# Causal association of leisure sedentary behavior and cervical spondylosis, sciatica, intervertebral disk disorders, and low back pain: a Mendelian randomization study

**DOI:** 10.3389/fpubh.2024.1284594

**Published:** 2024-01-23

**Authors:** Youjia Qiu, Xingzhou Wei, Yuchen Tao, Bingyi Song, Menghan Wang, Ziqian Yin, Minjia Xie, Aojie Duan, Zhouqing Chen, Zhong Wang

**Affiliations:** ^1^Department of Neurosurgery & Brain and Nerve Research Laboratory, The First Affiliated Hospital of Soochow University, Suzhou, Jiangsu, China; ^2^Suzhou Medical School of Soochow University, Suzhou, Jiangsu, China

**Keywords:** leisure sedentary behavior, sciatica, cervical spondylosis, intervertebral disk disorders, low back pain, Mendelian randomization

## Abstract

**Background:**

Some studies suggest sedentary behavior is a risk factor for musculoskeletal disorders. This study aimed to investigate the potential causal association between leisure sedentary behavior (LSB) (including television (TV) viewing, computer use, and driving) and the incidence of sciatica, intervertebral disk degeneration (IVDD), low back pain (LBP), and cervical spondylosis (CS).

**Methods:**

We obtained the data of LSB, CS, IVDD, LBP, sciatica and proposed mediators from the gene-wide association studies (GWAS). The causal effects were examined by Inverse Variance Weighted (IVW) test, MR-Egger, weighted median, weighted mode and simple mode. And sensitivity analysis was performed using MR-Pleiotropy Residual Sum and Outlier (MR-PRESSO) and MR-Egger intercept test. Multivariable MR (MVMR) was conducted to investigate the independent factor of other LSB; while two-step MR analysis was used to explore the potential mediators including Body mass index (BMI), smoking initiation, type 2 diabetes mellitus (T2DM), major depressive disorder (MDD), schizophrenia, bipolar disorder between the causal association of LSB and these diseases based on previous studies.

**Results:**

Genetically associated TV viewing was positively associated with the risk of CS (OR = 1.61, 95%CI = 1.25 to 2.07, *p* = 0.002), IVDD (OR = 2.10, 95%CI = 1.77 to 2.48, *p* = 3.79 × 10^−18^), LBP (OR = 1.84, 95%CI = 1.53 to 2.21, *p* = 1.04 × 10^−10^) and sciatica (OR = 1.82, 95% CI = 1.45 to 2.27, *p* = 1.42 × 10^−7^). While computer use was associated with a reduced risk of IVDD (OR = 0.66, 95%CI = 0.55 to 0.79, *p* = 8.06 × 10^−6^), LBP (OR = 0.49, 95%CI = 0.40 to 0.59, *p* = 2.68 × 10^−13^) and sciatica (OR = 0.58, 95%CI = 0.46 to 0.75, *p* = 1.98 × 10^−5^). Sensitivity analysis validated the robustness of MR outcomes. MVMR analysis showed that the causal effect of TV viewing on IVDD (OR = 1.59, 95%CI = 1.13 to 2.25, *p* = 0.008), LBP (OR = 2.15, 95%CI = 1.50 to 3.08, *p* = 3.38 × 10^−5^), and sciatica (OR = 1.61, 95%CI = 1.03 to 2.52, *p* = 0.037) was independent of other LSB. Furthermore, two-step MR analysis indicated that BMI, smoking initiation, T2DM may mediate the causal effect of TV viewing on these diseases.

**Conclusion:**

This study provides empirical evidence supporting a positive causal association between TV viewing and sciatica, IVDD and LBP, which were potentially mediated by BMI, smoking initiation and T2DM.

## 1 Introduction

Cervical spondylosis (CS) is a degenerative condition characterized by the compression of the cervical spinal cord and/or surrounding blood vessels and has been shown to be associated with musculoskeletal neck disorders (1, 2). More than one third of the global population experiences mechanical neck pain for a duration of at least 3 months (3). In addition, prolonged neck flexion is a significant contributing factor in the development of myofascial neck pain (4). Intervertebral disk disorders (IVDD) is a common musculoskeletal condition and age-related degenerative disorder in which the amounts of proteoglycans and water in the nucleus pulposus within the disk gradually decreases (5–7). With increasing age, intervertebral disks gradually lose flexibility, elasticity and shock absorbency due to the degeneration, and the fibrosis surrounding the disks can become fragile and prone to rupture (8). The primary clinical manifestation of IVDD is usually low back pain (LBP) and can lead to radiculopathy and myelopathy (9, 10). Sciatica is considered a symptom, rather than a specific disease diagnosis, resulting from the inflammation or compression of the lumbosacral nerve roots L4-S1 by IVDD (11, 12). Research has shown a substantial range in the occurrence of sciatica symptoms, with prevalence rates ranging from 1.6 to 43% (13). In addition, several systematic reviews have suggested that smoking, obesity, and engaging in physically demanding work are potential risk factors for the initial onset of sciatica (14). LBP is not a distinct disease but rather a symptom characterized by pain in the dorsal region between the lower ribs and the gluteal fold (15). A systematic review found that the prevalence ranged from 1.4 to 20.0% in North America, Northern Europe, and Israel (16).

Leisure sedentary behavior (LSB) encompasses activities that involve maintaining a reclined or seated position, leading to limited physical exertion and low metabolic activity (energy expenditure ≤1.5 metabolic equivalents) (17). Such activities include watching television (TV), using a computer, and driving (18). Studies have demonstrated a link between LSB and an increased risk of cardiovascular disease, all-cause mortality, metabolic syndrome, and obesity (19, 20). Additionally, a large cohort study suggests an association between prolonged sedentary leisure time exceeding 6 h and an increased likelihood of neurological, sensory, and musculoskeletal disorders (21). Furthermore, a meta-analysis showed that there may be association between full-day sedentary or sitting time and the risk of cervical, and shoulder pain and LBP (22). However, due to the deficits in potential confounding factors and reverse causality, the precise understanding of the relationship between LSB and sciatica, CS, IVDD, and LBP remains incomplete (21, 23).

Mendelian Randomization (MR) analysis is an analytical method to evaluate the causal effect of specific exposures on outcomes using genetic variants available on genome-wide association study (GWAS) (24). GWAS is a systematic analysis of genes which examines and identify DNA sequence variations regulate a complex trait or affect the risk of the disease (25, 26). Previous observational studies of disk disease are subject to unavoidable potential confounding factors such as heterogeneity of the included studies, individual factors in the study population, investigator subjectivity and measurement error, as well as reverse causation due to the effect of disease phenotypes on exposures during disease progression (27–30). As single nucleotide polymorphisms (SNPs) are randomly assigned at conception, they are unlikely to be influenced by lifestyle and environmental factors (31). This feature of MR reduces the risk of confounding factors and reverse causal association, which is common in observational studies (32). Therefore, using a two-sample MR design to analyze summary statistics from GWAS could increase the statistical efficacy of causal association (33). Multivariable Mendelian Randomization (MVMR) analysis is a further developed exploration of traditional MR, which could evaluate more than two exposures simultaneously, and assess the causal association after adjusting other exposures (34). This study aimed to assess the causal associations between LSB (TV viewing, computer use, and driving) and CS, IVDD, LBP, and sciatica with MR approach. Then we further investigated the potential factor independent of other LSB. Several previous studies suggested there may exist an association between LSB and body mass index (BMI), type 2 diabetes mellitus (T2DM) and smoking (35, 36). In addition, LSB may be risk factors for neuropsychiatric disorders (37) and a recent MR study confirmed that LSB (TV viewing) is a high risk factor for major depressive disorder (MDD) (38). In addition, previous epidemiological studies on risk factors of these musculoskeletal disease indicated that several lifestyle related factors may also associated with these diseases, such as depression, education, smoking, obesity, and physical activities (39–41). These modifiable risk factors may also play a role between the LSB and CS, IVDD, LBP, and sciatica. Mediation analyses using two-step MR method could identify the causal pathways through which exposure affects outcomes and their relative importance, which can help identify which factors mediate the relationship between exposure and outcome, which in turn can be intervened or prevented to reduce the impact of exposure on outcomes (42). Therefore, we also evaluated the potential mediator between the causal association of LSB and these skeletomuscular diseases, which will help to optimize disease prevention at both clinical and health levels.

## 2 Methods

### 2.1 Ethical statement

Our study is a re-analysis of data already included in GWAS; all ethical approvals have been obtained by the original GWAS authors. Thus, no additional ethical approval is required.

### 2.2 Study design

**Figure 1:**
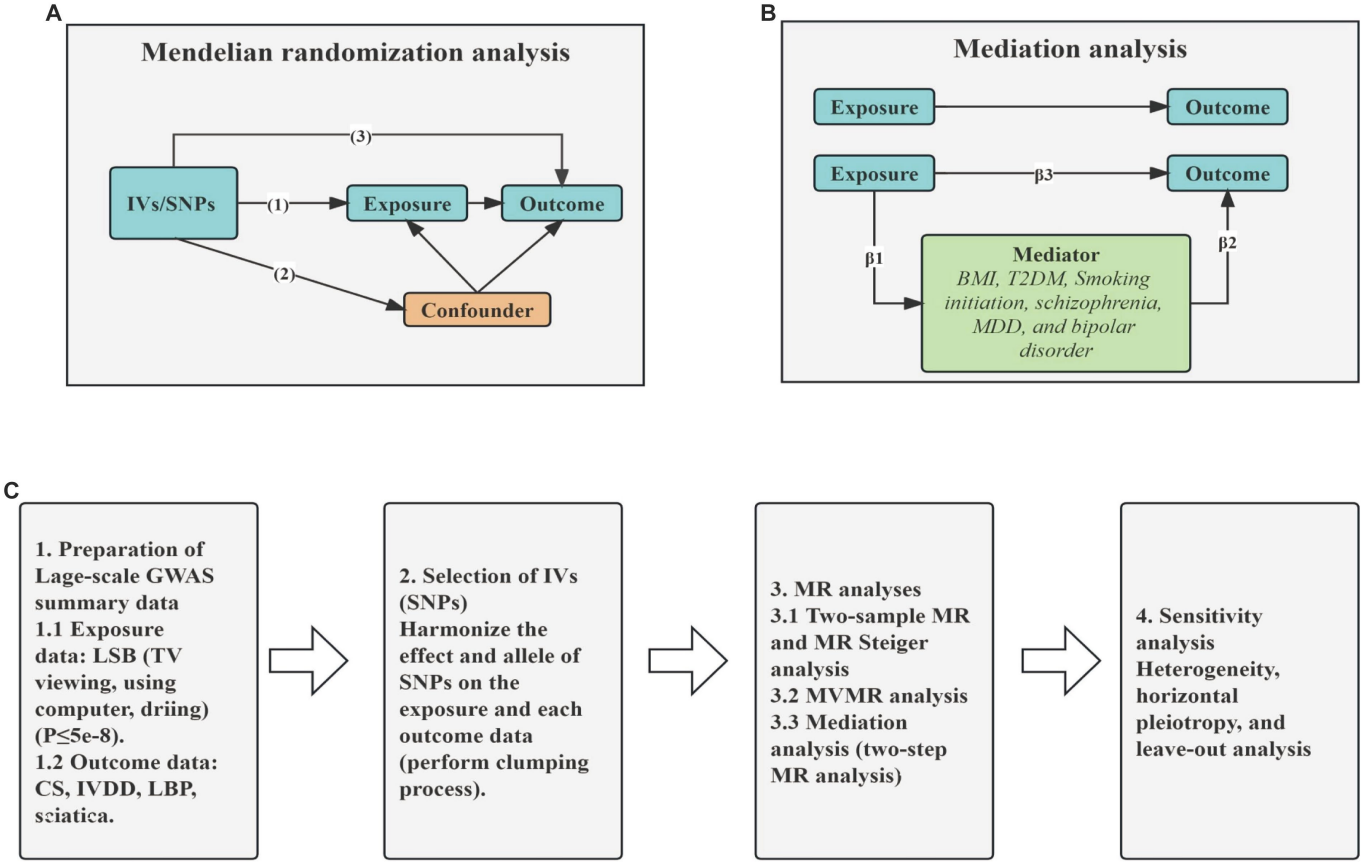
Graphical overview of the MR process. **(A)** The principles for two-sample MR analysis. **(B)** The principles for two-step MR analysis. **(C)** The whole workflow of MR analysis. IVs, instrumental variables; SNPs, single nucleotide polymorphisms; BMI, Body mass index; T2DM, type 2 diabetes mellitus; MDD, major depressive disorder; TV, television; CS, cervical spondylosis; IVDD, intervertebral disk disorders; LBP, low back pain; MR, Mendelian Randomization; MVMR, Multivariable Mendelian Randomization.

In this study, we used MR analysis to detect the association between LSB (TV viewing, computer use and driving) and CS, IVDD, LBP and sciatica using publicly available datasets from large GWAS. We used strict selection criteria to identify SNPs associated with specific LSB (including prolonged TV viewing, computer use, and driving), which were subsequently used as instrumental variables (IVs). The MR design was based on the following assumptions: (1) the genetic variants were directly and robustly associated with LSB and met the GWAS significance threshold; (2) the genetic variants used were not linked to any confounders; (3) the selected genetic variants influenced the development of CS and sciatica only through LSB. We used MR Steiger analysis to determine the precision of the direction. In addition, we also investigated the independent causal role of an exposure after adjusting for other exposures using MVMR. Furthermore, we sought to further explore the potential mechanisms by which genetic proxies for LSB influence susceptibility to CS, IVDD, sciatica and LBP through assessing the effects of potential mediating risk factors (including BMI, T2DM smoking initiation, MDD, schizophrenia, bipolar disorder) using two-step MR analysis (Figure 1). Confounding factors including alcohol use, smoking, low density lipoprotein, triglyceride, etc. are unrelated to genetic variation (43).

### 2.3 Data source

The GWAS summary data of LSB was obtained from a previous publication of the UK Biobank Repository (*N* = 422,218; European ancestry) (43). The study conducted a GWAS of sedentary behavior in 422,218 individuals of European origin. We selected IVs significantly associated (*p* < 5 × 10^−8^) with LSB on the UK Biobank website,^1^ including TV viewing, computer use, and driving. The amount of time respondents spent on these three behaviors was measured by their responses to the following questions: “In a typical day, how many hours do you spend watching television?,” “In a typical day, how many hours do you spend using a computer (excluding using a computer at work)?” and “In a typical day, how many hours do you spend driving?.” The mean age of the cohort at first assessment was 57.4 years (SD 8.0), and 45.7% of the study population was male. The average daily TV viewing was 2.8 h, computer use was 1.0 h and driving was 0.9 h, with standard deviations (SD) of 1.5 h, 1.2 h and 1.0 h, respectively. A strict threshold (*R*^2^ < 0.001, kb = 10,000) clustering procedure was used to ensure the independence of the selected SNPs. SNPs with a significant association with outcomes (*p* < 5 × 10^−8^) were also diskarded, and the mean F-statistic of all included exposures was greater than 10 (24).

Summary level GWAS results for CS (*N* = 284,358), IVDD (*N* = 308,600), sciatica (*N* = 377,277), and LBP (*N* = 300,293) were obtained from the Finn-Gen, and participant details, statistical protocols, and genetic information are available on the website.^2^ The trait of CS was labeled as “Cervical disk disorders” (44).

We also obtained genetic association for potential mediator (such as BMI, smoking initiation and T2DM) from different database in the IEU open GWAS,^3^ and the detailed information of the source of mediators is shown in Table 1. We selected phenotypes of potential mediators with a non-overlapped population to minimize the bias of weak instruments caused by sample overlap.

**Table 1:** Data source of the exposures and outcomes.

Traits	Consortium	Sample size	Ancestry	Author	Year of publication
Time spent watching TV	UK Biobank	437,877	European	Ben Elsworth	2018
Time spent using computer	UK Biobank	360,895	European	Ben Elsworth	2018
Time spent driving	UK Biobank	310,555	European	Ben Elsworth	2018
CS	FinnGen	284,358	European	NA	NA
IVDD	FinnGen	308,600	European	NA	NA
LBP	FinnGen	300,293	European	NA	NA
Sciatica	FinnGen	289,533	European	NA	NA
BMI	UK Biobank	461,460	European	Ben Elsworth	2018
Smoking initiation	GSCAN	607,291	European	Liu M	2019
Type 2 diabetes	NA	655,666	European	Xue A	2018
Major depressive disorder	PGC	173,005	European	Wray	2018
Schizophrenia	PGC	127,906	European	Trubetskoy V	2022
Bipolar disorder	PGC	413,466	European	Niamh Mullins	2021

TV, television; CS, cervical spondylosis; IVDD, interverbal disk disorder; LBP, low back pain; BMI, Body mass index; GSCAN, Sequencing Consortium of Alcohol and Nicotine use; ICBP, International Consortium of Blood Pressure; PGC, Psychiatric Genomics Consortium.

### 2.4 Statistical analysis

Inverse variance method (IVW) was used as the primary method for MR analysis. Random-effects IVW was used when significant heterogeneity (*I*^2^ > 50%) was detected, otherwise fixed-effects IVW was used. MR-Egger, weighted median, MR Pleiotropy Residuals and Outliers (MR-PRESSO) were also applied for additional statistical analysis. MR-Egger can detect and correct for potential horizontal pleiotropy, but results may be affected by the presence of outlying genetic variables (45–47). Moreover, MR-Egger slopes are relatively effective as estimates of MR in the presence of horizontal pleiotropy. The weighted median method ensures the stability of causality estimates by eliminating errors in the presence of 50% invalid IVs, and may provide better causality detection than the MR-egger under certain conditions (48, 49). MR Steiger directionality test was performed to rule out possibilities of reverse causal association. The estimates are provided for each increase of one standard deviation (SD), and the impact magnitude was reported as the odds ratio (OR) along with a 95% confidence interval (95%CI). Finally, various diagnostic plots were used to detect the robustness of MR estimates. The scatter plots showcase the association of SNPs with exposure and outcome, while the forest plots illustrate the influence of individual instrumental variable on the overall estimation of causality (50). Leave-one-out plots were utilized to visually present the findings of leave-one-out analysis, which involved recalculating the causal estimates obtained from IVW by excluding one SNP at a time. This approach was carried out to assess whether the estimates were affected by biases or driven by outliers (45).

The MR-PRESSO method identifies and corrects outliers by detecting the presence of horizontal multi-effects through global tests, outlier tests and bias tests (51). It is identified horizontal pleiotropy that genetic variants associated with the exposure (LSB) of interest have a direct effect on the outcome (CS, IVDD, sciatica and LBP) through multiple pathways other than the hypothesized exposure, and if horizontal pleiotropy occurs in MR analyses, the results of MR analyses will become unreliable (52). Cochran’s Q test and *I*^2^ statistics were carried out to evaluate the heterogeneity of the instrumental genetic variable, and a *p*-value <0.05 indicated significant heterogeneity. MR-Egger intercept, ME-PRESSO global test were performed to assess pleiotropy between IVs. Directional pleiotropy was assessed using the intercept term in MR Egger regressions, while in the MR-PRESSO method, heterogeneity is minimized by finding and removing outliers, then reassessing causal estimates. When horizontal pleiotropy still existed, we used the Radial MR method to filter variants identified as outliers (53). In addition, Bonferroni test was used for multiple comparisons, and a *p*-value of 0.016 (0.05/3 exposures) was considered significant, *p*-value ranged from 0.016 to 0.05 was considered suggested significant. In addition, *p*-value <0.05 was considered statistically significant in MVMR, which did not involve errors in multiple comparison.

We used MVMR to further assess the independent effects of these three LSB on these outcomes. MVMR can be used to assess which characteristics maintain causal relationships with outcomes, reflecting the direct effects of exposure on the outcome (54, 55). MR responds to the total effect of exposure and outcome and is composed of both direct (MVMR) and indirect effects (mediation effects) (42, 56). In addition, we further explored the effect of potential mediators that may mediate the causality between LSB and these outcomes using two-step MR analysis. The effect of LSB on these outcomes after adjusting for potential mediators is referred to as the direct effect, whereas the effect mediated by potential mediators is referred to as the indirect effect. In two-step MR, the first step is to test the influence of LSB on potential mediators; the second step is to test the influence of potential mediators on CS, IVDD, LBP, and sciatica. All statistical analyses were two-sided. The following packages were all used in R software (version 4.3.0) for analyses: MendelR (version 7.6.2), RadialMR, MR-PRESSO (1.0), and Forestploter (1.1.0) packages.

## 3 Result

### 3.1 Two-sample MR

All instrumental variables used to genetically proxy LSB are shown in Supplementary Table S1, with a total of 113 SNPs for TV viewing, 83 SNPs for computer use, and 7 SNPs for driving. Meanwhile, Steiger filter test showed no reverse causality between the exposure and outcome (Supplementary Table S2). The results of MR estimates are shown in Supplementary Table S3. However, MR-PRESSO and radial MR test detected some of outliers, we therefore preformed MR analysis after remove these outliers (Supplementary Tables S4, S5). The number of eventually enrolled SNPs are shown in Supplementary Table S6.

**Figure 2:**
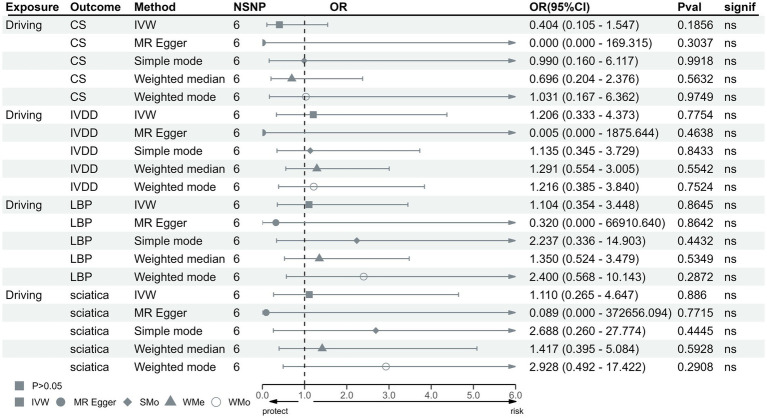
MR analysis for time spent driving on outcomes. MR, Mendelian Randomization; IVW, invers variance weighted; CS, cervical spondylosis; IVDD, intervertebral disk disorders; LBP, low back pain; OR, odds ratio; CI, confidence interval.

**Figure 3:**
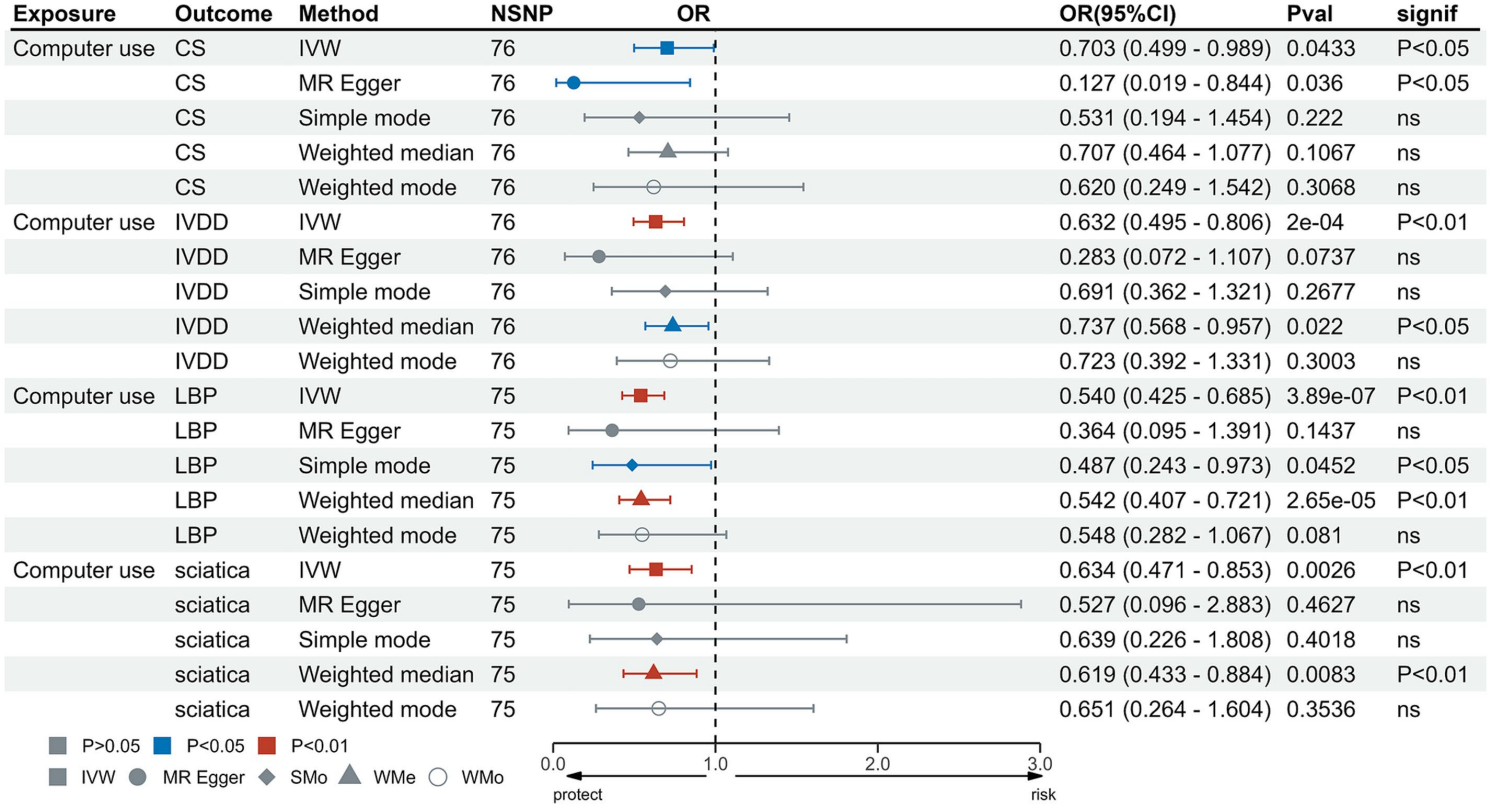
MR analysis for time spent using computer on outcomes. MR, Mendelian Randomization; IVW, inverse variance weighted; CS, cervical spondylosis; IVDD, intervertebral disk disorders; LBP, low back pain; OR, odds ratio; CI, confidence interval.

**Figure 4:**
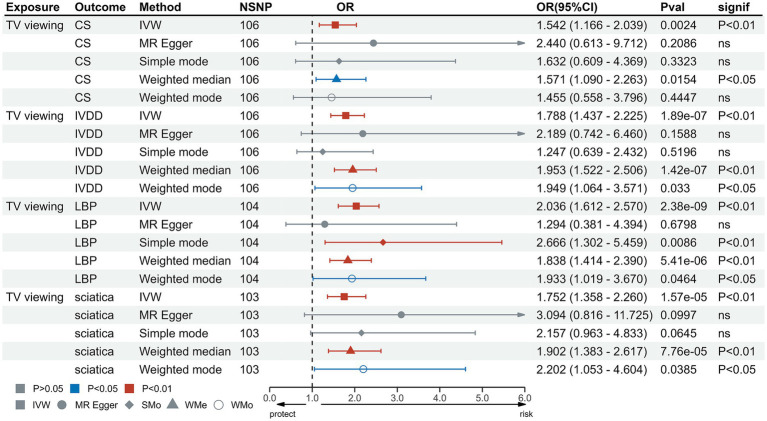
MR analysis for TV viewing on outcomes. MR, Mendelian Randomization; IVW, inverse variance weighted; TV, television; CS, cervical spondylosis; IVDD, intervertebral disk disorders; LBP, low back pain; OR, odds ratio; CI, confidence interval.

In the IVW test, no significant causal relationship was found between computer use and CS (OR = 0.80, 95%CI = 0.61 to 1.05, *p* = 0.11). There were also no significant causal associations between driving and CS (OR = 0.40, 95%CI = 0.11 to 1.55, *p* = 0.19), IVDD (OR = 1.67, 95%CI = 0.66 to 4.22, *p* = 0.28), sciatica (OR = 1.73, 95%CI = 0.44 to 6.71, *p* = 0.43) and LBP (OR = 1.38, 95%CI = 0.67 to 2.83, *p* = 0.38) (Figure 2). However, genetically predicted computer use was associated with a reduced risk of IVDD (OR = 0.66, 95%CI = 0.55 to 0.79, *p* = 8.06 × 10^−6^), sciatica (OR = 0.58, 95%CI = 0.46 to 0.75, *p* = 1.98 × 10^−5^) and LBP (OR = 0.49, 95%CI = 0.40 to 0.59, *p* = 2.68 × 10^−13^) (Figure 3); while genetically predicted TV viewing was positively associated with the risk of CS (OR = 1.61, 95%CI = 1.25 to 2.07, *p* = 0.002), IVDD (OR = 2.10, 95%CI = 1.77 to 2.48, *p* = 3.79 × 10^−18^), sciatica (OR = 1.82, 95%CI = 1.45 to 2.27, *p* = 1.42 × 10^−7^) and LBP (OR = 1.84, 95%CI = 1.53 to 2.21, *p* = 1.04 × 10^−10^) (Figure 4). In addition, weight median and other methods (weight mode and MR-Egger) also provided consistent results with IVW, showing the same directions, indicating the robustness of the identified SNP.

Heterogeneity test results were consistent with a value of *p* >0.05 except for driving and CS (*p* < 0.05) (Supplementary Table S7). The leave-one-out test suggested no SNP derived the causal association between exposure and outcome (Supplementary Figures S1–S12). Furthermore, scatter plot showed the same direction of different methods (Supplementary Figures S13–S24), and the funnel plot displayed a mostly symmetrical distribution (Supplementary Figures S25–S36), indicating the robustness of MR results. Funnel plot showed no specific outlier among SNPs. The results of the MR-PRESSO test after removing outliers showed no potential pleiotropy in MR analysis (Supplementary Table S8). The results of forest plot are shown in Supplementary Figures S37–S48.

### 3.2 Multivariable Mendelian randomization and mediation analysis

**Figure 5:**
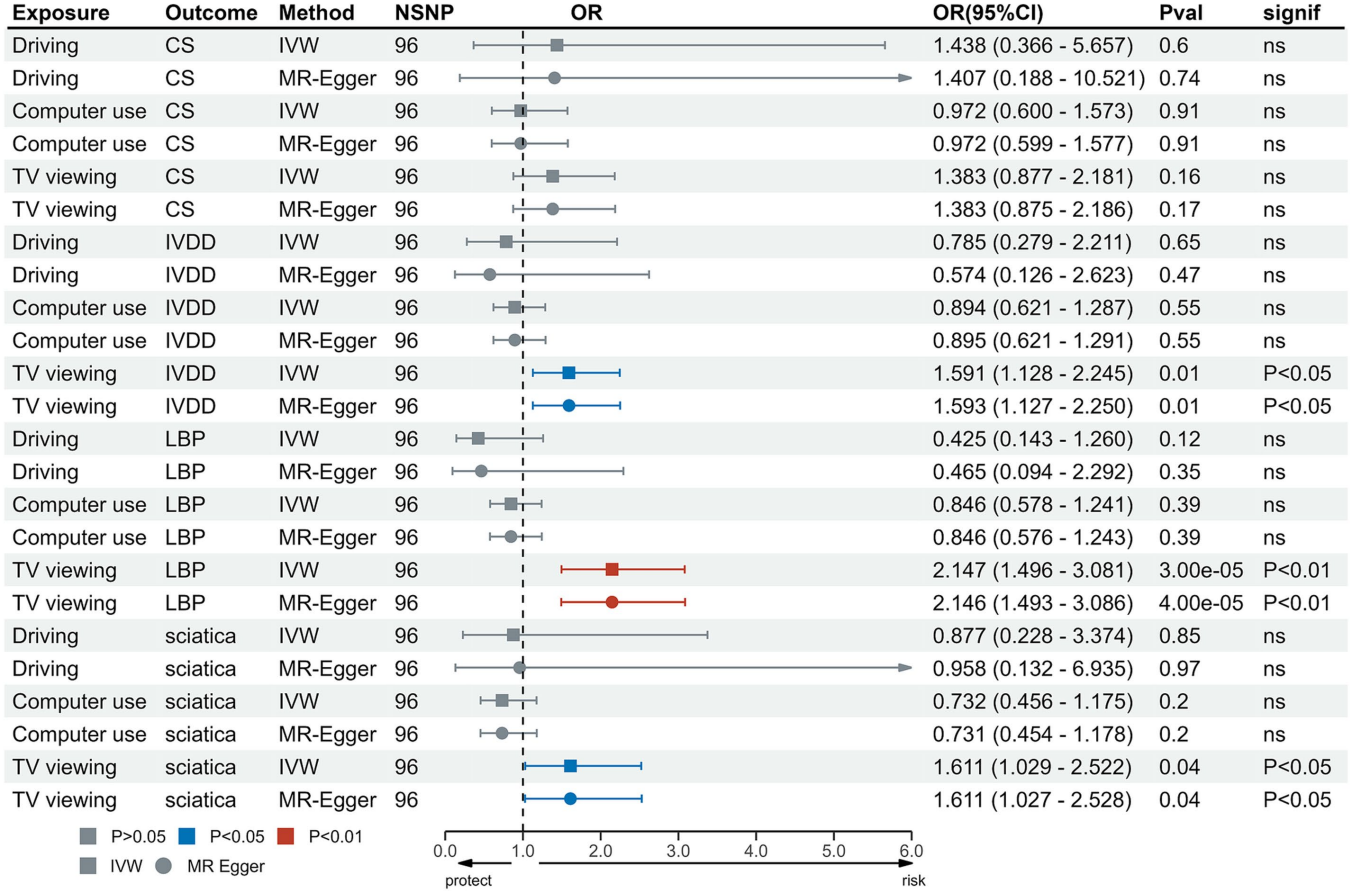
MVMR analysis for TV viewing on outcomes. MVMR, Multivariable Mendelian Randomization; IVW, inverse variance weighted; TV, television; CS, cervical spondylosis; IVDD, intervertebral disk disorders; LBP, low back pain; OR, odds ratio; CI, confidence interval.

MVMR was used to evaluate the independent effects of these three LSB on these outcomes. The results of the MVMR analysis showed that TV viewing remained independently causally associated with IVDD (OR = 1.59, 95%CI = 1.13 to 2.25, *p* = 0.008), LBP (OR = 2.15, 95%CI = 1.50 to 3.08, *p* = 3.38 × 10^−5^) and sciatica (OR = 1.61, 95%CI = 1.03 to 2.52, *p* = 0.037). However, MVMR analysis demonstrated that TV viewing was not significantly causally related to CS (OR = 1.38, 95%CI = 0.88 to 2.18, *p* = 0.16) and computer use was not significantly causally related to CS (OR = 0.97, 95%CI = 0.60 to 1.57, *p* = 0.91), IVDD (OR = 0.89, 95%CI = 0.62 to 1.29, *p* = 0.55), LBP (OR = 0.85, 95%CI = 0.58 to 1.24, *p* = 0.39) and sciatica (OR = 0.73, 95%CI = 0.46 to 1.18, *p* = 0.20) (Table 2 and Figure 5).

**Table 2:** Result of MVMR analysis between LSB and outcome.

Exposure	Outcome	SNPs	IVW method		MR Egger method		MR Egger intercept		
			OR	*p-*value	OR	*p*-value	Intercept	SE	*p*-value
Time spent driving	CS	96	1.44 (0.37 to 0.6)	0.6	1.41 (0.19 to 10.52)	0.74	0.00008	0.003	0.98
Time spent using computer	CS	96	0.97 (0.6 to 1.57)	0.91	0.97 (0.6 to 1.58)	0.91			
Time spent watching TV	CS	96	1.38 (0.88 to 2.18)	0.16	1.38 (0.87 to 2.19)	0.17			
Time spent driving	IVDD	96	0.78 (0.28 to 2.21)	0.65	0.57 (0.13 to 2.62)	0.47	0.001	0.002	0.58
Time spent using computer	IVDD	96	0.89 (0.62 to 1.29)	0.55	0.9 (0.62 to 1.29)	0.55			
Time spent watching TV	IVDD	96	1.59 (1.13 to 2.25)	0.008	1.59 (1.13 to 2.25)	0.01			
Time spent driving	LBP	96	0.43 (0.14 to 1.26)	0.12	0.46 (0.09 to 2.29)	0.35	−0.0003	0.002	0.88
Time spent using computer	LBP	96	0.85 (0.58 to 1.24)	0.39	0.85 (0.58 to 1.24)	0.39			
Time spent watching TV	LBP	96	2.15 (1.05 to 2.08)	0.00003	2.15 (1.49 to 3.09)	0.00004			
Time spent driving	sciatica	96	0.88 (0.23 to 3.37)	0.85	0.96 (0.13 to 6.94)	0.97	−0.0003	0.003	0.9
Time spent using computer	sciatica	96	0.73 (0.46 to 1.18)	0.2	0.73 (0.45 to 1.18)	0.2			
Time spent watching TV	sciatica	96	1.61 (1.03 to 2.52)	0.04	1.61 (1.03 to 2.53)	0.04			

MVMR, Multivariable Mendelian Randomization; TV, television; CS, cervical spondylosis; IVDD, interverbal disk disorder; LBP, low back pain.

**Figure 6:**
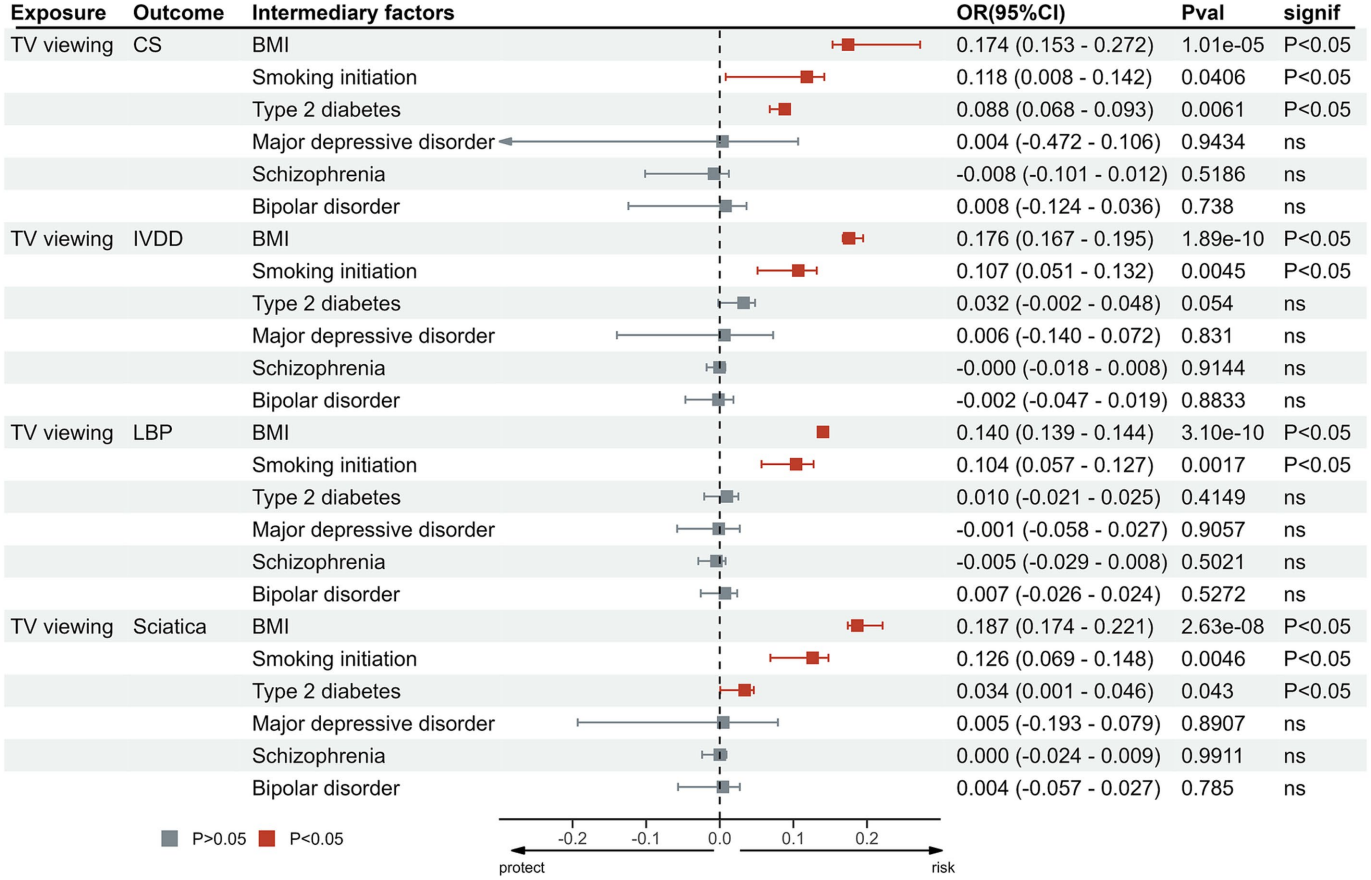
Proportion of the effect of TV viewing on outcomes mediated by potential mediators. MR, Mendelian Randomization; IVW, inverse variance weighted; TV, television; CS, cervical spondylosis; IVDD, intervertebral disk disorders; LBP, low back pain.

The proportion of mediation is shown in Table 3 and Figure 6. MDD (*p* > 0.05), schizophrenia (*p* > 0.05), bipolar disorder (*p* > 0.05) did not exhibit mediating effects between the relationship of LSB and these outcomes. In the relationship between TV viewing and CS, BMI (0.174), smoking initiation (0.118) and T2DM (0.088) were identified as potential intermediary factors. And in the relationship between TV viewing and IVDD, BMI (0.176), smoking initiation (0.107) and T2DM (0.062) were identified as factors. Meanwhile, BMI (0.14), smoking initiation (0.104) and T2DM (0.01) were found to mediate the effect of TV viewing on sciatica. In particular, BMI (0.187), smoking initiation (0.126) and T2DM (0.034) were also found to mediate the effect of TV viewing on LBP (Supplementary Table S9).

**Table 3:** Results of intermediary analyses for TV viewing and outcome.

Outcome	Intermediary factors	Beta	OR	95%CI	*p*-value	Proportion (%)
CS	BMI	0.08	1.08	1.04, 1.12	<0.001	17.4
CS	Smoking initiation	0.05	1.05	1.001, 1.11	0.04	11.8
CS	Type 2 diabetes	0.04	1.04	1.01, 1.07	0.006	8.8
CS	Major depressive disorder	0.002	1.002	0.93, 1.08	0.94	0.4
CS	Schizophrenia	−0.003	1.00	0.98, 1.009	0.52	−0.8
CS	Bipolar disorder	0.003	1.003	0.98, 1.03	0.74	0.8
IVDD	BMI	0.10	1.11	1.07, 1.14	<0.001	17.6
IVDD	Smoking initiation	0.06	1.06	1.02, 1.11	0.004	10.7
IVDD	Type 2 diabetes	0.02	1.02	0.999, 1.04	0.05	3.2
IVDD	Major depressive disorder	0.004	1.004	0.95, 1.06	0.83	0.6
IVDD	Schizophrenia	−0.0002	1.00	0.99, 1.006	0.91	−0.03
IVDD	Bipolar disorder	−0.001	1.00	0.98, 1.01	0.88	−0.2
LBP	BMI	0.10	1.10	1.07, 1.14	<0.001	14.0
LBP	Smoking initiation	0.07	1.08	1.03, 1.13	0.002	10.4
LBP	Type 2 diabetes	0.01	1.01	0.99, 1.02	0.41	1.0
LBP	Major depressive disorder	−0.0001	1.00	0.97, 1.03	0.91	−0.1
LBP	Schizophrenia	−0.003	1.00	0.99, 1.008	0.5	−0.5
LBP	Bipolar disorder	0.005	1.005	0.99, 1.02	0.53	0.7
Sciatica	BMI	0.10	1.11	1.07, 1.15	<0.001	18.7
Sciatica	Smoking initiation	0.07	1.07	1.02, 1.13	0.005	12.6
Sciatica	Type 2 diabetes	0.02	1.02	1.0002, 1.04	0.04	3.4
Sciatica	Major depressive disorder	0.003	1.00	0.94, 1.07	0.89	0.5
Sciatica	Schizophrenia	2.47	1.00	0.99, 1.007	0.99	0.004
Sciatica	Bipolar disorder	0.002	1.002	0.98, 1.02	0.78	0.4

TV, television; CS, cervical spondylosis; IVDD, interverbal disk disorder; LBP, low back pain; BMI, Body mass index.

## 4 Discussion

Our MR analysis suggested that LSB (TV viewing) may serve as a significant risk factor that is causally related to the development of IVDD, sciatica, and LBP. Additionally, factors such as BMI, T2DM, and smoking initiation may act as potential mediators in the relationship between TV viewing and IVDD, sciatica, and LBP.

IVDD is a common musculoskeletal disease caused by degenerative changes in the nucleus pulposus of the intervertebral disk (7, 11, 57). And the main cause of LBP is IVDD and results from a variety of known or unknown pathologies or diseases (58, 59). While the etiology of sciatica is attributed to the involvement of the L4-S1 nerve roots by the IVDD (11). Additionally, patients with IVDD may experience LBP and sciatica as a result of inflammation caused by IVDD (7). One study showed that LSB was associated with an increased risk of IVDD and LBP (60) and Euro et al. found a significant association between sitting and the incidence of sciatica (61), which are consistent with our findings.

LSB (TV viewing) may promote the onset and progression of IVDD, sciatica, and LBP by altering disk biomechanical relationships and causing chronic disk inflammation, due to body weight gain. One of the main causes of disk degeneration is damage to the intervertebral disks caused by disturbed biomechanical relationships between the vertebrae (62), and vertebral endplate defects have been shown to be the primary cause of disk degeneration (63, 64). Several studies have demonstrated that LSB plays a role in the development of high BMI and obesity, as evidenced by research conducted by various authors (65–71). TV viewing is considered a ‘mentally passive’ behavior, whereas using a computer is considered a ‘mentally active’ behavior (38). Furthermore, as a ‘mentally passive’ behavior, TV viewing is often perceived as an immersive and less reflective form of leisure and entertainment (52) and unhealthy eating, alcohol consumption and snacking are also associated with TV viewing (72), which may lead to obesity by having more involuntary intake and less consumption compared to non-sedentary population. And obesity can lead to severe postural changes that affect joint loading, while prolonged TV viewing can lead to prolonged periods of poor posture due to ‘mentally passivity’, which increases spinal strain and muscle fatigue leading disturbed biomechanical relationships and vertebral endplate defects (73–75). In addition, an increase in BMI increases the lumbosacral angle, causing greater flexion of the sacroiliac joints, increased lumbar disk and joint torque, which in turn increases joint loading and causes LBP (76, 77). At the same time, high BMI and obesity lead to metabolic dysregulation and chronic low-grade inflammatory response causing abnormal cytokine production, increased acute phase reactants and activation of inflammatory signaling pathways (78, 79). Moreover, fatty tissue has been shown to promote an inflammatory response through the release of leptin and resistin (80–82). Vertebral endplate defects may allow pro-inflammatory mediators which may be caused by obesity to be transported from the disk to the vertebral body, which in turn cause degenerative disk changes (63).

It has been shown that smoking is associated with LSB using MR analysis (52), and several studies have shown that smoking is an adverse risk factor for LBP and sciatica (83–85), which are consistent with the results of our mediation analysis. By reducing the blood supply to the intervertebral disks, smoking can cause intervertebral disk dystrophy (84), and tobacco smoke has been shown to contribute to degenerative changes in intervertebral disks in animal models (86). In addition, tobacco smoke inhalation promotes the production and release of cytokines from inflammatory cells in the intervertebral disk, which causes disk fibrosis and interferes with disk healing and repair (87, 88). Smoking may cause elevated serum levels of advanced glycation end-products (AGEs) and elevated AGEs promote degenerative disk changes by promoting nucleus pulposus apoptosis, facilitating collagen degradation of the annulus fibrosus, and inducing endplate sclerosis (89). In addition, T2DM has also been shown to be causally associated with an increased risk of IVDD (90, 91). In patients with T2DM mellitus, hyperglycemia causes the irreversible formation and accumulation of glycosylation end products, leading to pathophysiological changes in the cartilaginous endplates of the intervertebral disks, while at the same time hyperglycemia affects disk nutrition, cell viability and matrix homeostasis, resulting in changes in disk biomechanics and ultimately leading to IVDD (90). Diabetes accelerates hyperglycemia-induced accumulation of AGEs (92) and the continued accumulation of AGEs associated with hyperglycemia in T2DM was responsible for disk stiffening and the subsequent destructive chain of events (93). Moreover, LSB is considered a potential risk factor for neuropsychiatric disorders (37), and TV viewing was regard as a ‘mentally passive’ behavior which was considered that associated with an increased MDD risk (38). However, through mediation analyses, we did not find a mediating role for these psychiatric disorders (schizophrenia, MDD and bipolar disorder) between TV viewing and CS, IVDD, LBP, and sciatica. This may be due to the fact that we used mediation analyses (two-step MR analysis) to examine the potential relationship and role of neuropsychiatric disorders in the relationship between LSB (TV viewing) and those outcomes.

In addition, the mechanisms underlying neck pain and CS have been identified as increased intramuscular pressure in the neck, abnormal fascial tension on peripheral nerves, and altered muscle tissue mechanics (2, 4, 94). This condition is primarily caused by increased stress on the intervertebral disks in the neck and a decrease in flexion strength, resulting in the splitting of the annulus and subsequent herniation of the nucleus pulposus (cervical disk herniation), which in turn compresses the spinal cord and blood vessels (95). And studies have shown that inactivity and occasional sitting are associated with more perceived neck pain (96, 97). Although the results of two-sample MR analysis showed a significant association between TV viewing and CS, the results of our MVMR analysis showed no significant association between LSB and CS, which is inconsistent with the expected result. This suggests that the LSB of TV viewing does not have an effect on CS independently of other sedentary behaviors.

As mentioned above, although several observational studies have suggested an association between LSB and CS, IVDD, LBP, and sciatica, they have not provided clear evidence of a causal relationship. In addition, strong evidence of an association may not be available due to confounding variables, reverse causality and survival bias. We therefore performed MR analysis of sedentary behavior and CS, IVDD, LBP, and sciatica to resolve this uncertainty. Our study demonstrated that using computer and TV viewing are causally related to the aforementioned musculoskeletal disease, which should be emphasized in the preventive strategies. At the same time, avoiding smoking, maintaining a healthy BMI and preventing the onset of T2DM are also associated with avoiding these musculoskeletal diseases.

Our study has several strengths. First, the causal effect of LSB on IVDD, sciatica, and LBP was investigated using a large, publicly available GWAS database, the results were less likely to be influenced by confounding factors and reverse causality. Second, the population we selected was restricted to European origin to reduce the bias introduced by population stratification. Third, we further assessed the existence possible mediators in the relationship between LSB (TV viewing) and these outcomes, which may play a role in the prevention of these diseases. Fourth, the data sources for the mediators we analyzed were different from the exposures and outcomes, which effectively avoided the problem of overlapping sample sizes. However, there are some limitations to this study. Firstly, we only enrolled European ancestry because there is no GWAS of other origins with large sample size. Therefore, this result may not apply to other ancestries. Second, we did not conduct subgroup analysis on different sex due to the application of summary statistics instead of individual-level data. Additionally, although mediation analysis was conducted and mediators were identified, some other potential mediators, such as poor regions and chances of medical care, need to be heritable and available in GWAS.

## 5 Conclusion

In a summary, our two-sample MR study provides evidence that LSB is associated with the risk of CS, IVDD, sciatica, and LBP, and the causal effect of TV viewing on these diseases was independent of other LSB factors. In addition, mediation analysis indicated that BMI, smoking initiation, and T2DM may mediate the causal associations of TV viewing with IVDD, sciatica and LBP. These modifiable risk factors were the promising interventions for reducing the risk of these diseases.

## Data availability statement

The original contributions presented in the study are included in the article/Supplementary material, further inquiries can be directed to the corresponding authors.

## Ethics statement

The studies involving humans were approved by original GWAS authors. Thus, no additional ethical approval is required. The studies were conducted in accordance with the local legislation and institutional requirements. Written informed consent for participation was not required from the participants or the participants’ legal guardians/next of kin in accordance with the national legislation and institutional requirements.

## Author contributions

YQ: Conceptualization, Investigation, Validation, Writing – original draft, Writing – review & editing. XW: Conceptualization, Investigation, Validation, Writing – original draft, Writing – review & editing. YT: Conceptualization, Investigation, Validation, Writing – original draft. BS: Formal analysis, Methodology, Resources, Software, Writing – original draft. MW: Formal analysis, Methodology, Resources, Software, Writing – original draft. ZY: Data curation, Formal analysis, Methodology, Software, Writing – original draft. MX: Data curation, Methodology, Software, Writing – review & editing. AD: Data curation, Methodology, Supervision, Writing – original draft. ZC: Funding acquisition, Supervision, Writing – review & editing. ZW: Conceptualization, Supervision, Writing – review & editing.

## Footnotes

^1^ https://www.ukbiobank.ac.uk/

^2^ https://www.finngen.fi/en

^3^ https://gwas.mrcieu.ac.uk/
