# Supplementary material for: Causal association of leisure sedentary behavior and cervical spondylosis, sciatica, intervertebral disk disorders, and low back pain: a Mendelian randomization study
Source: Front Public Health. 2024 Jan 23;12:1284594. doi: 10.3389/fpubh.2024.1284594 (PMC10844448; doi:10.3389/fpubh.2024.1284594)

**Figure summary of supplementary information**

Figure S1: Leave-one-out plot for the effect of time spent driving on CS.

Figure S2: Leave-one-out plot for the effect of time spent driving on IVDD.

Figure S3: Leave-one-out plot for the effect of time spent driving on LBP.

Figure S4: Leave-one-out plot for the effect of time spent driving on sciatica.

Figure S5: Leave-one-out plot for the effect of time spent using computer on CS.

Figure S6: Leave-one-out plot for the effect of time spent using computer on IVDD.

Figure S7: Leave-one-out plot for the effect of time spent using computer on LBP.

Figure S8: Leave-one-out plot for the effect of time spent using computer on sciatica.

Figure S9: Leave-one-out plot for the effect of time spent watching TV on CS.

Figure S10: Leave-one-out plot for the effect of time spent watching TV on IVDD.

Figure S11: Leave-one-out plot for the effect of time spent watching TV on LBP.

Figure S12: Leave-one-out plot for the effect of time spent watching TV on sciatica.

Figure S13: Scatter plot for the effect of time spent driving on CS.

Figure S14: Scatter plot for the effect of time spent driving on IVDD.

Figure S15: Scatter plot for the effect of time spent driving on LBP.

Figure S16: Scatter plot for the effect of time spent driving on sciatica.

Figure S17: Scatter plot for the effect of time spent using computer on CS.

Figure S18: Scatter plot for the effect of time spent using computer on IVDD.

Figure S19: Scatter plot for the effect of time spent using computer on LBP.

Figure S20: Scatter plot for the effect of time spent using computer on sciatica.

Figure S21: Scatter plot for the effect of time spent watching TV on CS.

Figure S22: Scatter plot for the effect of time spent watching TV on IVDD.

Figure S23: Scatter plot for the effect of time spent watching TV on LBP.

Figure S24: Scatter plot for the effect of time spent watching TV on sciatica.

Figure S25: Funnel plot for the effect of time spent driving on CS.

Figure S26: Funnel plot for the effect of time spent driving on IVDD.

Figure S27: Funnel plot for the effect of time spent driving on LBP.

Figure S28: Funnel plot for the effect of time spent driving on sciatica.

Figure S29: Funnel plot for the effect of time spent using computer on CS.

Figure S30: Funnel plot for the effect of time spent using computer on IVDD.

Figure S31: Funnel plot for the effect of time spent using computer on LBP.

Figure S32: Funnel plot for the effect of time spent using computer on sciatica.

Figure S33: Funnel plot for the effect of time spent watching TV on CS.

Figure S34: Funnel plot for the effect of time spent watching TV on IVDD.

Figure S35: Funnel plot for the effect of time spent watching TV on LBP.

Figure S36: Funnel plot for the effect of time spent watching TV on sciatica.

Figure S37: Forest plot for the effect of time spent driving on CS.

Figure S38: Forest plot for the effect of time spent driving on IVDD.

Figure S39: Forest plot for the effect of time spent driving on LBP.

Figure S40: Forest plot for the effect of time spent driving on sciatica.

Figure S41: Forest plot for the effect of time spent using computer on CS.

Figure S42: Forest plot for the effect of time spent using computer on IVDD.

Figure S43: Forest plot for the effect of time spent using computer on LBP.

Figure S44: Forest plot for the effect of time spent using computer on sciatica.

Figure S45: Forest plot for the effect of time spent watching TV on CS.

Figure S46: Forest plot for the effect of time spent watching TV on IVDD.

Figure S47: Forest plot for the effect of time spent watching TV on LBP.

Figure S48: Forest plot for the effect of time spent watching TV on sciatica.


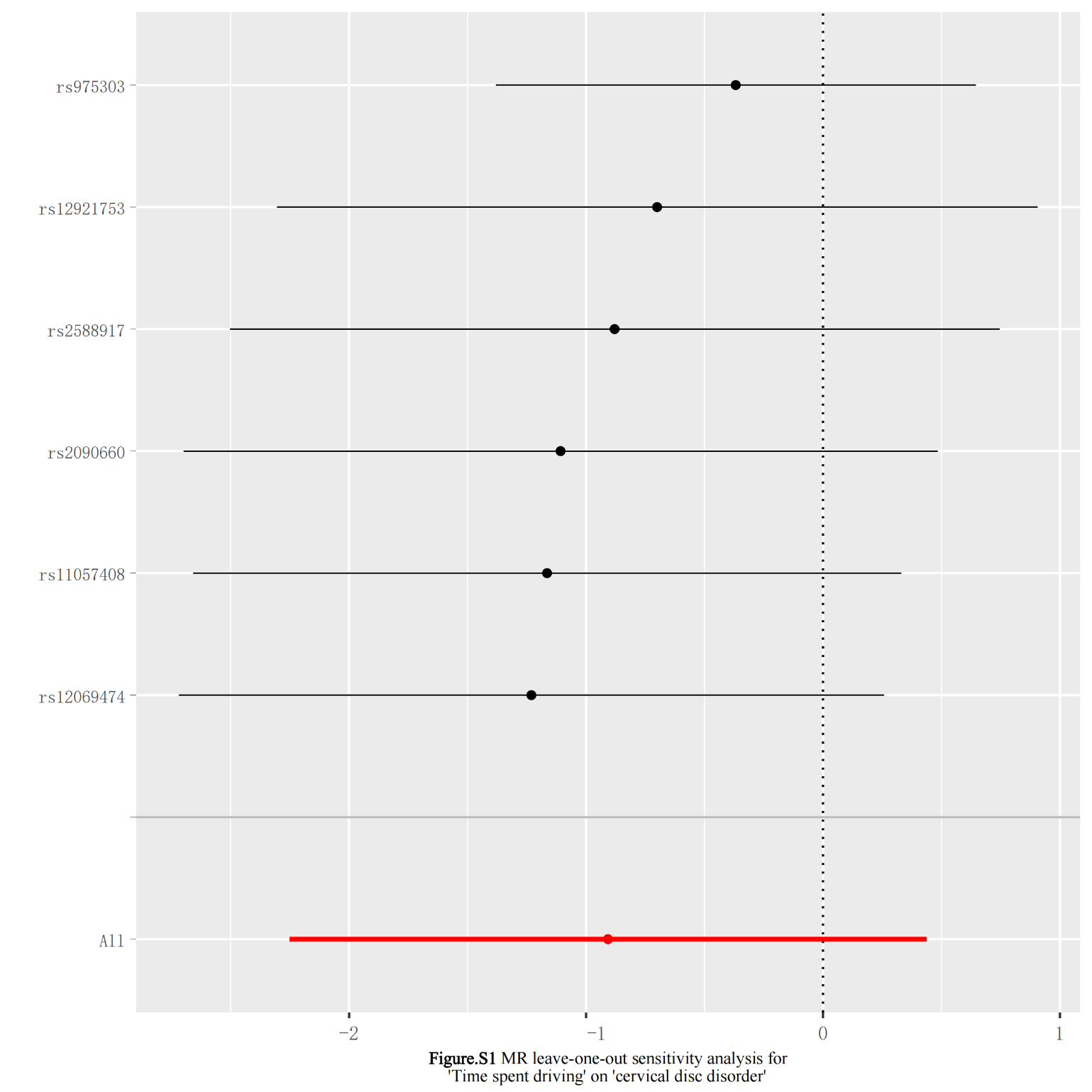

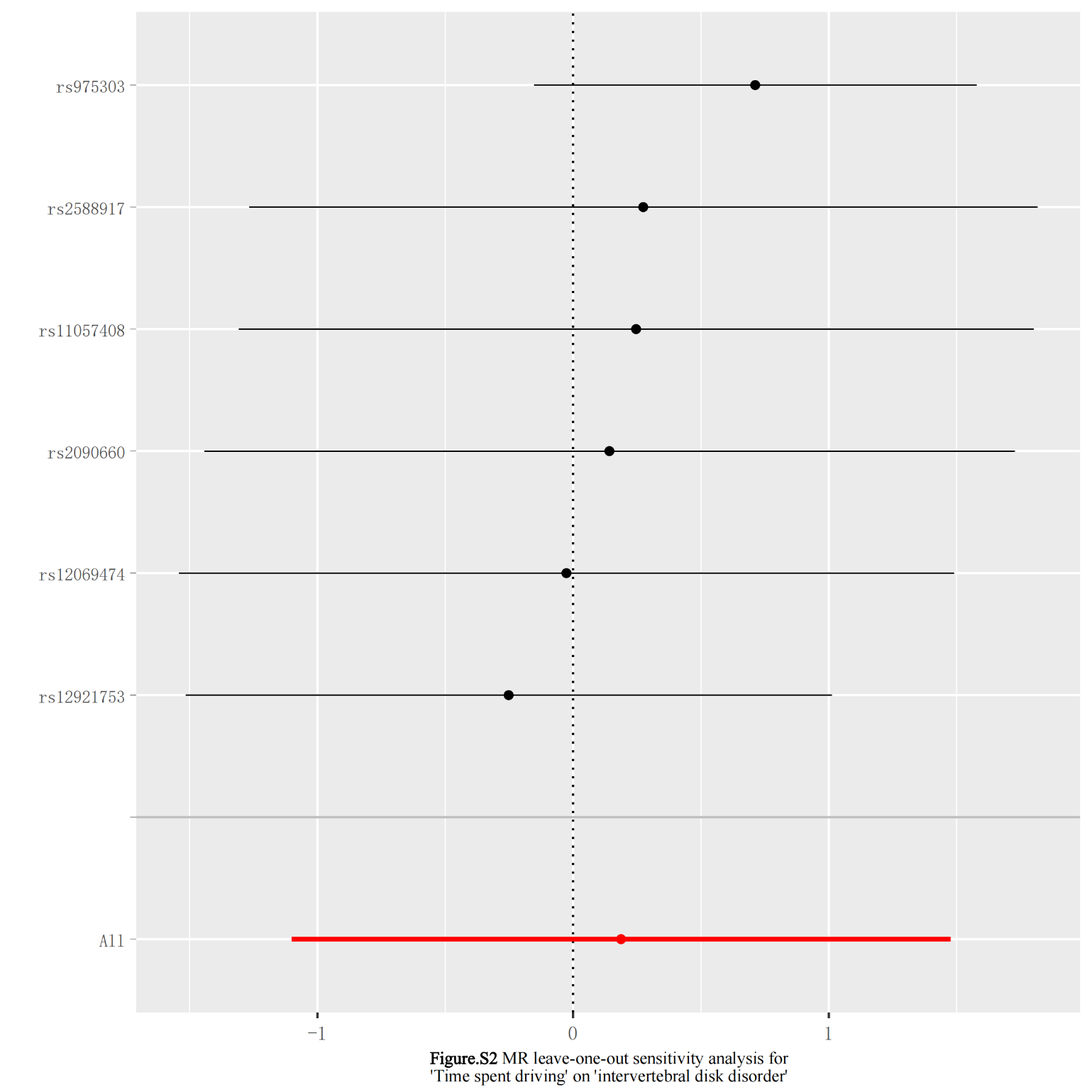

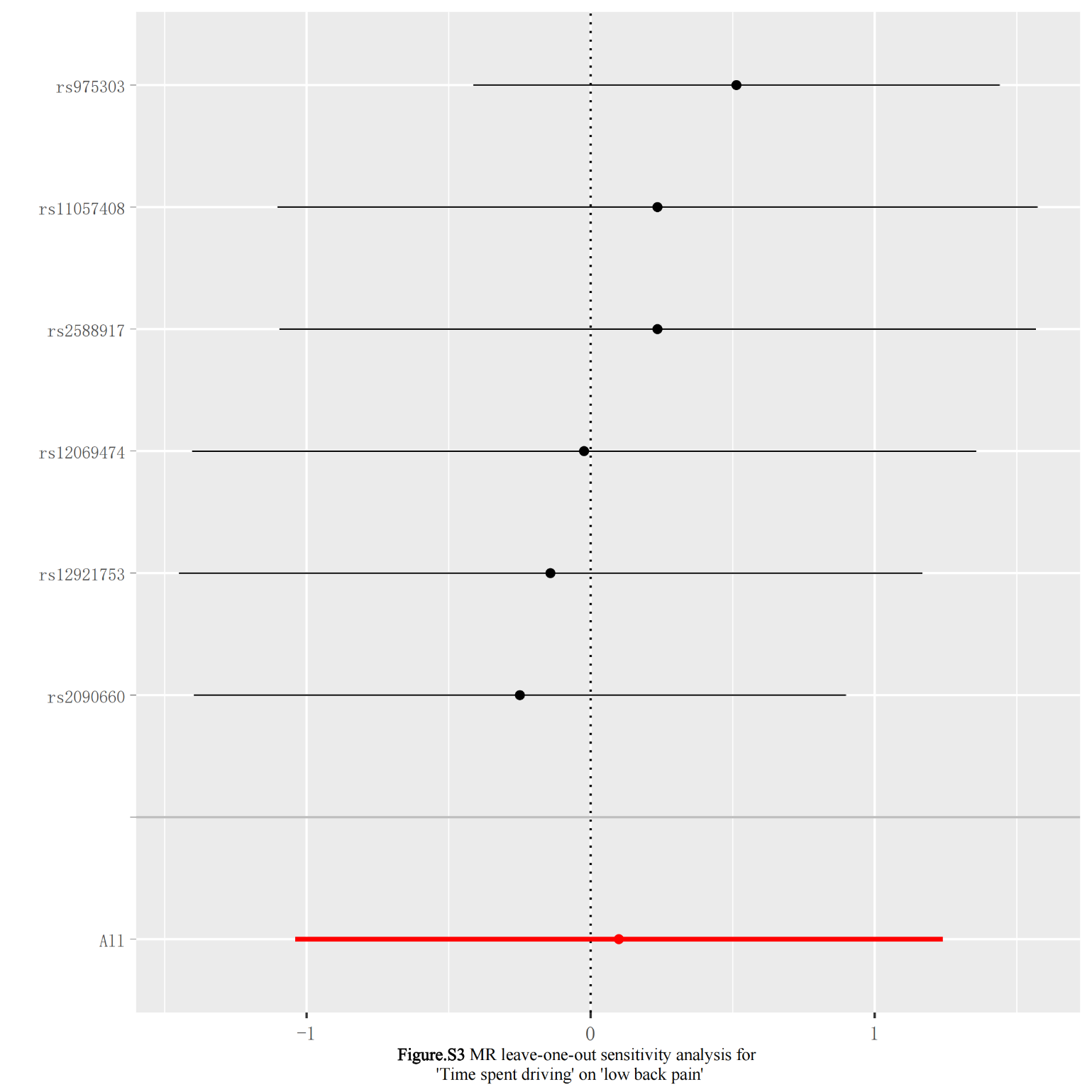

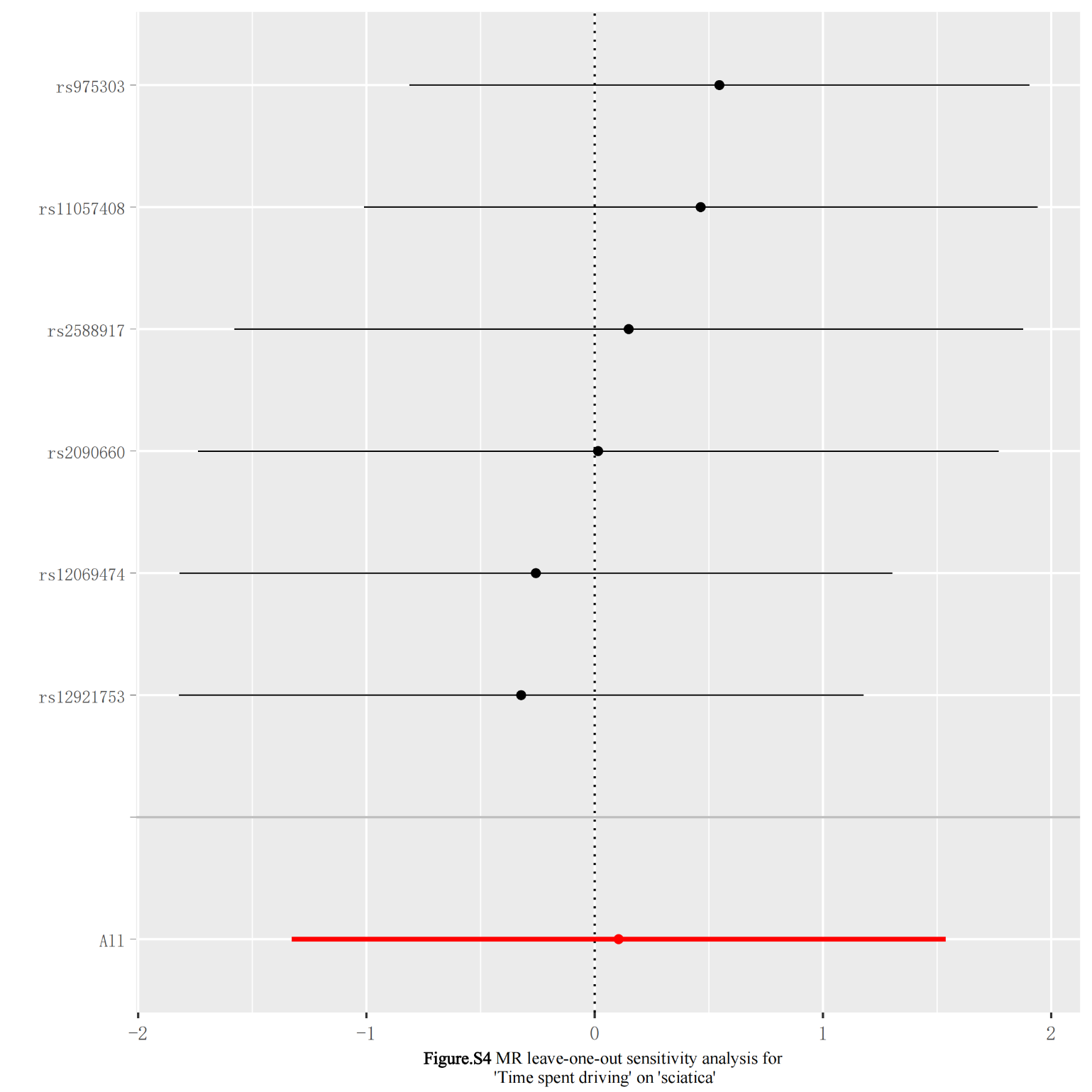

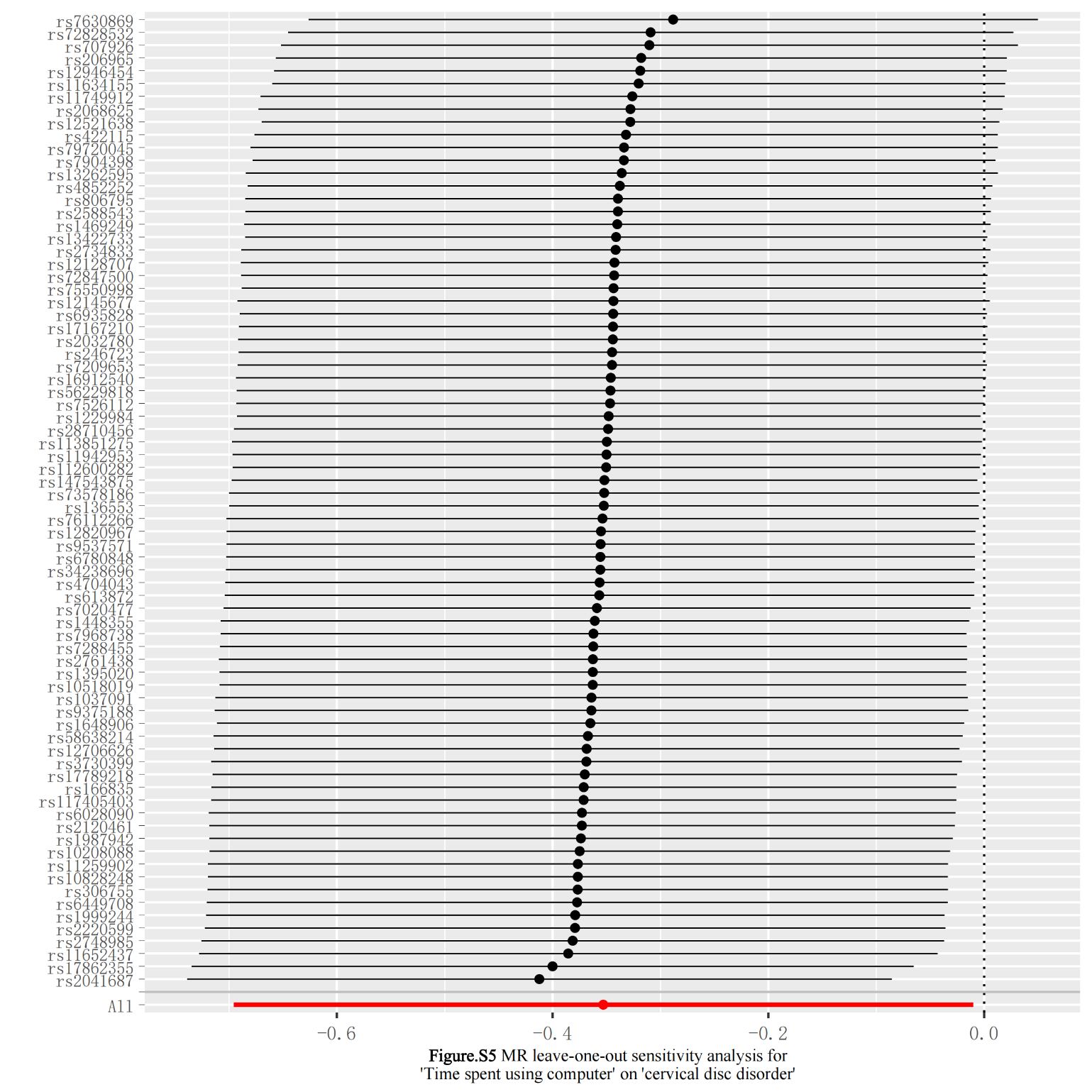

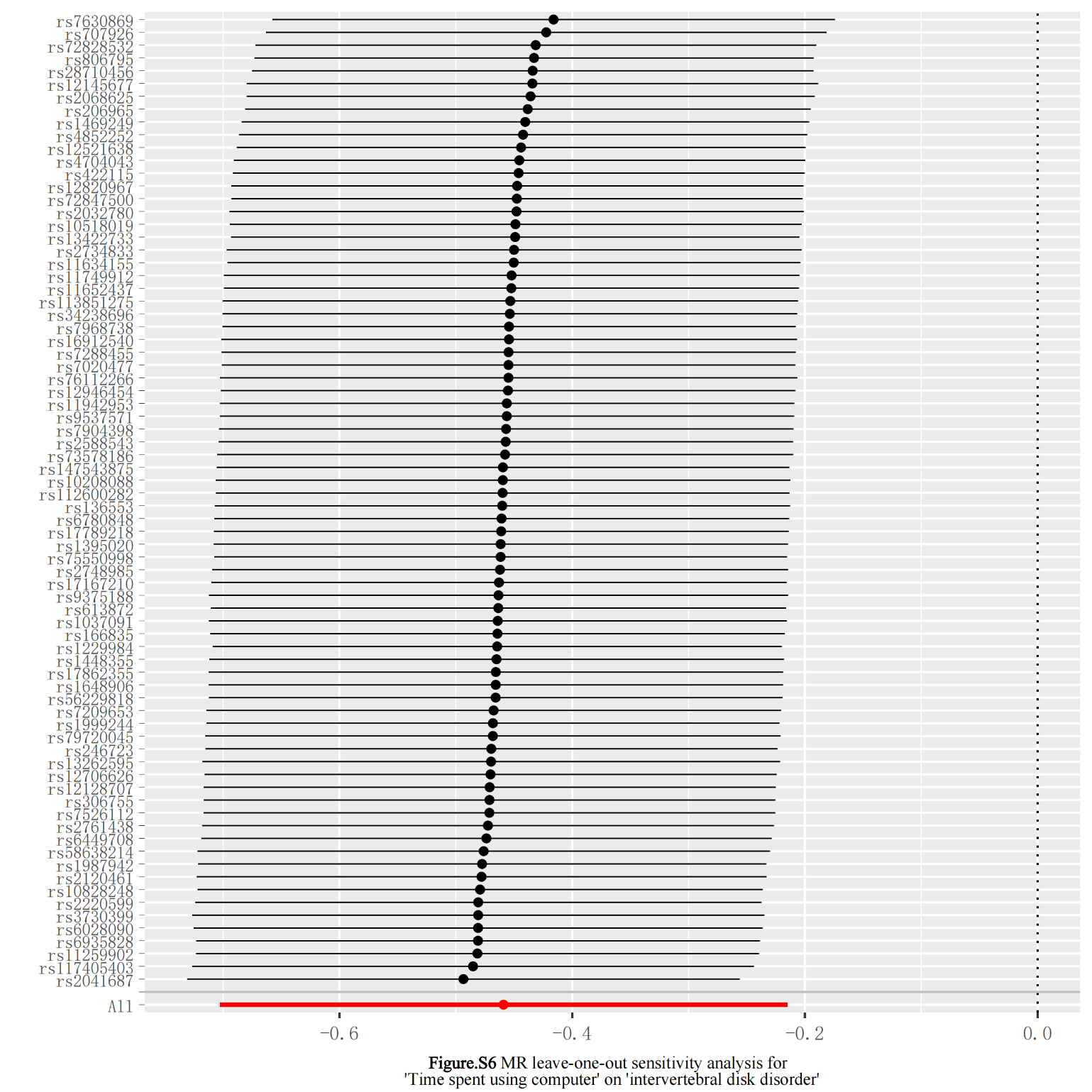

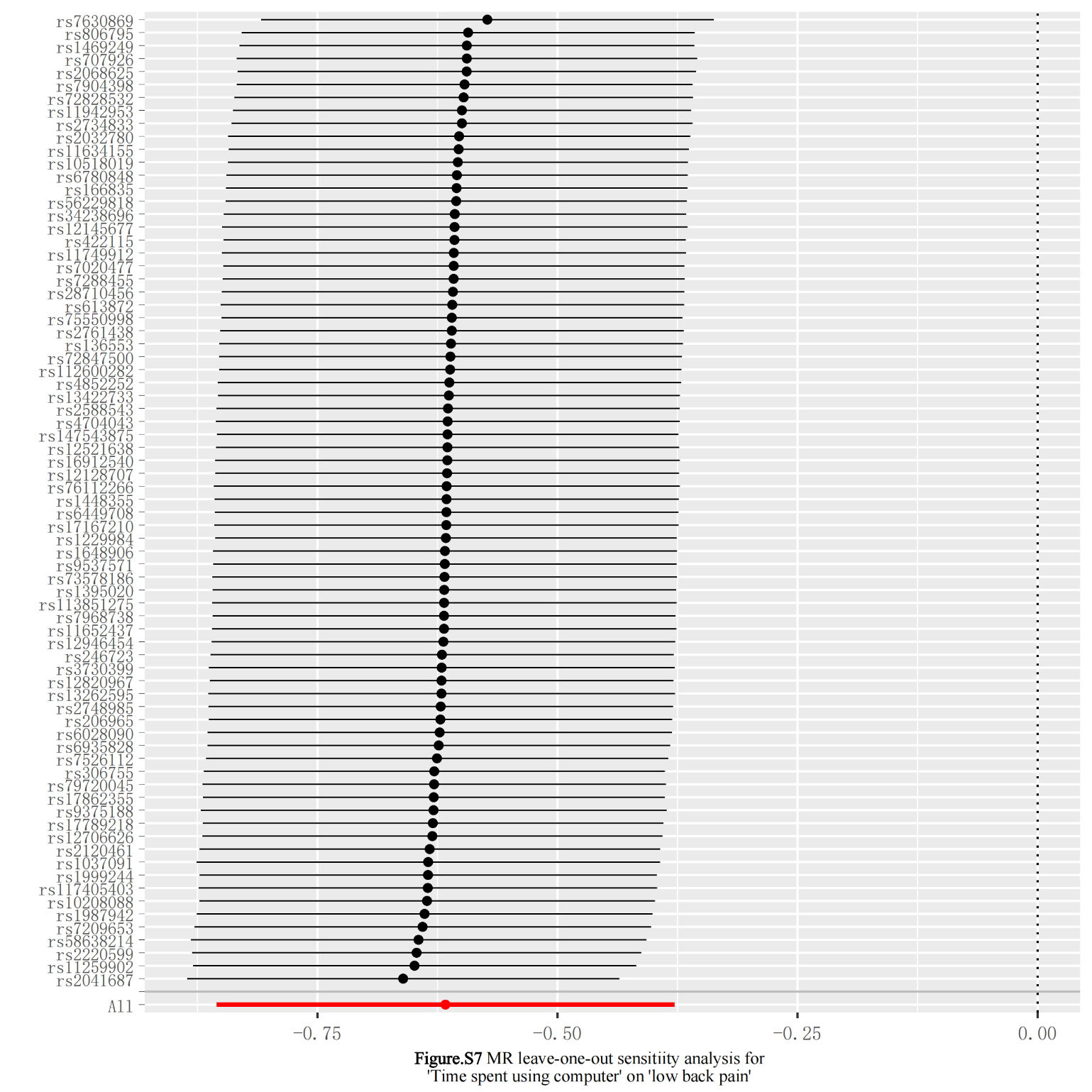

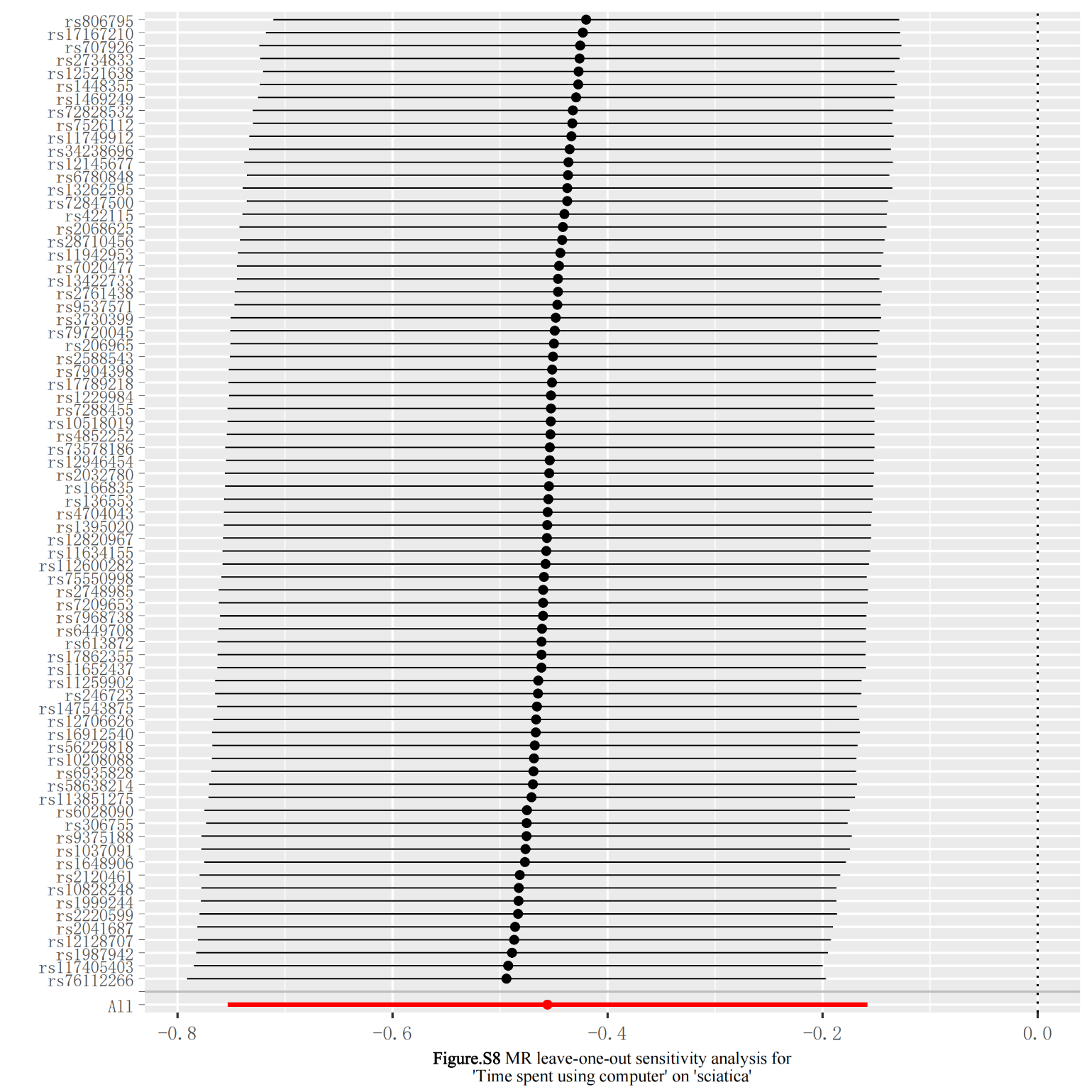

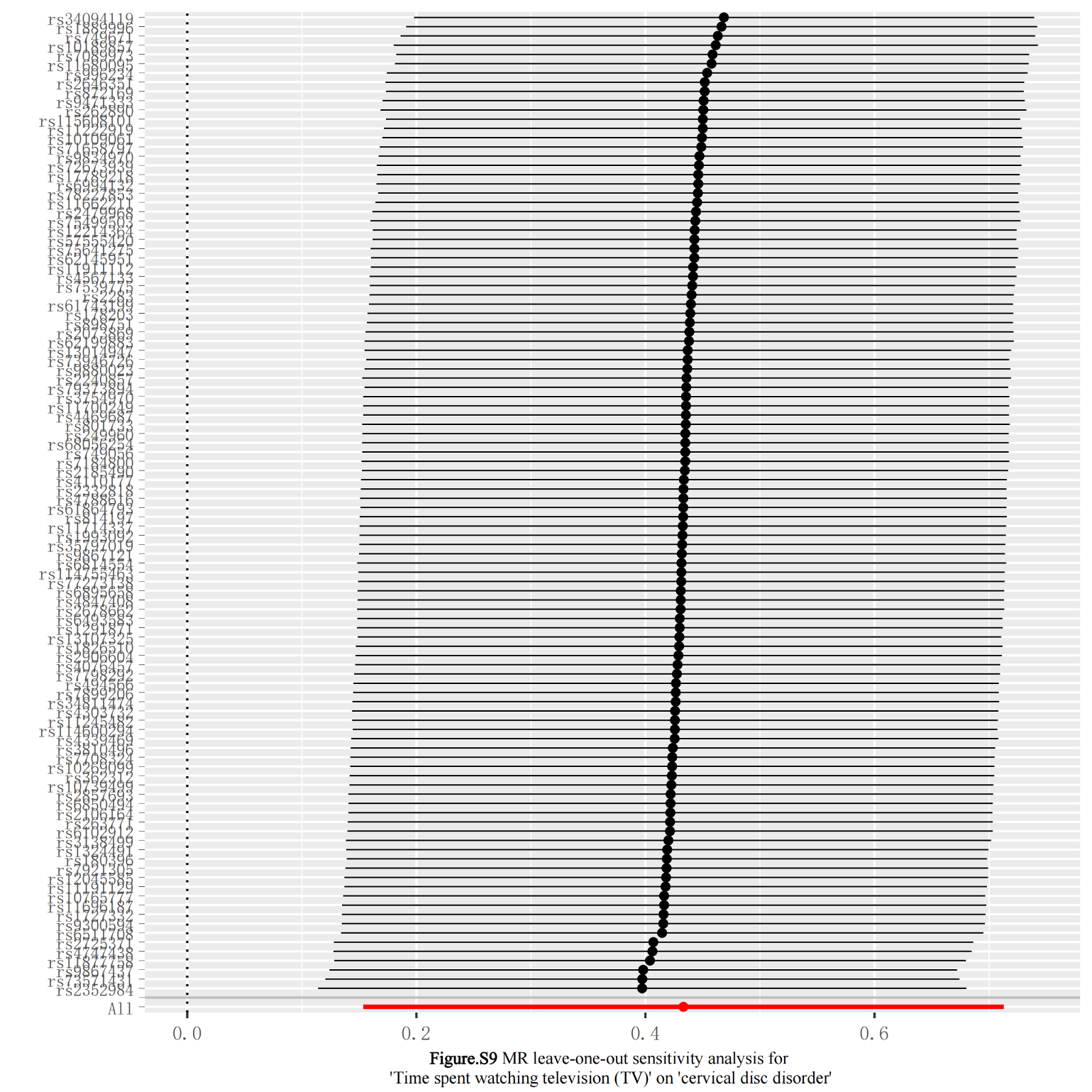

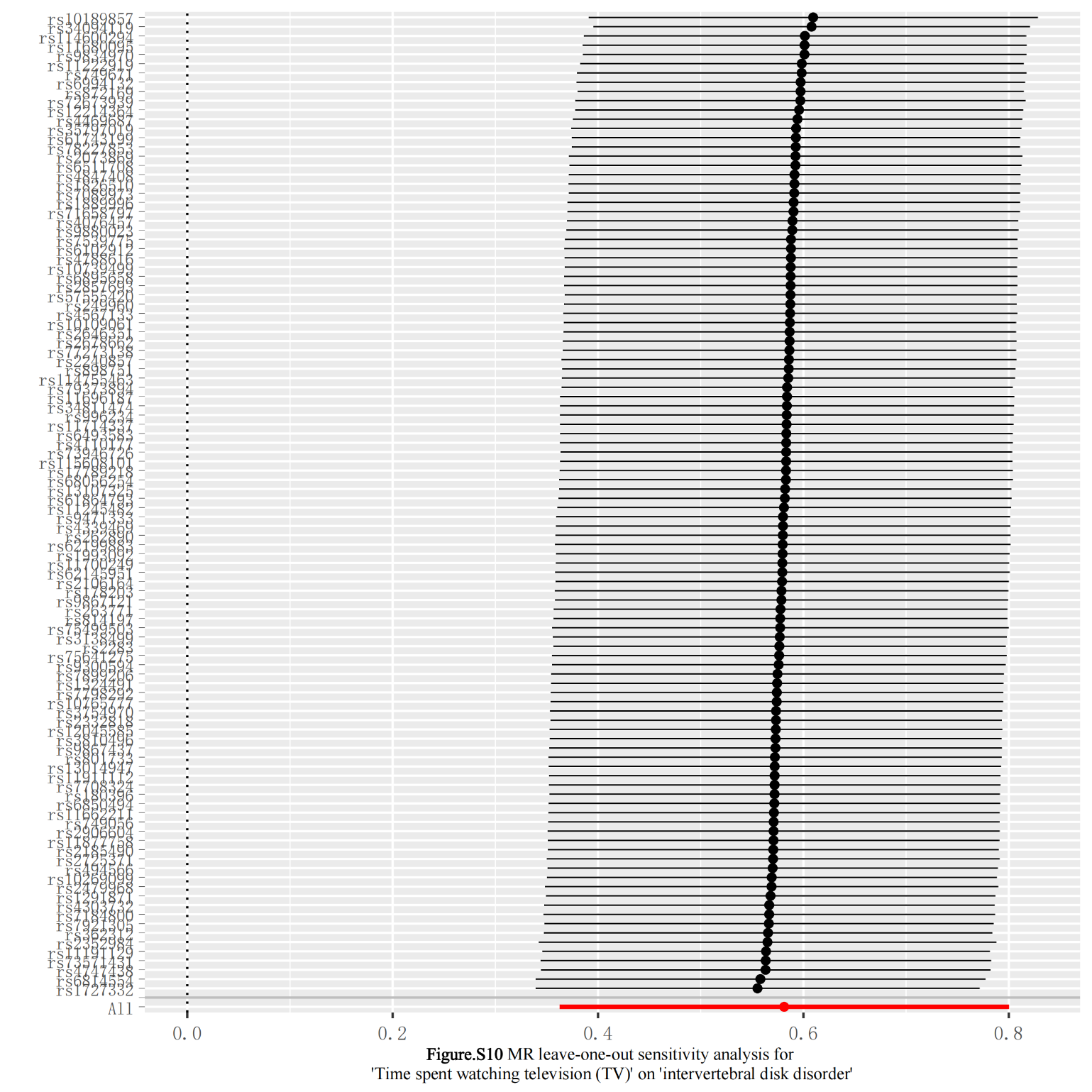

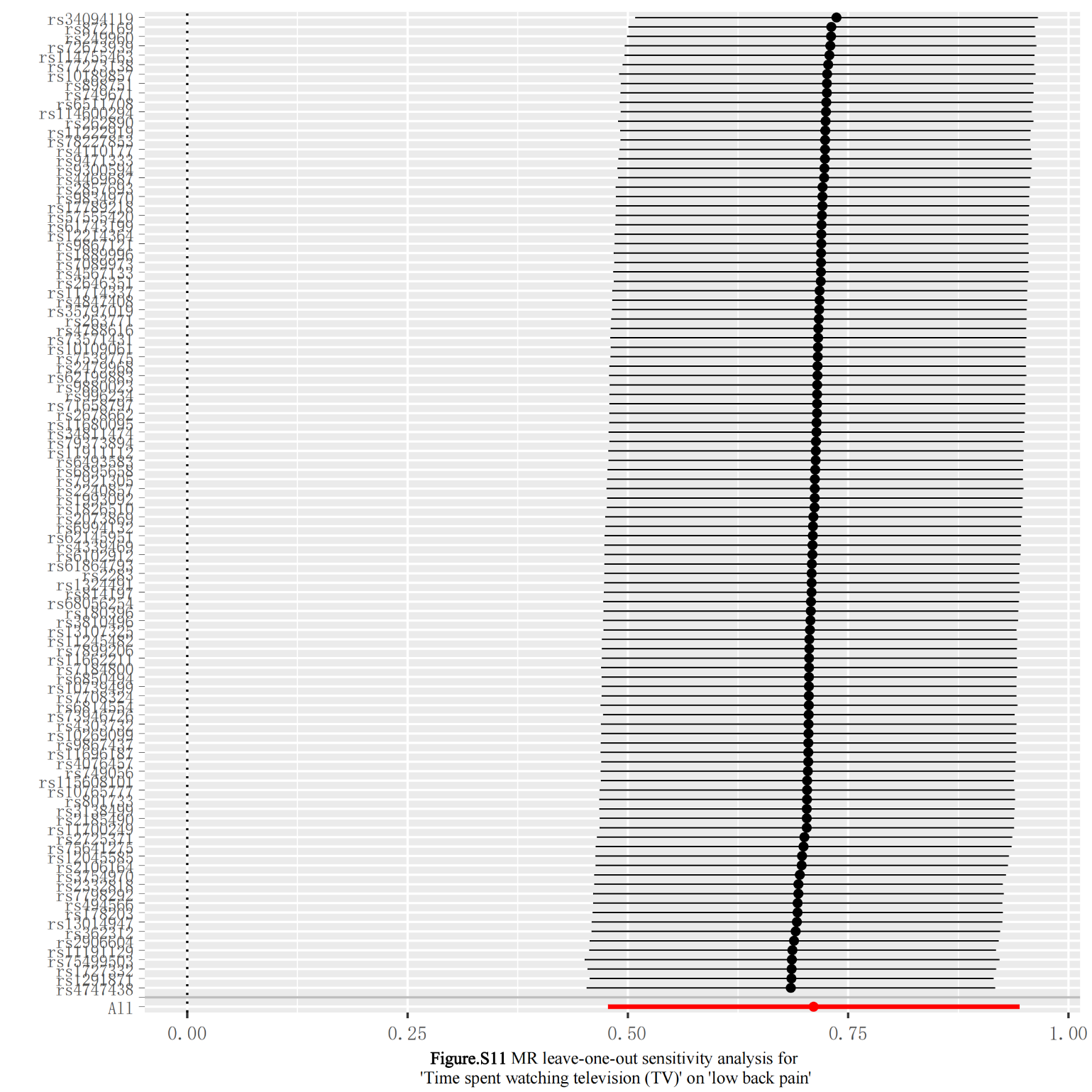

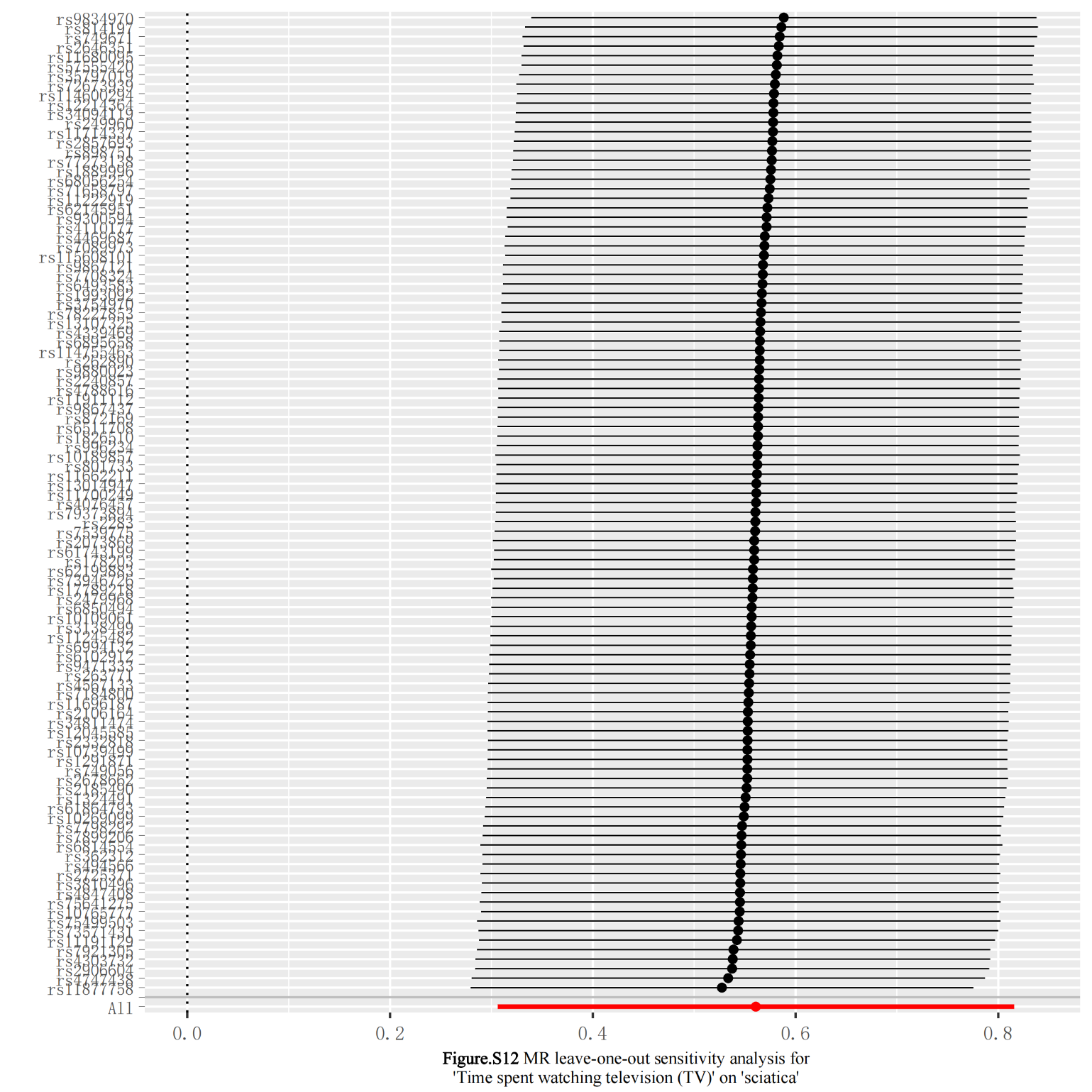

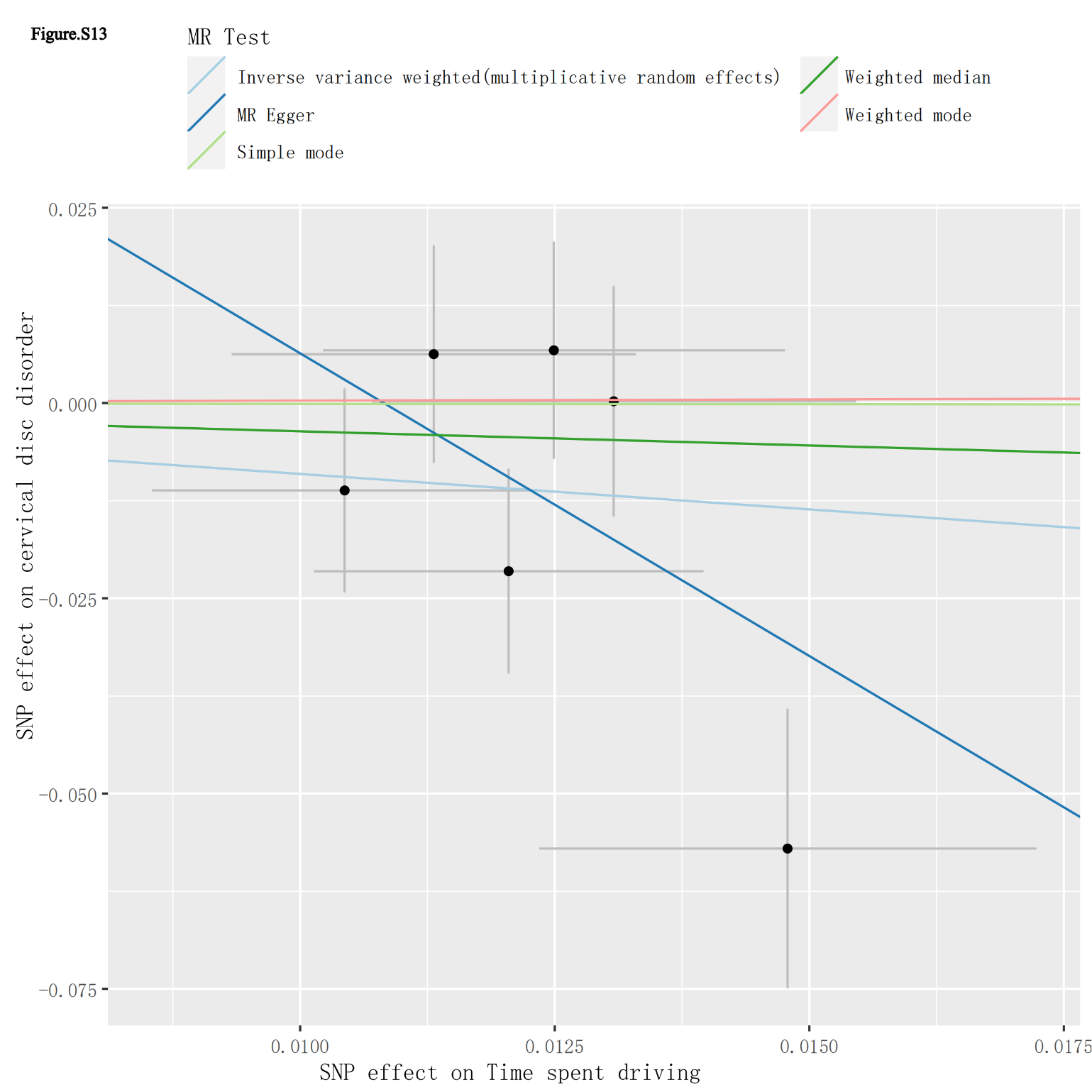

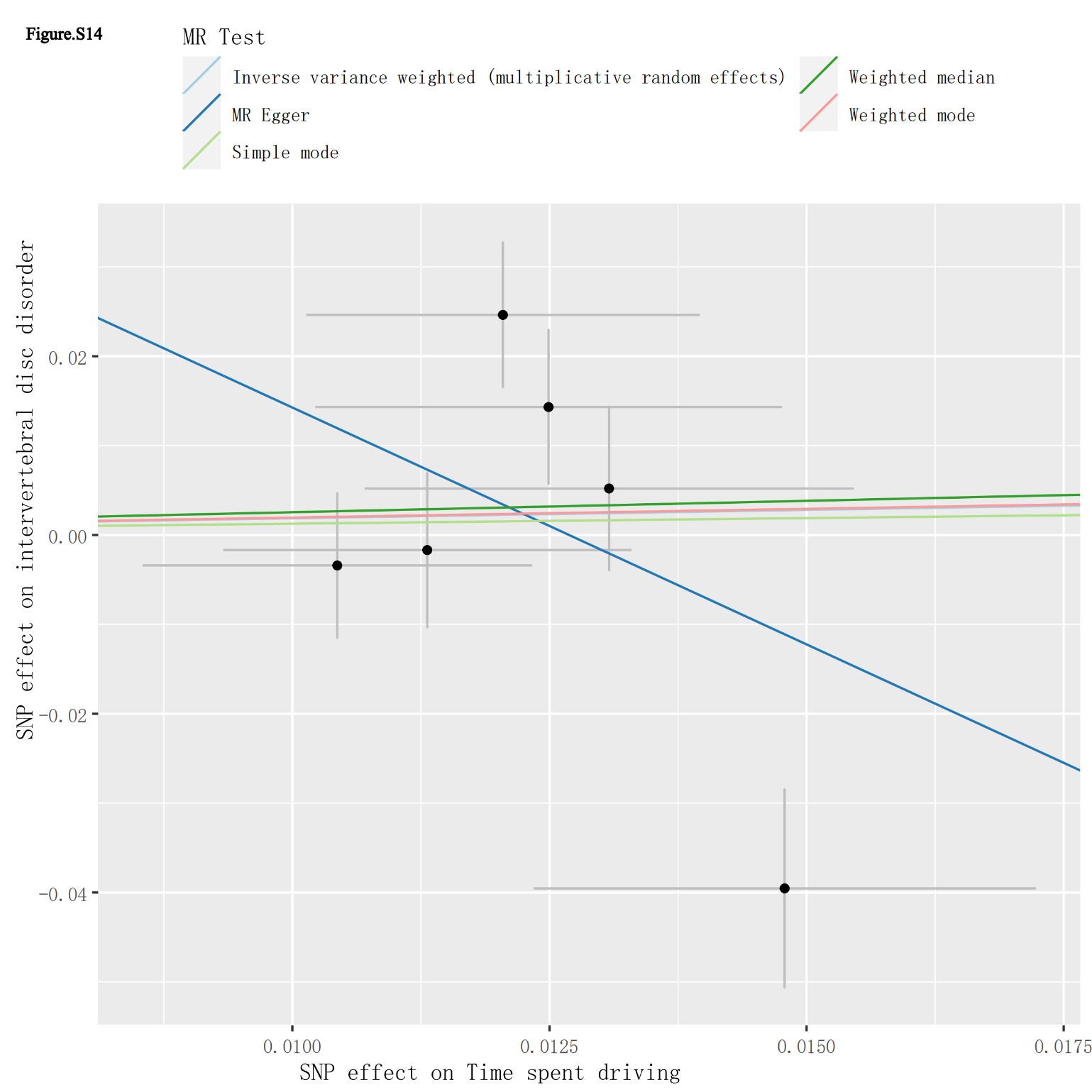

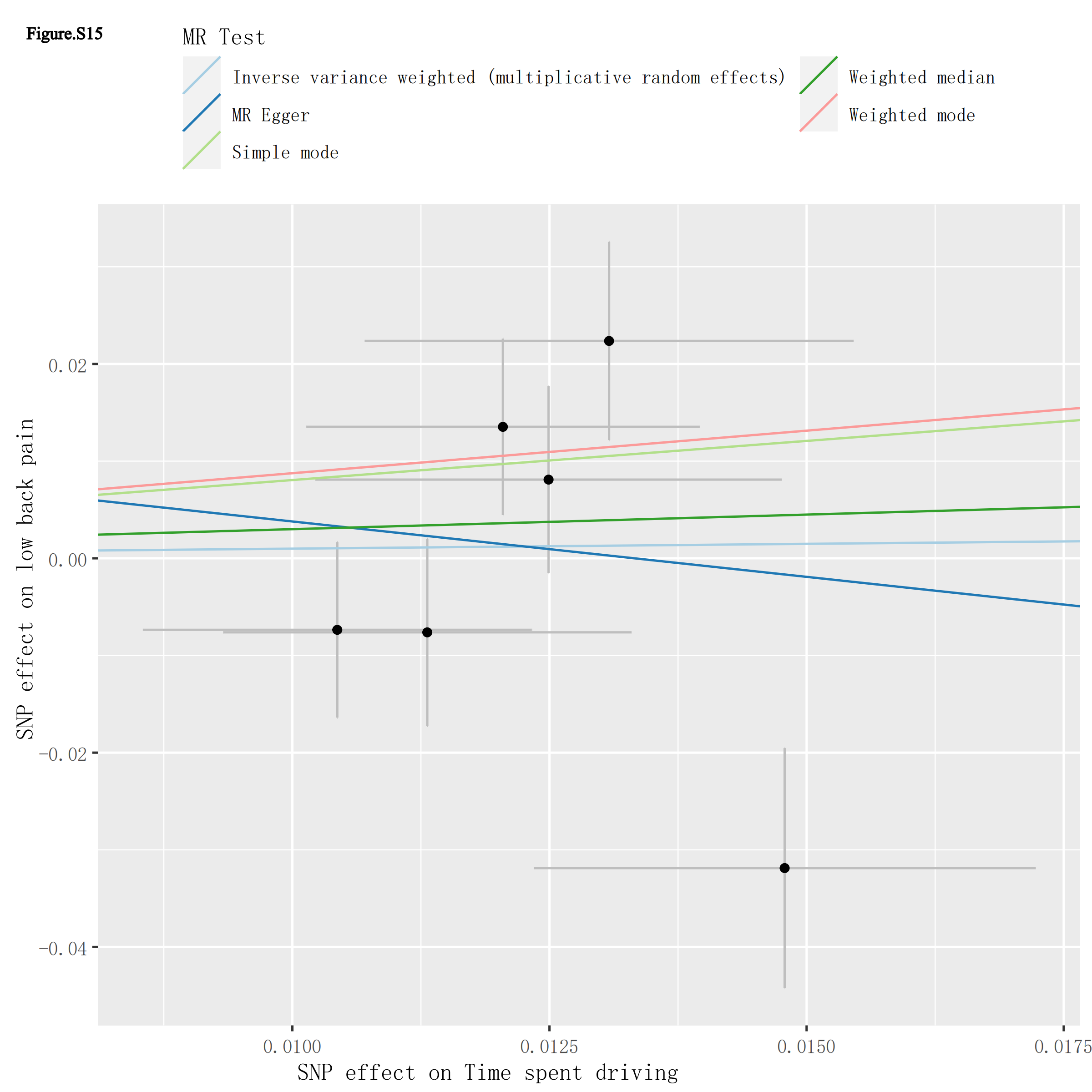

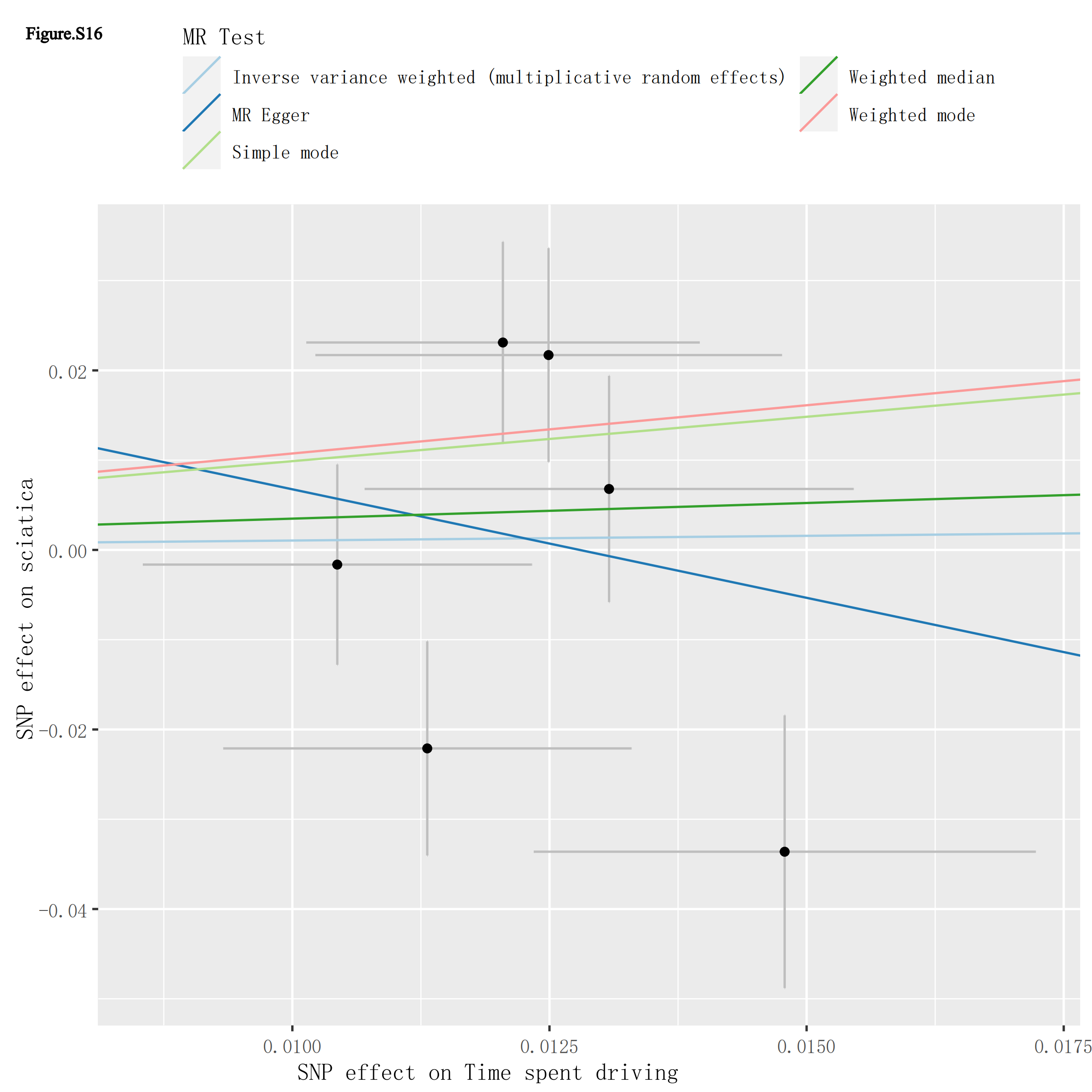

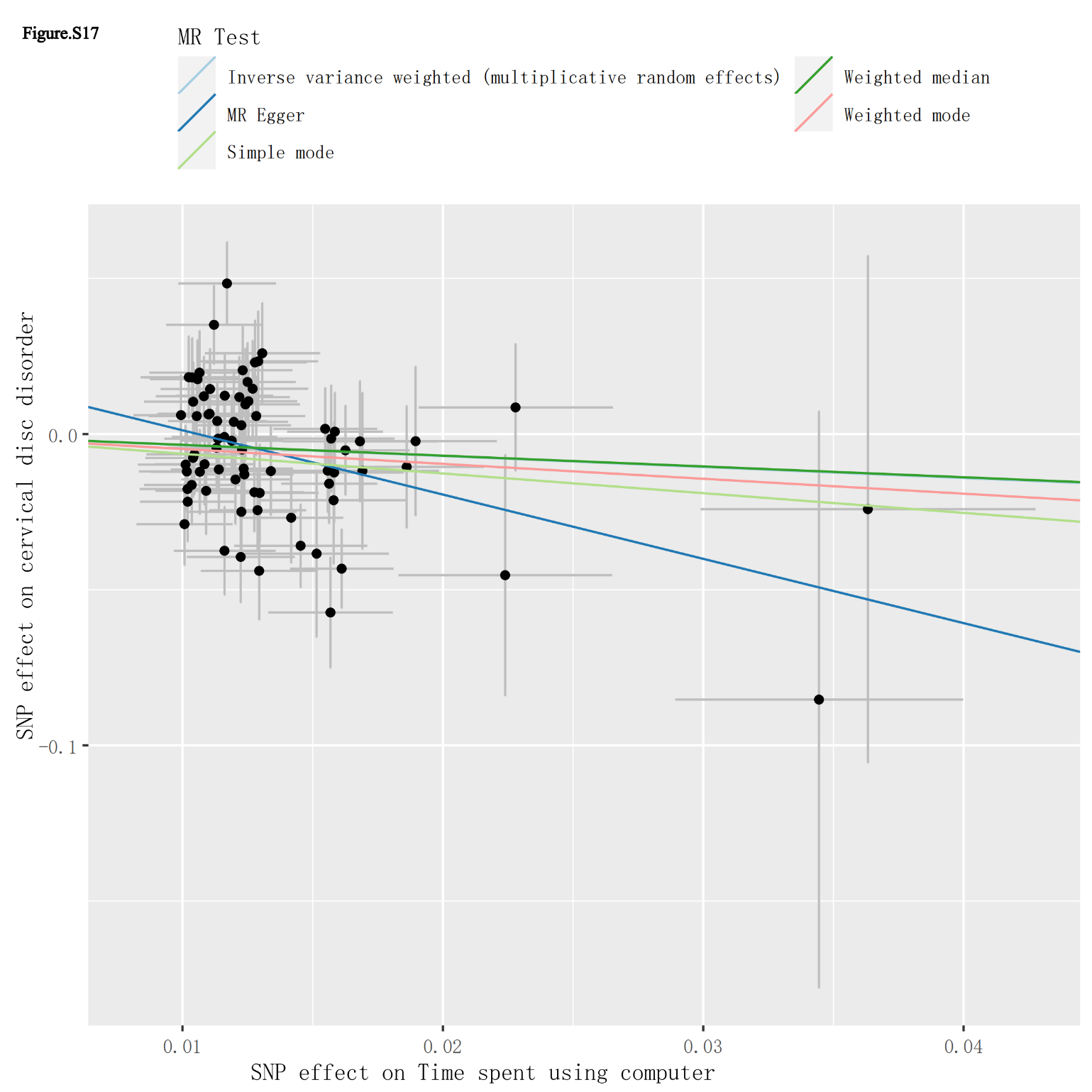

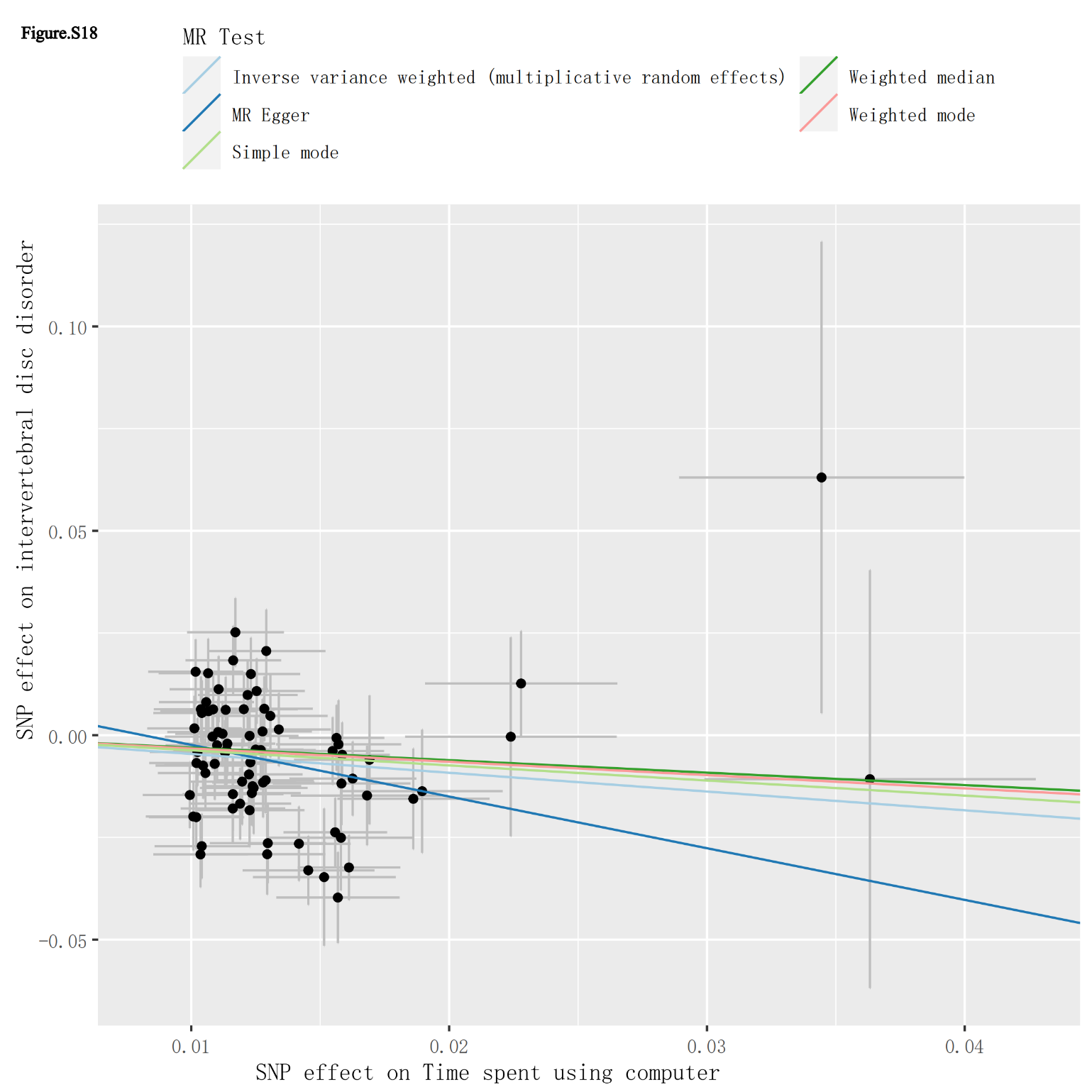

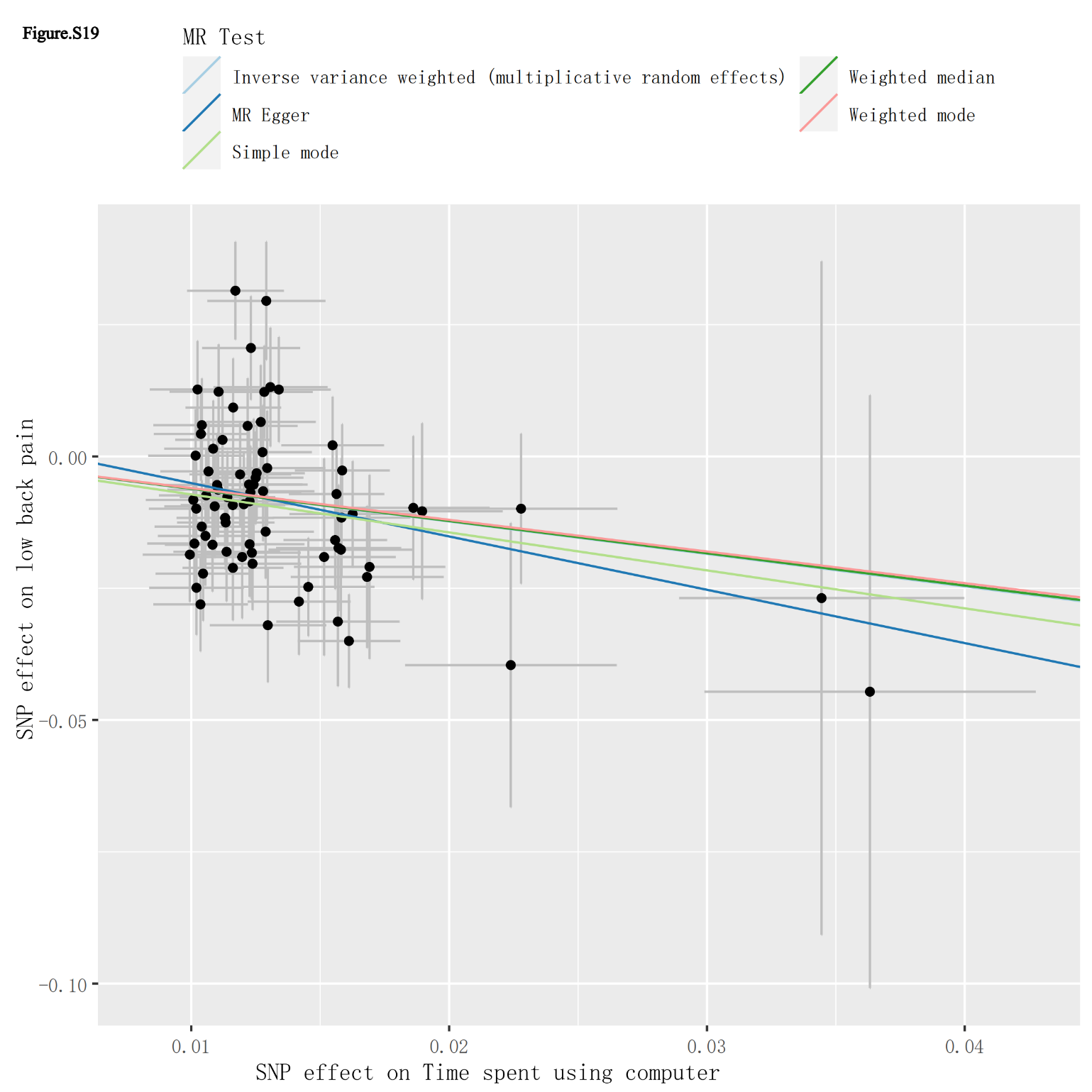

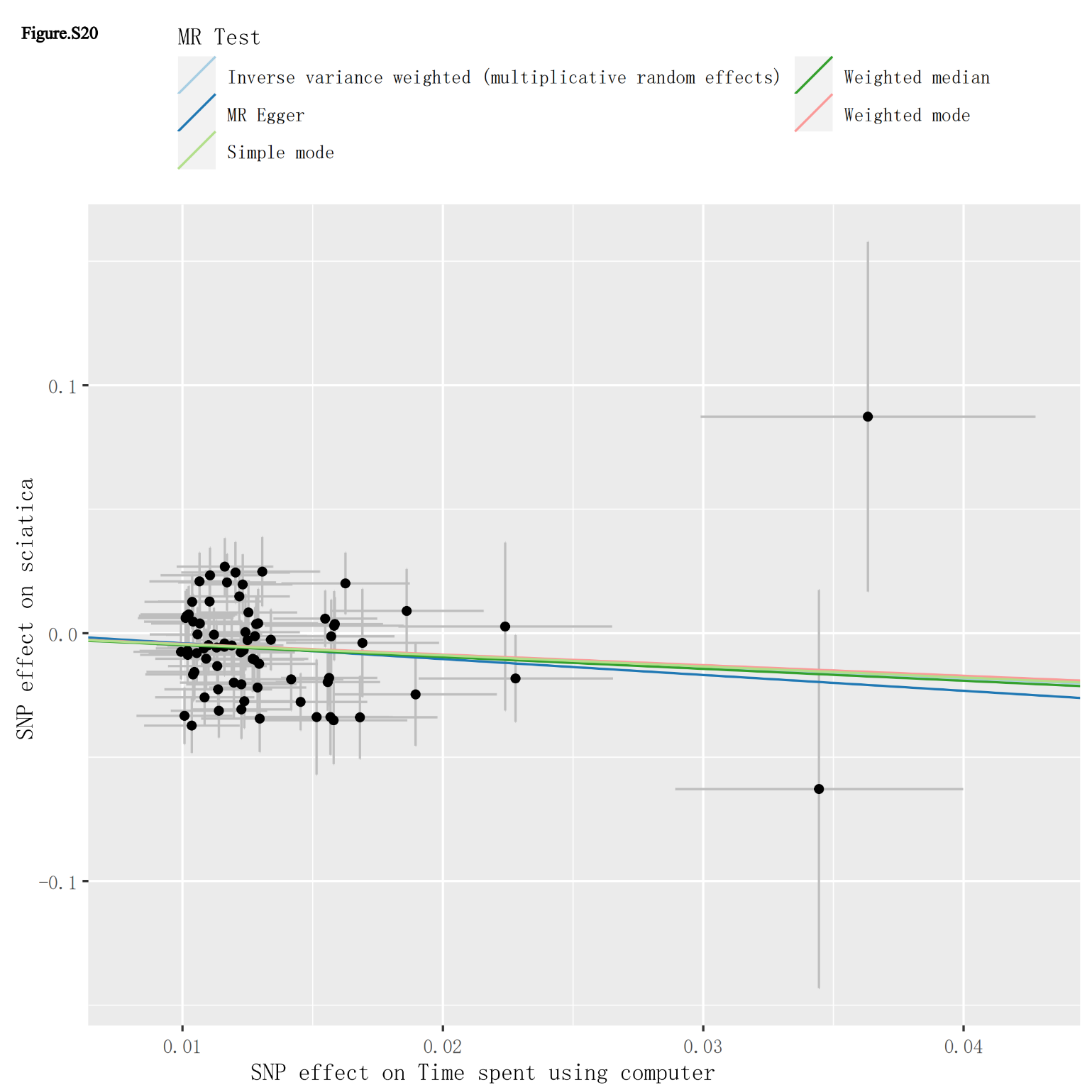

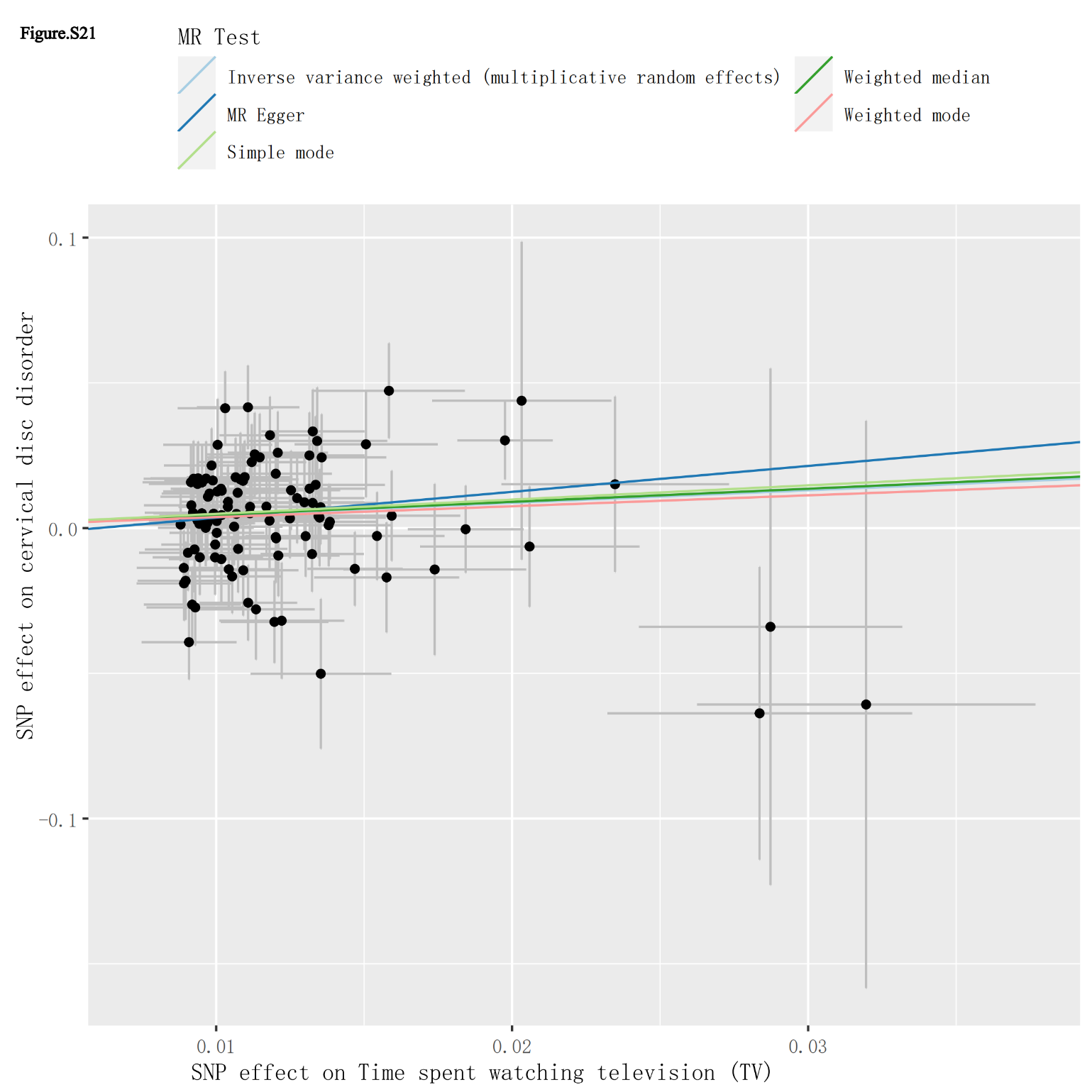

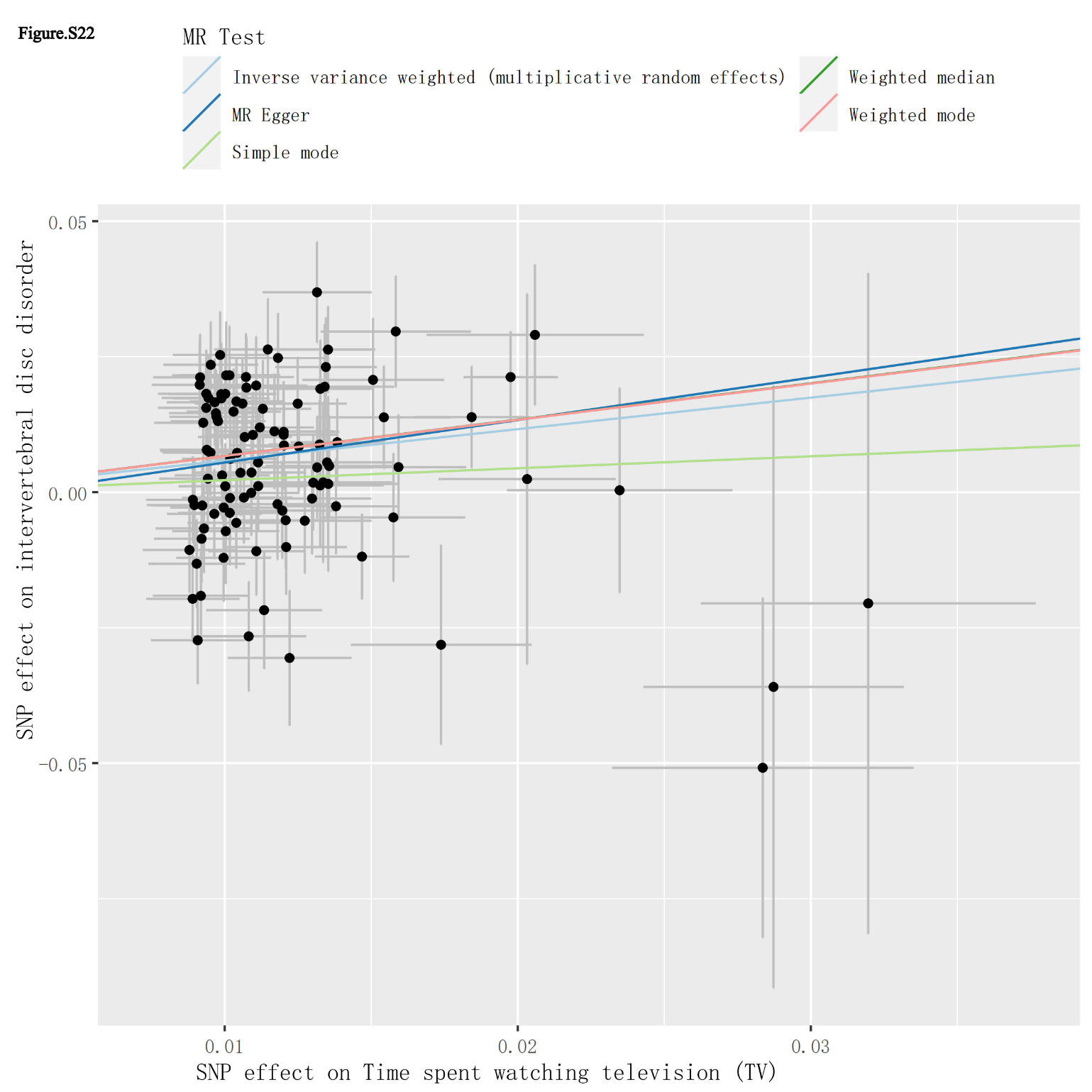

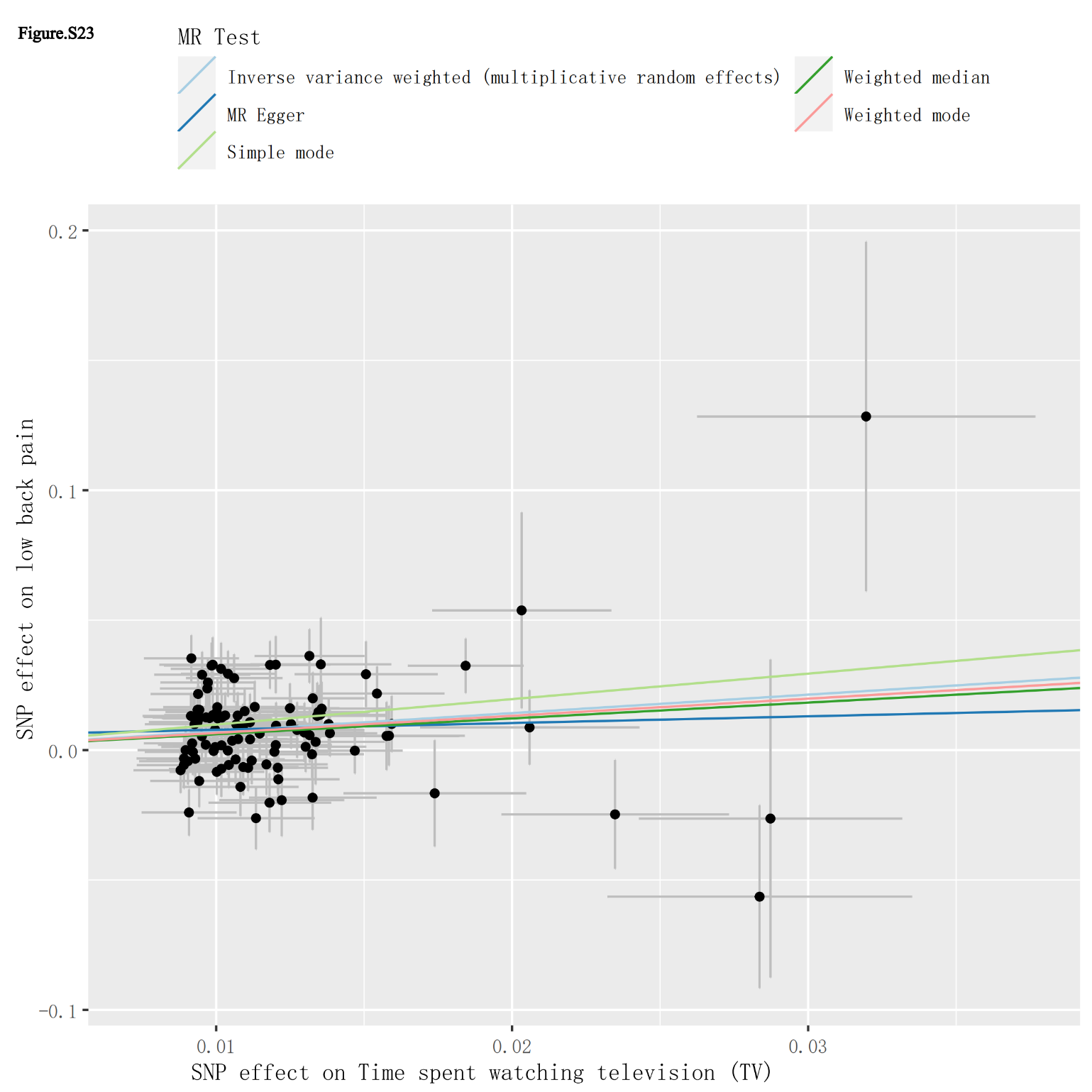

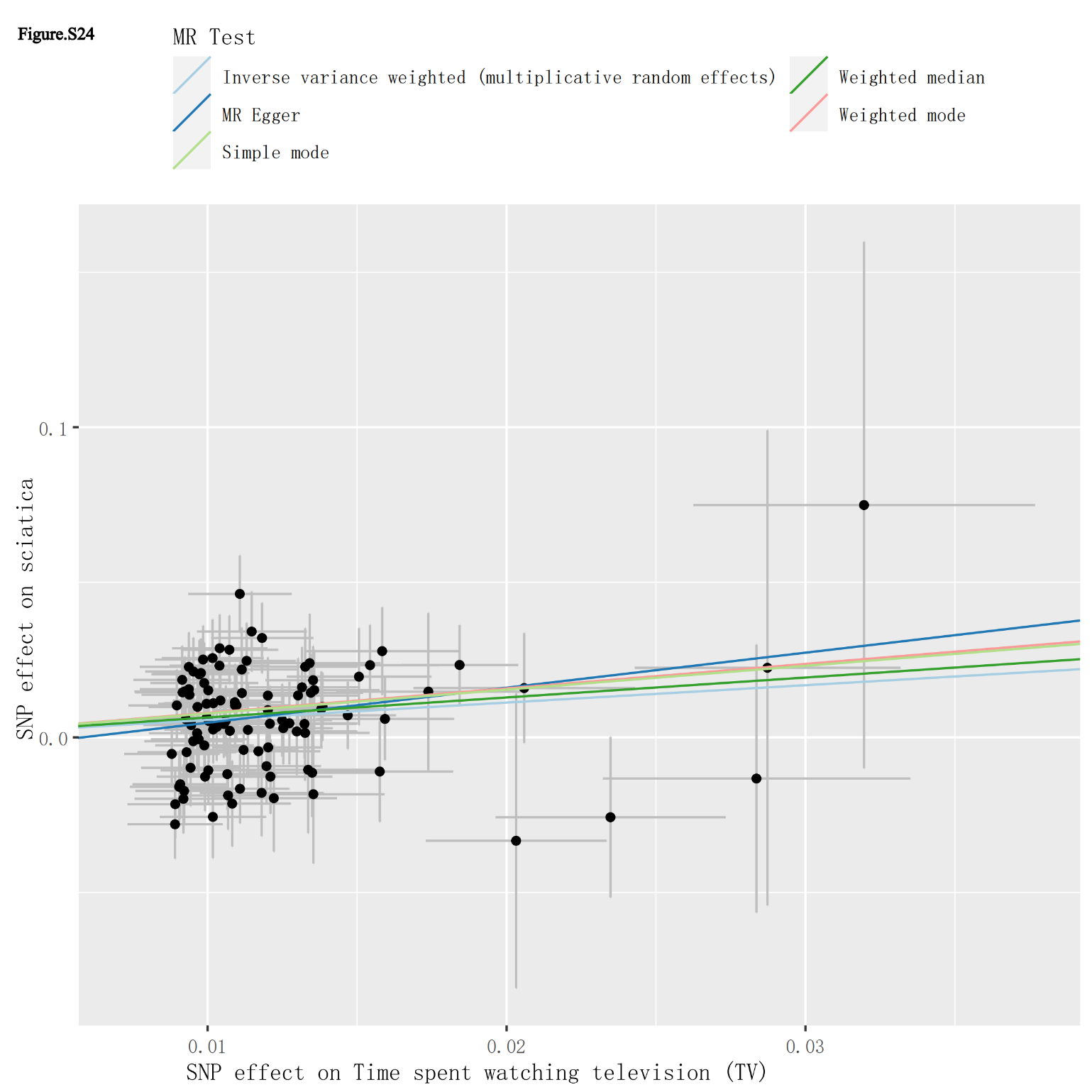

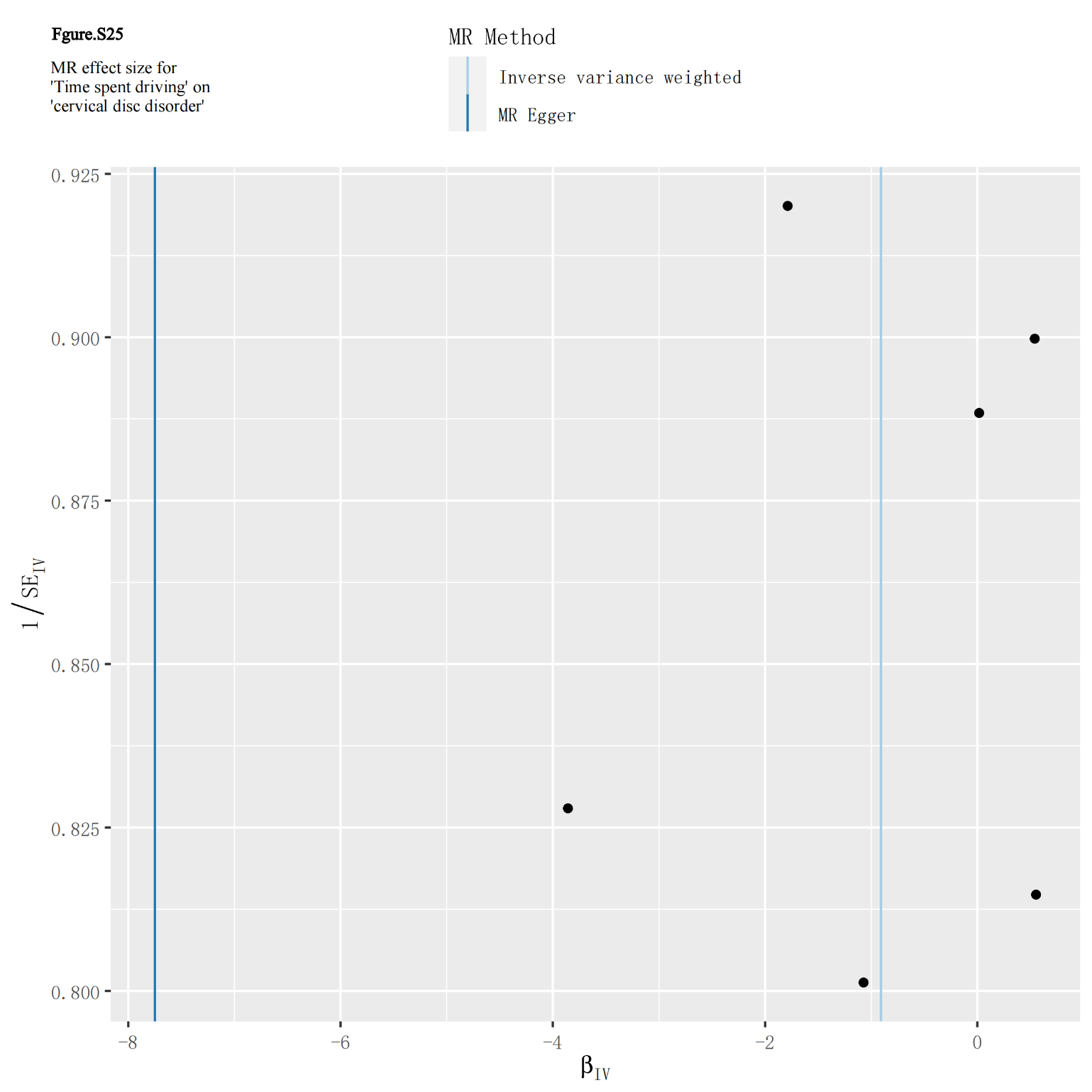

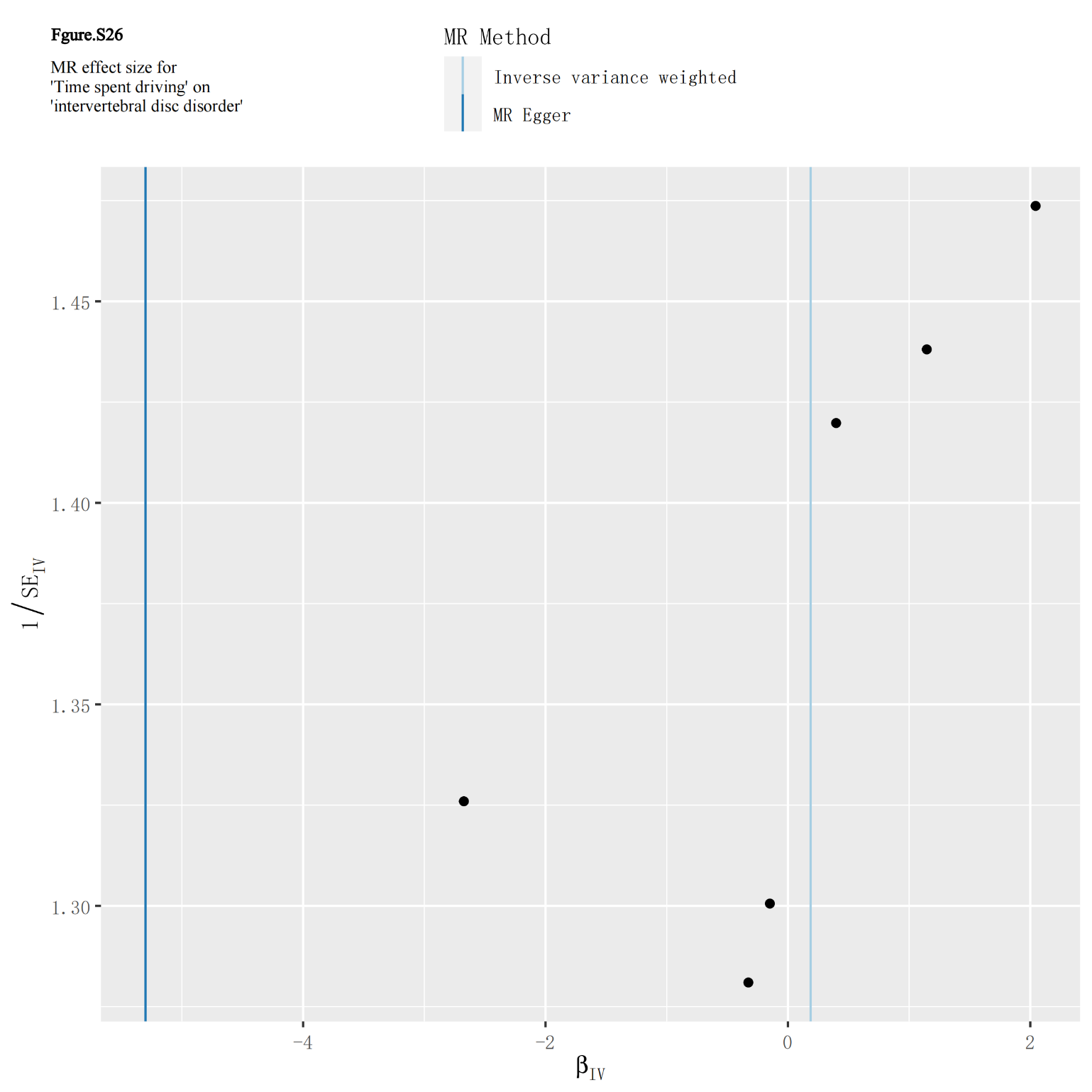

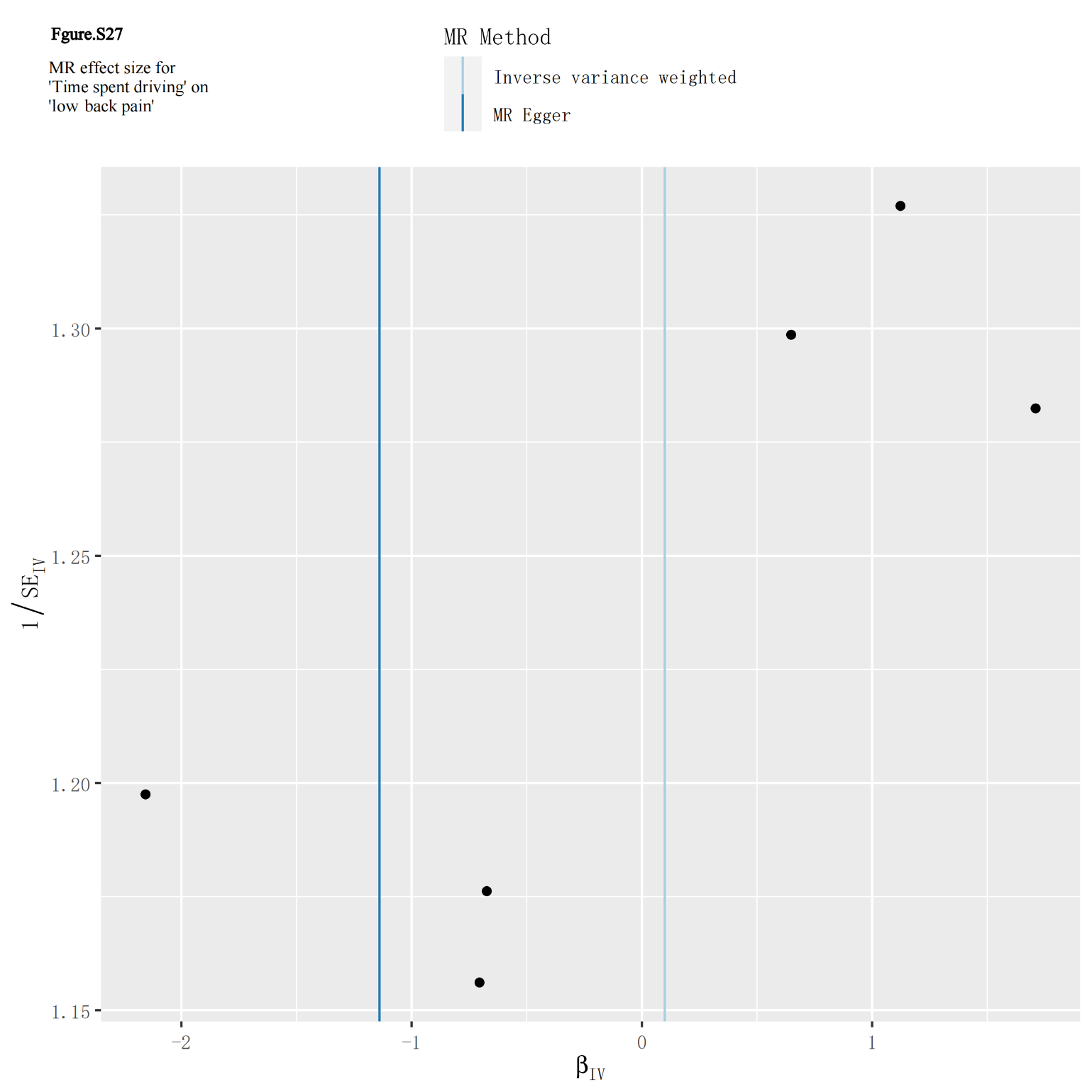

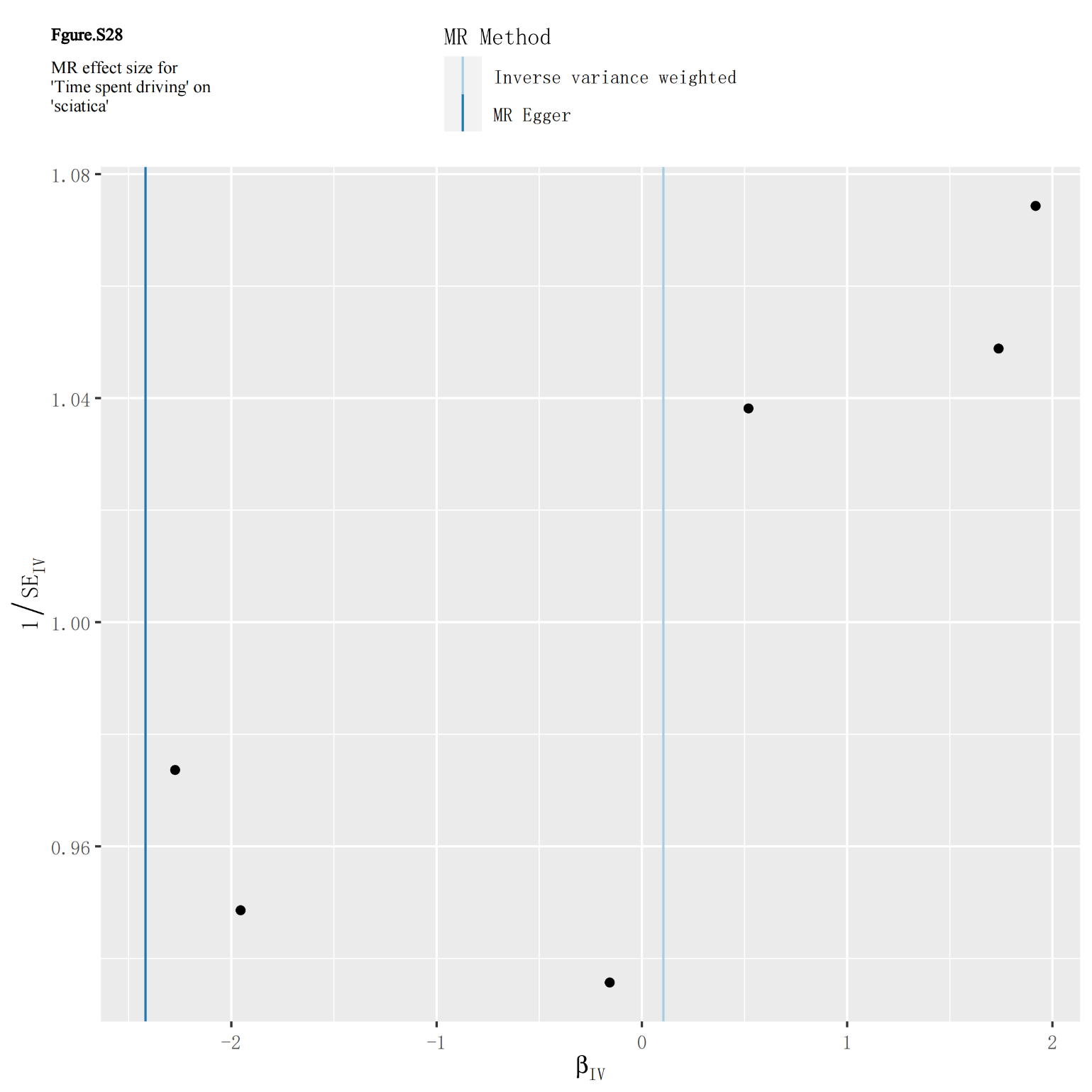

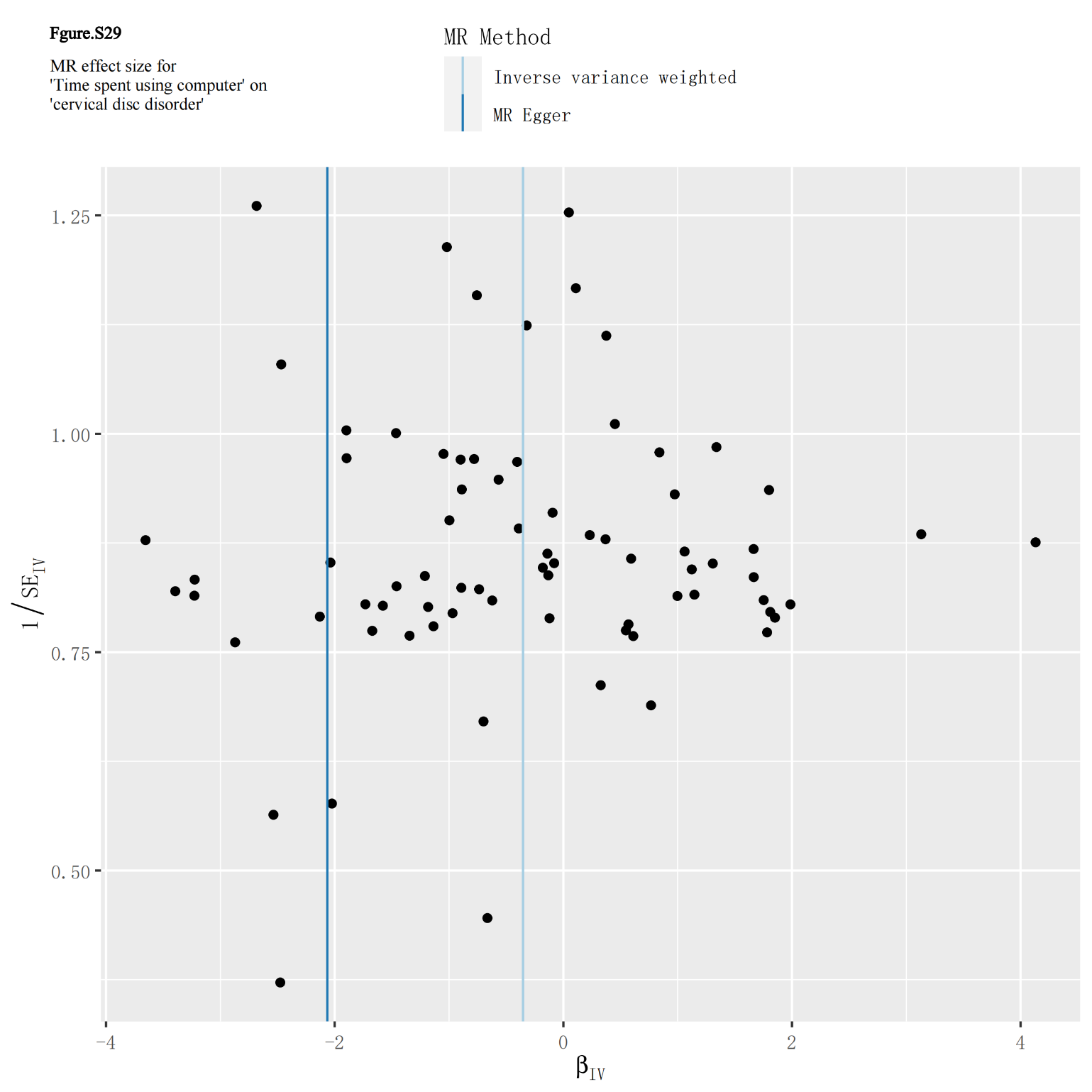

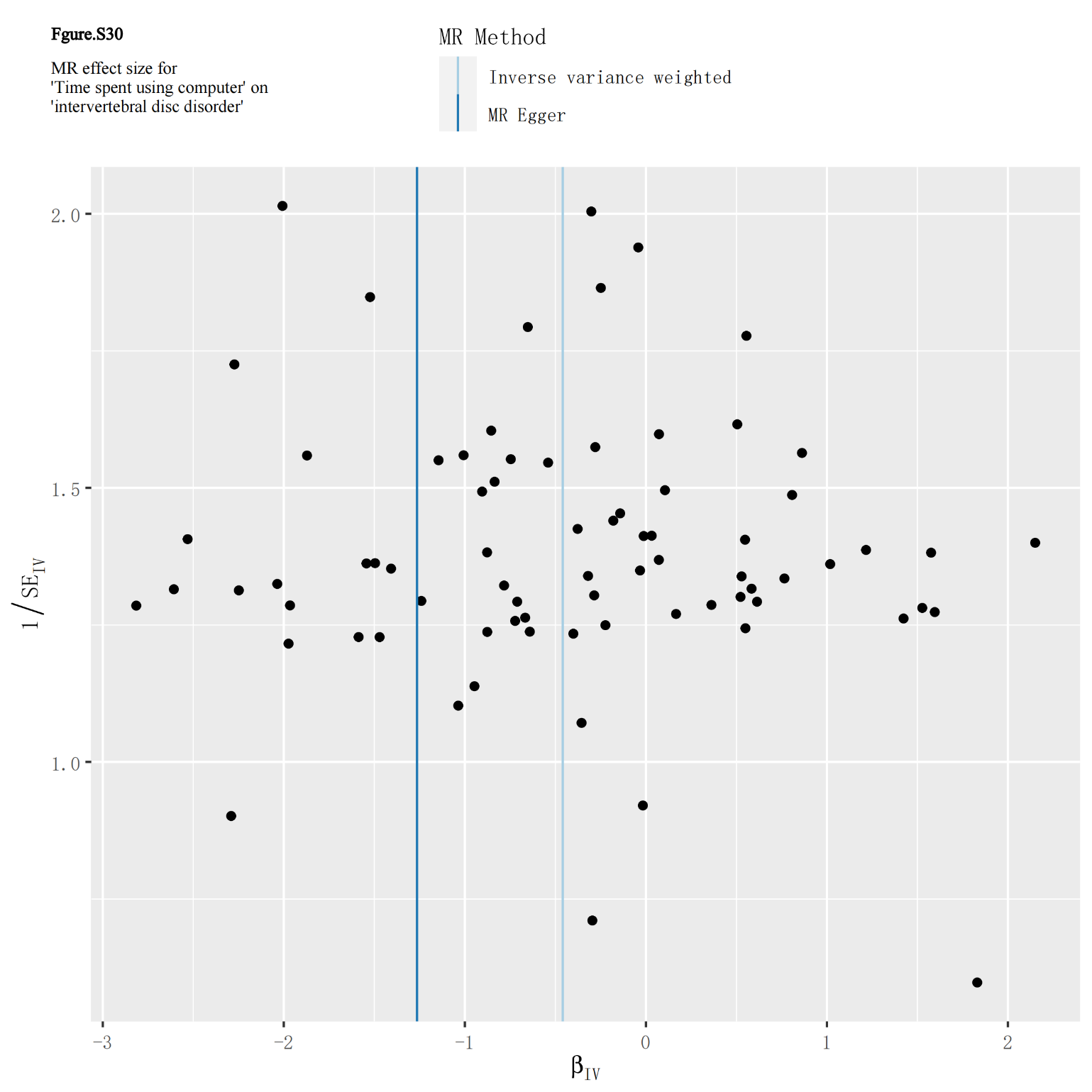

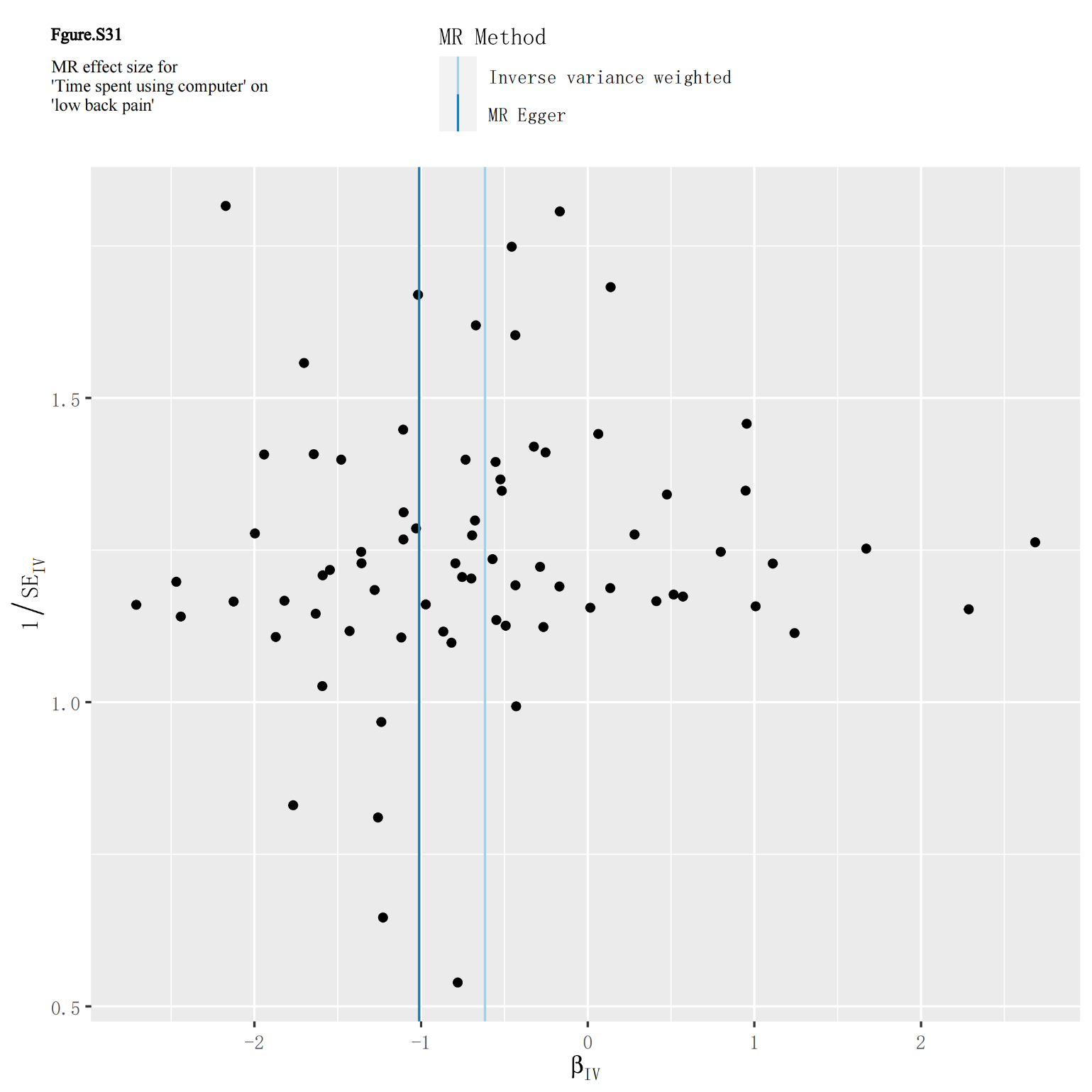

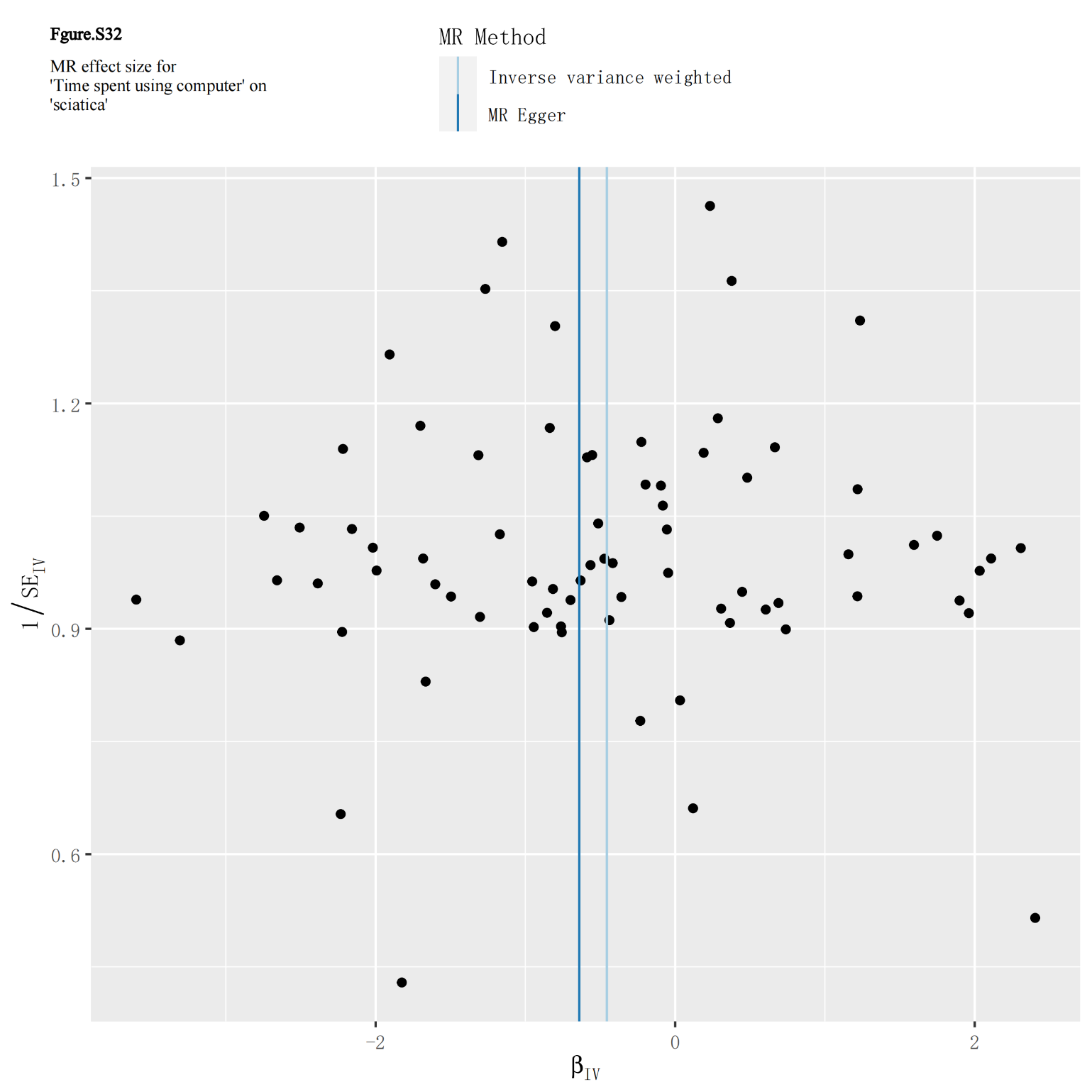

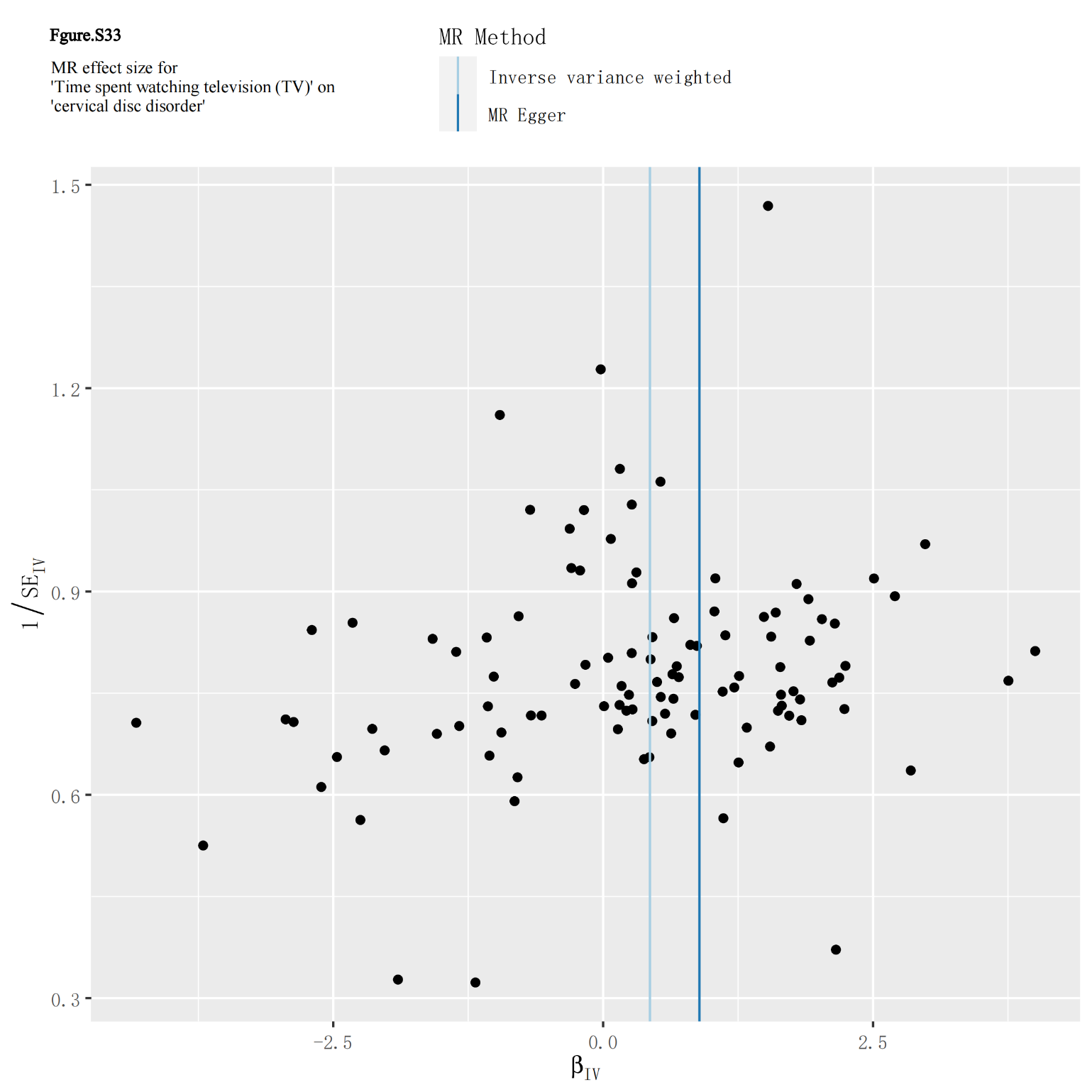

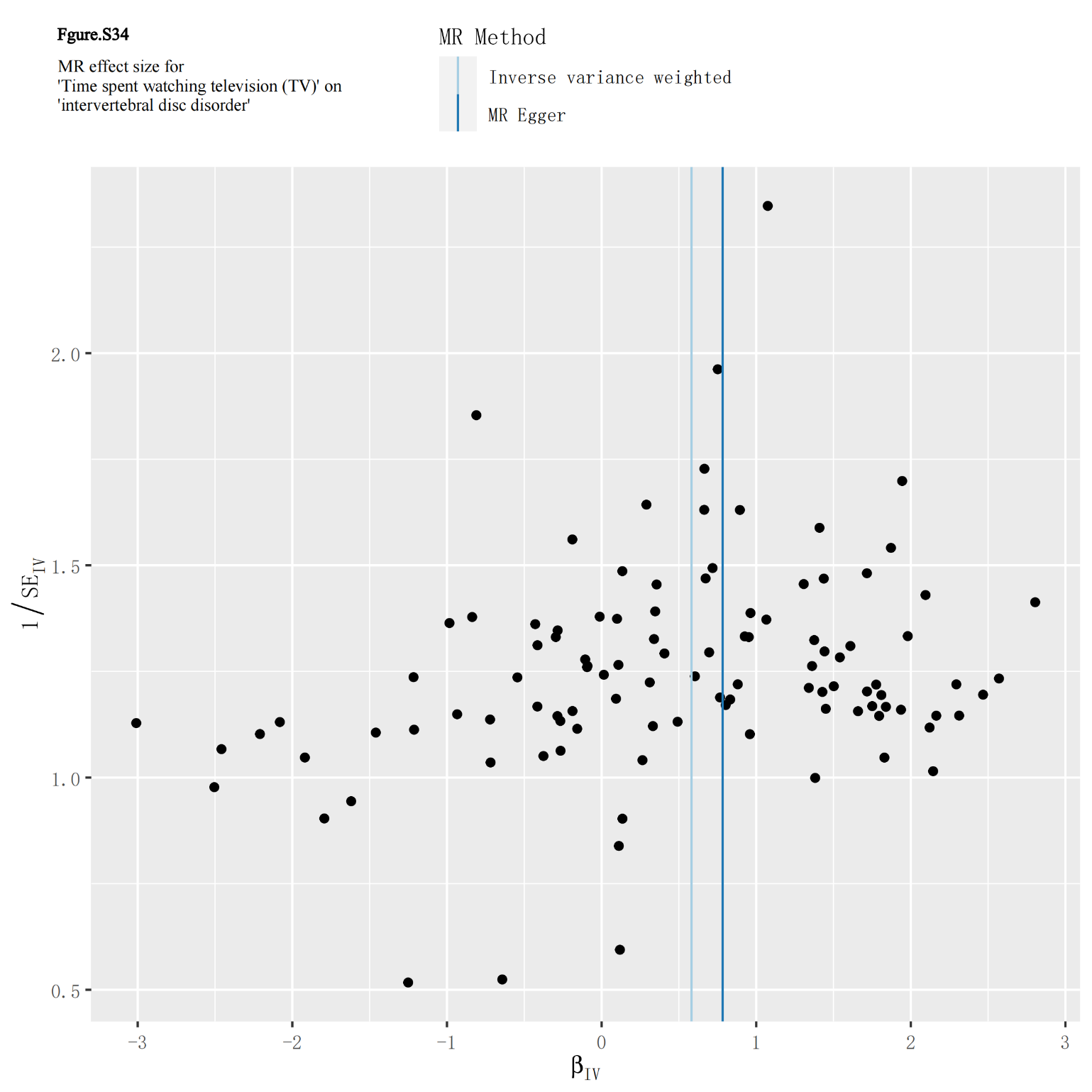

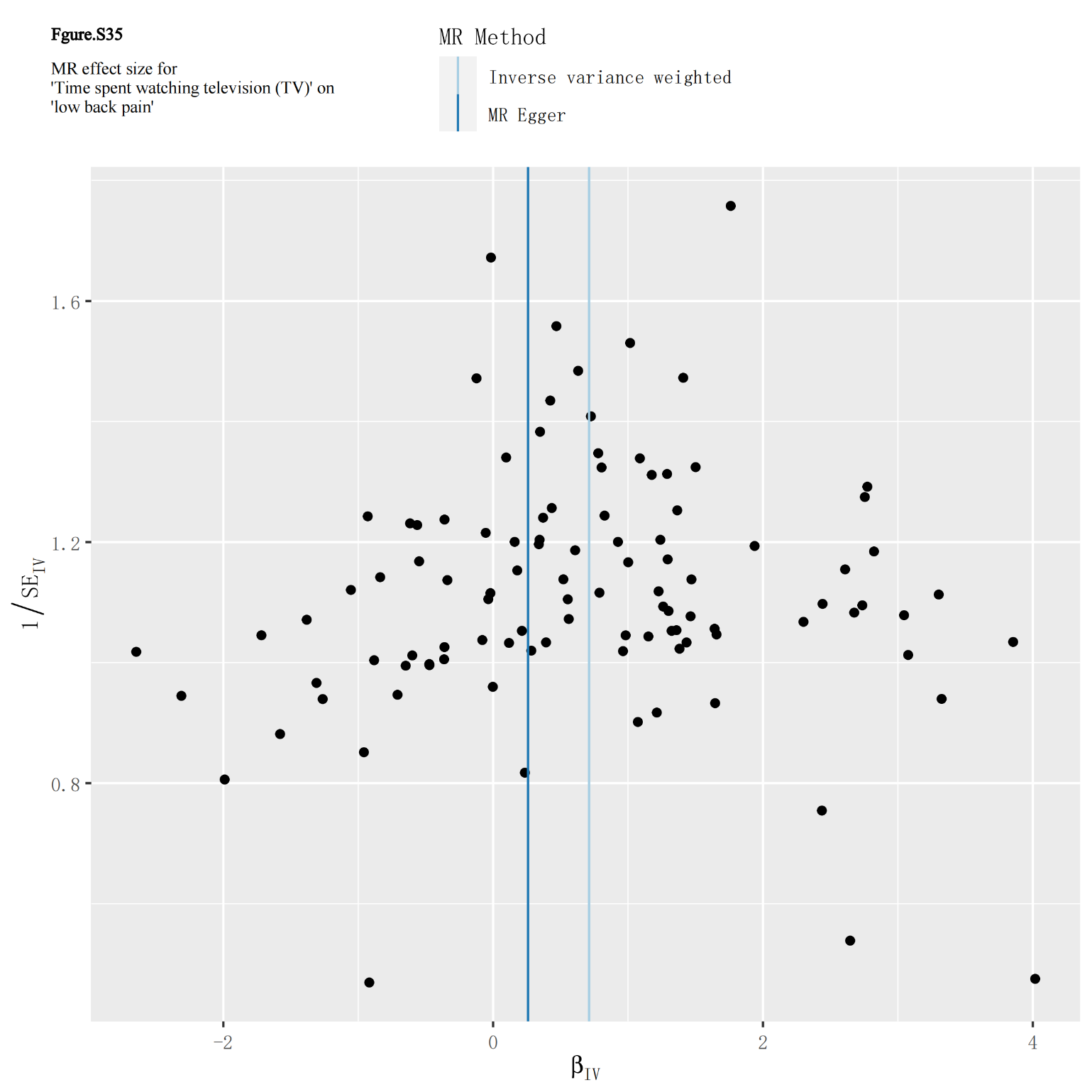

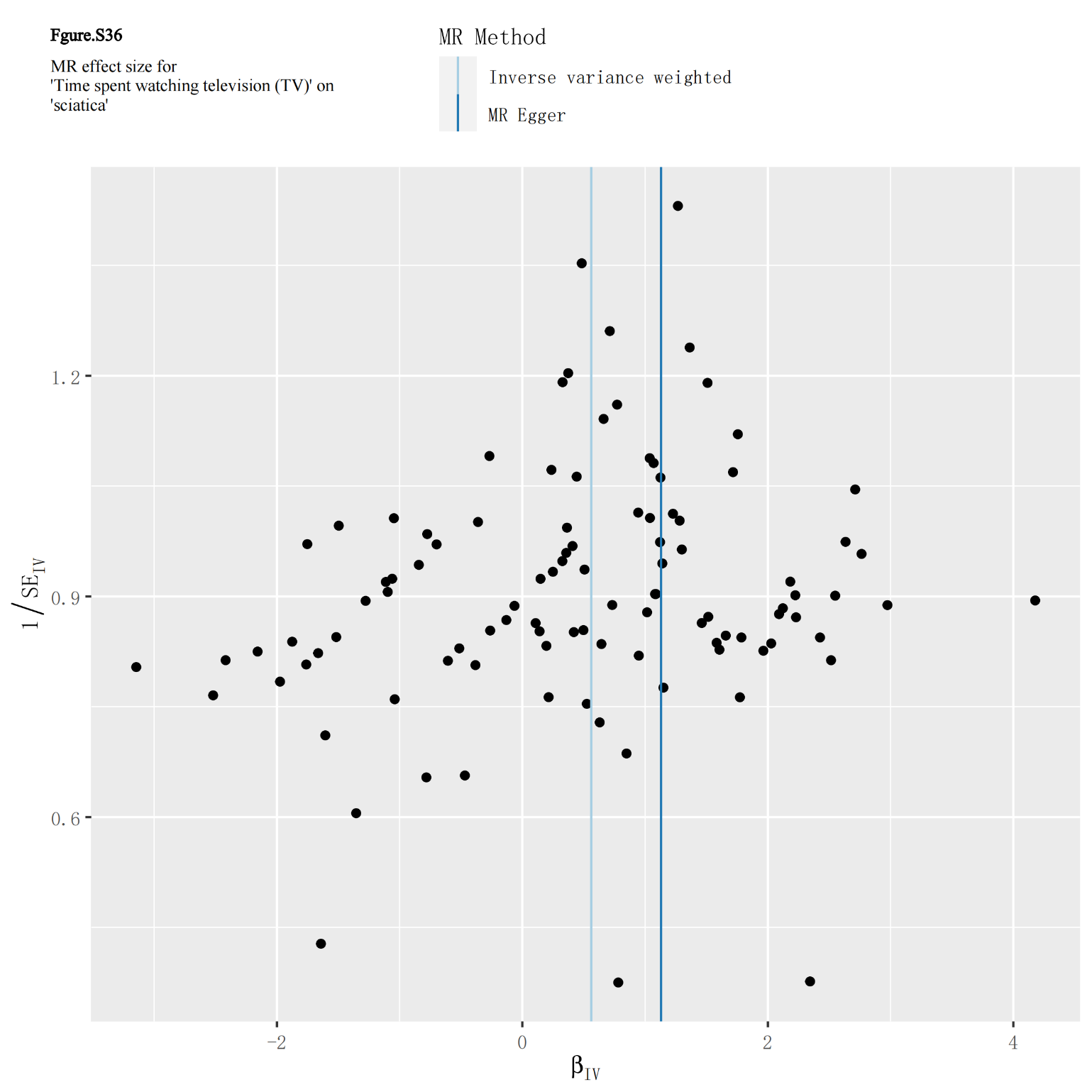

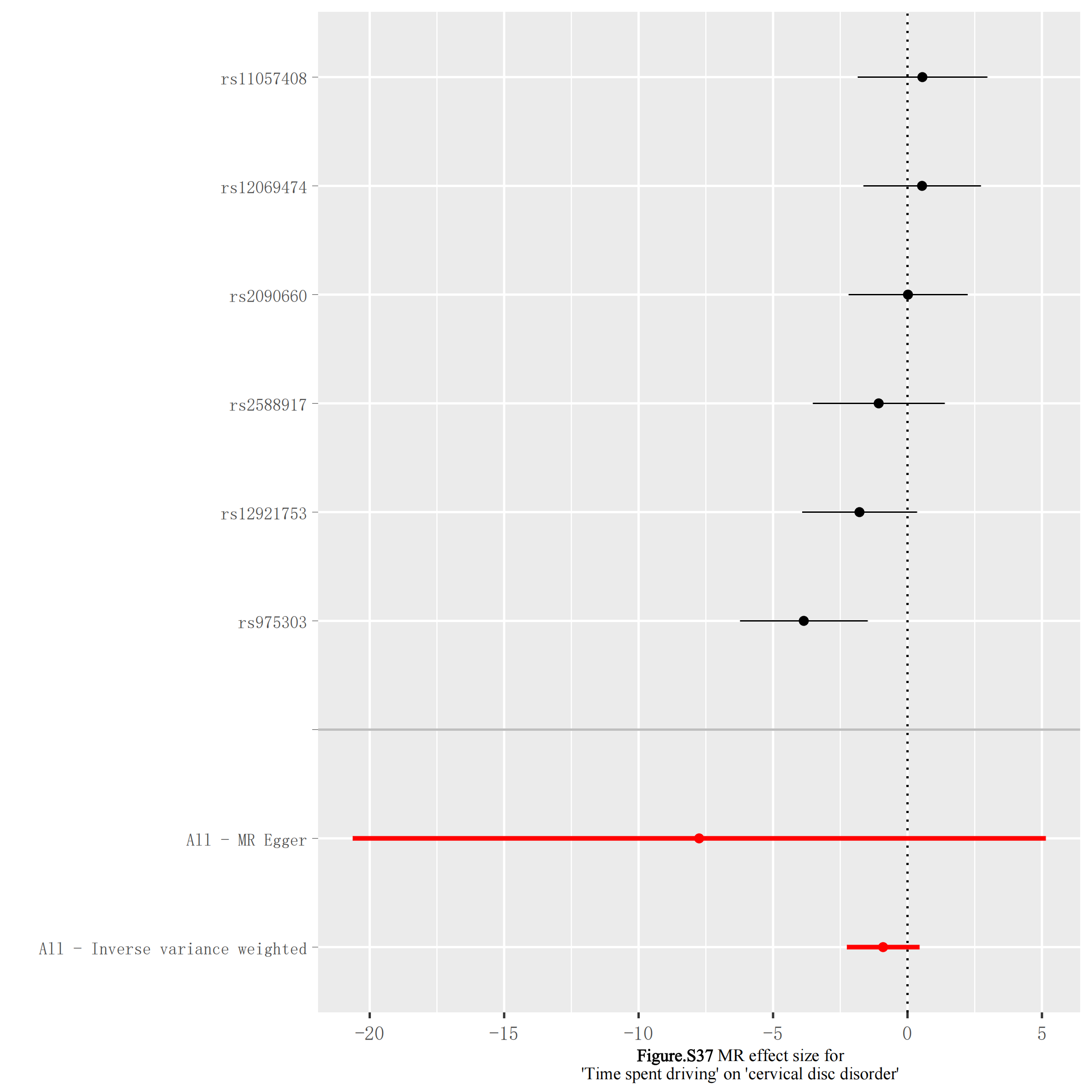

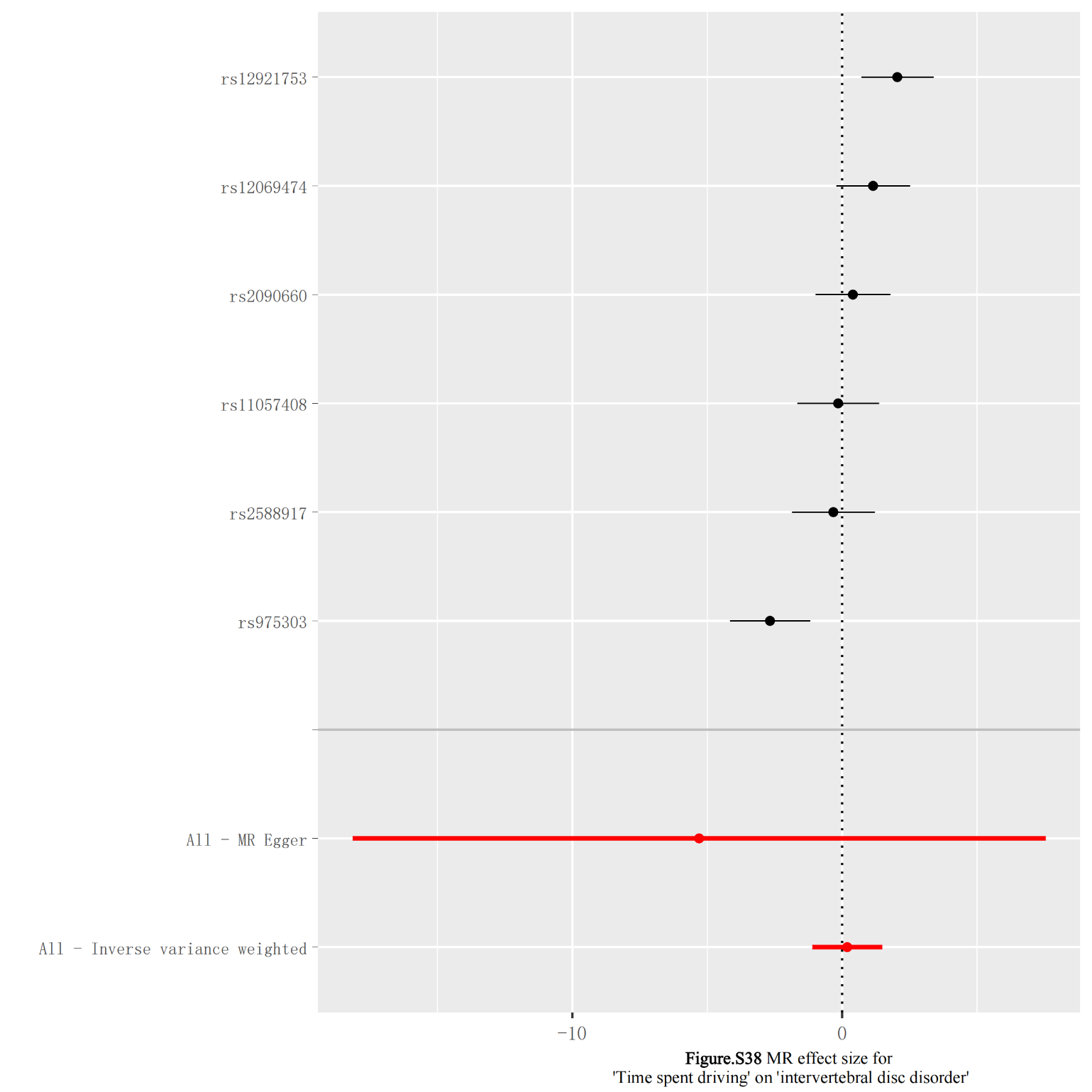

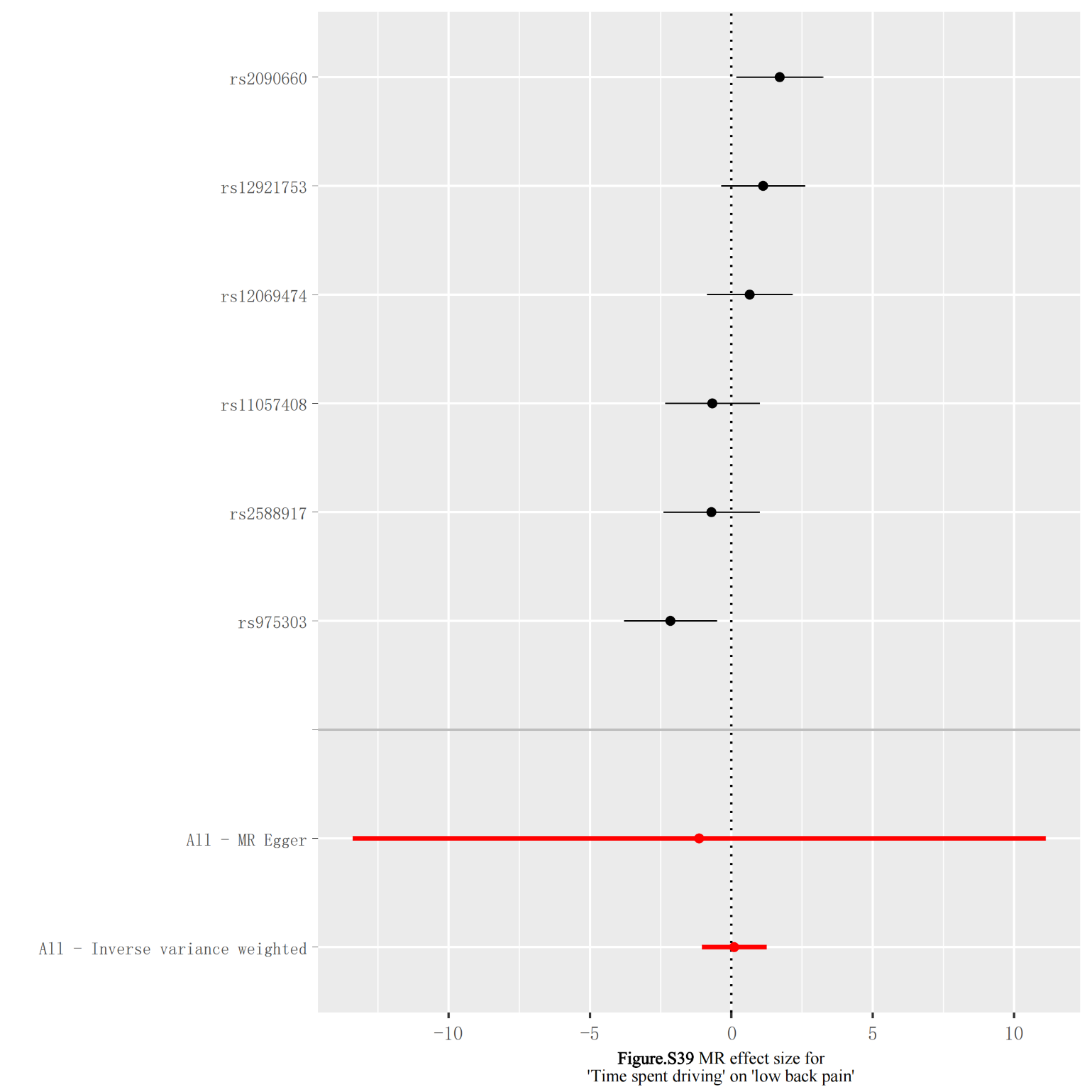

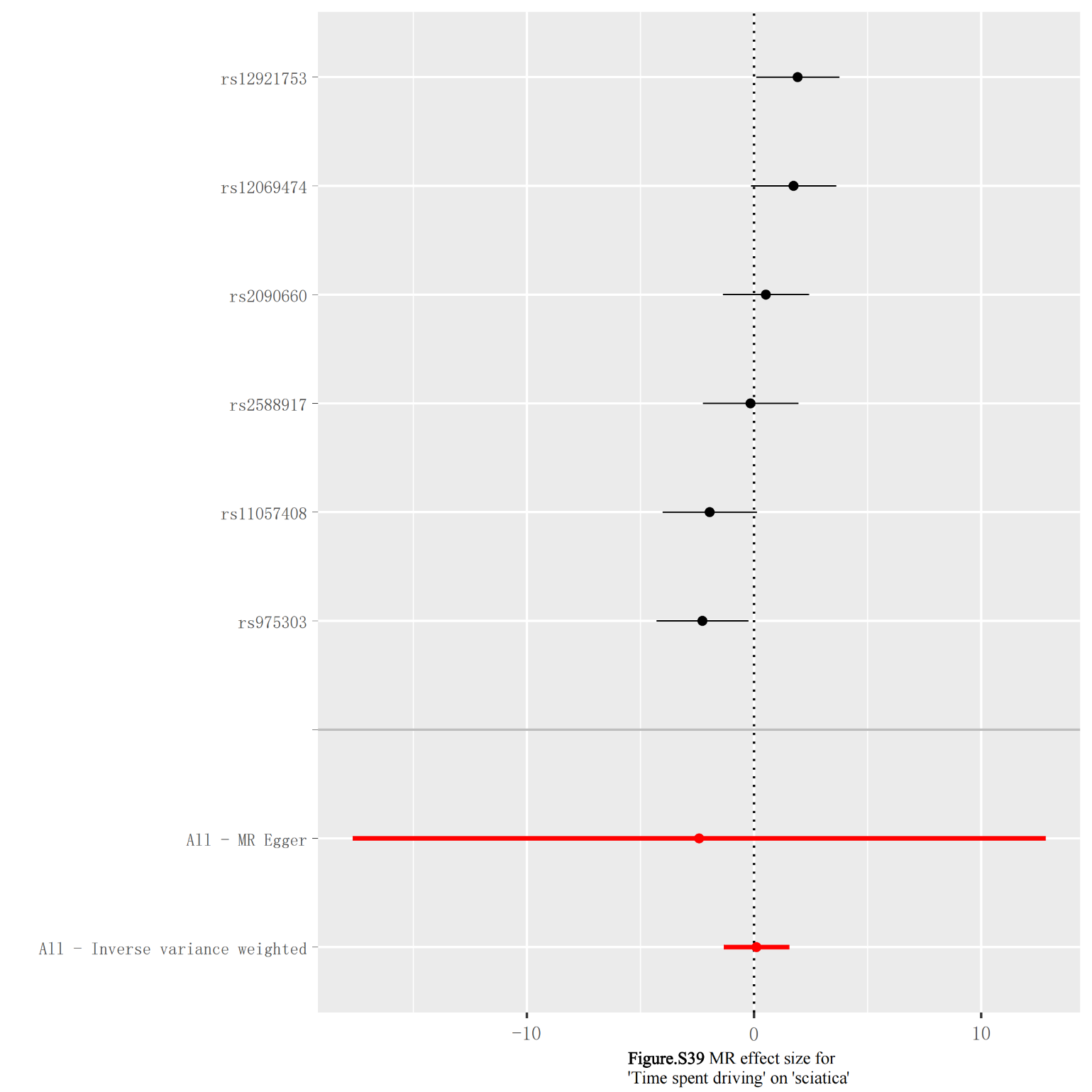

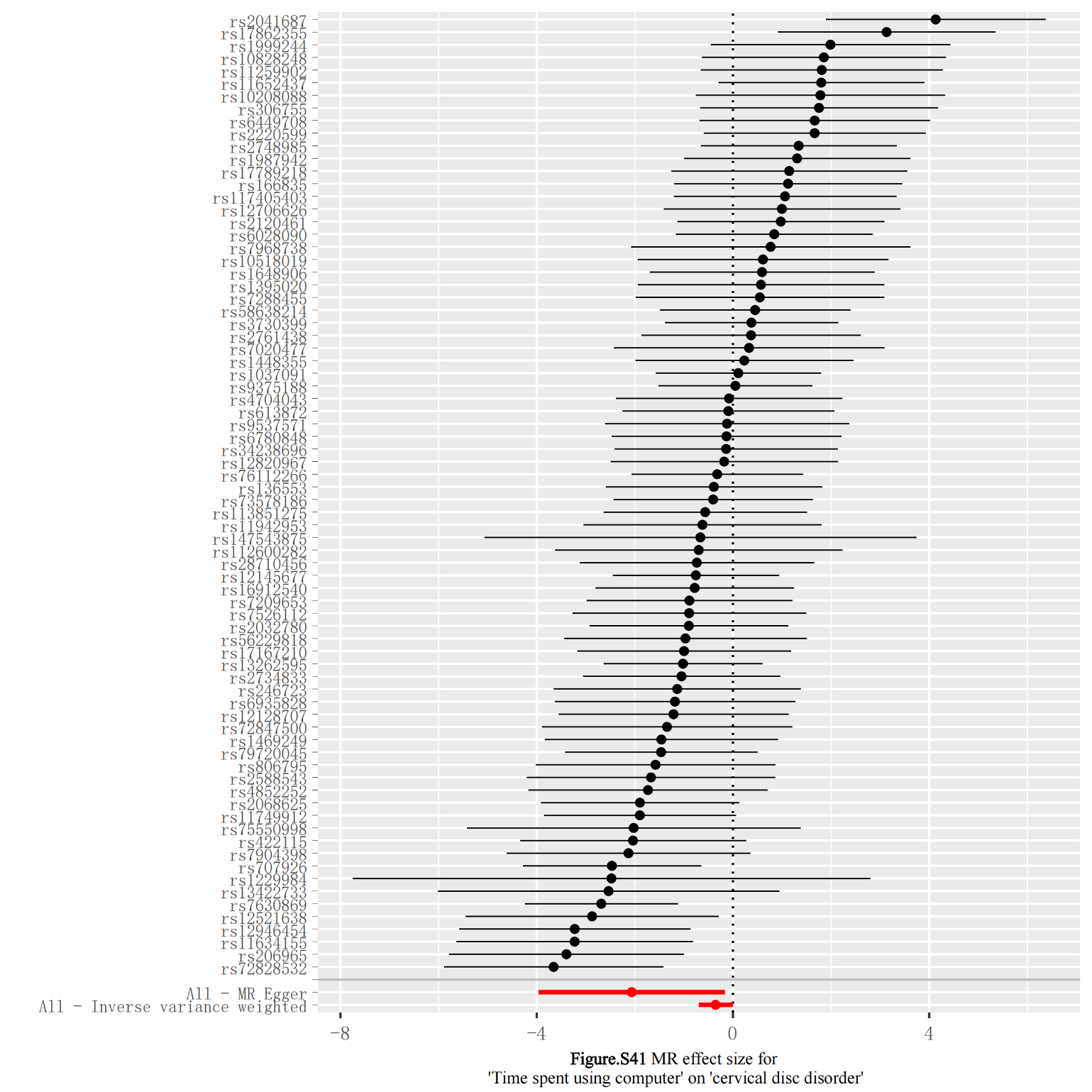

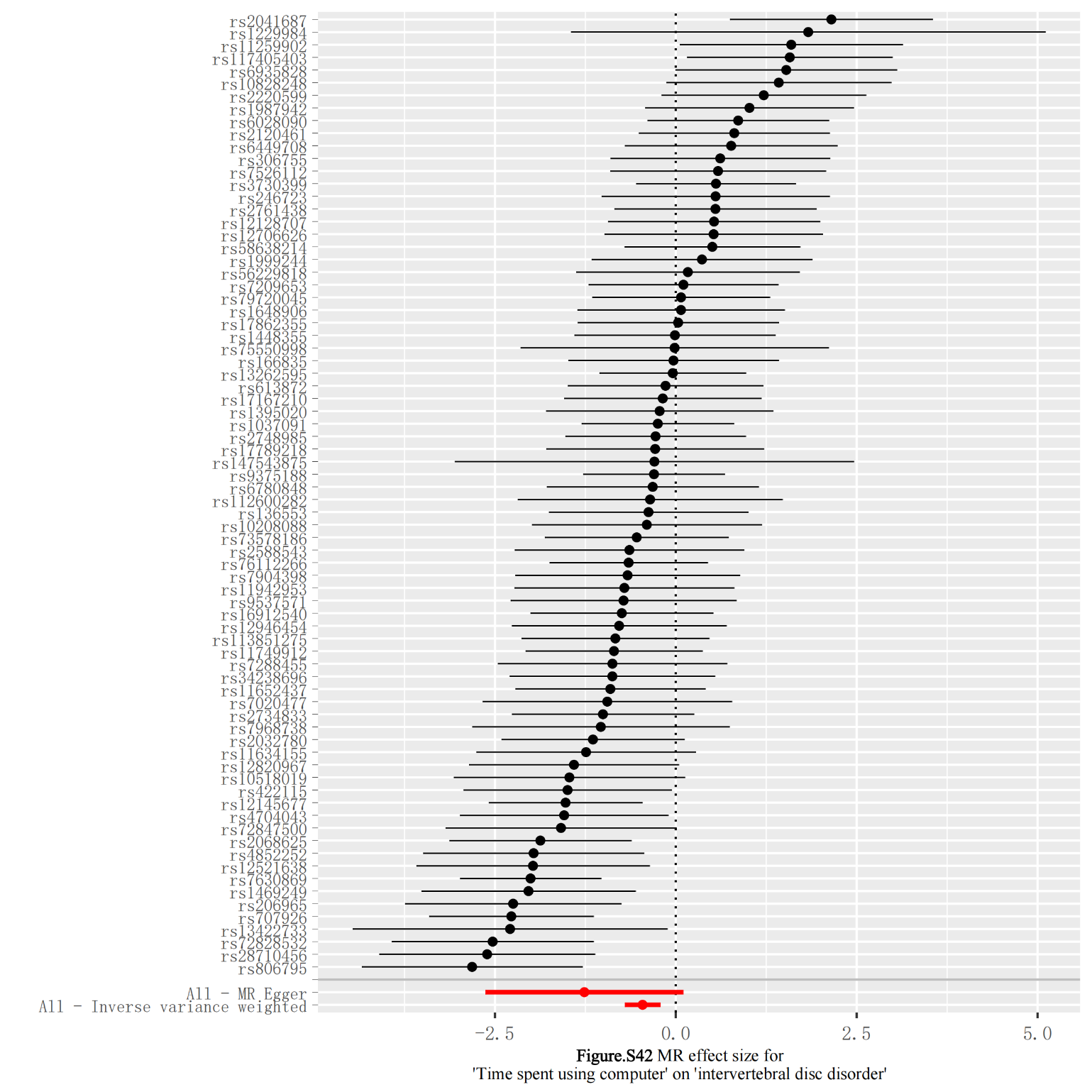

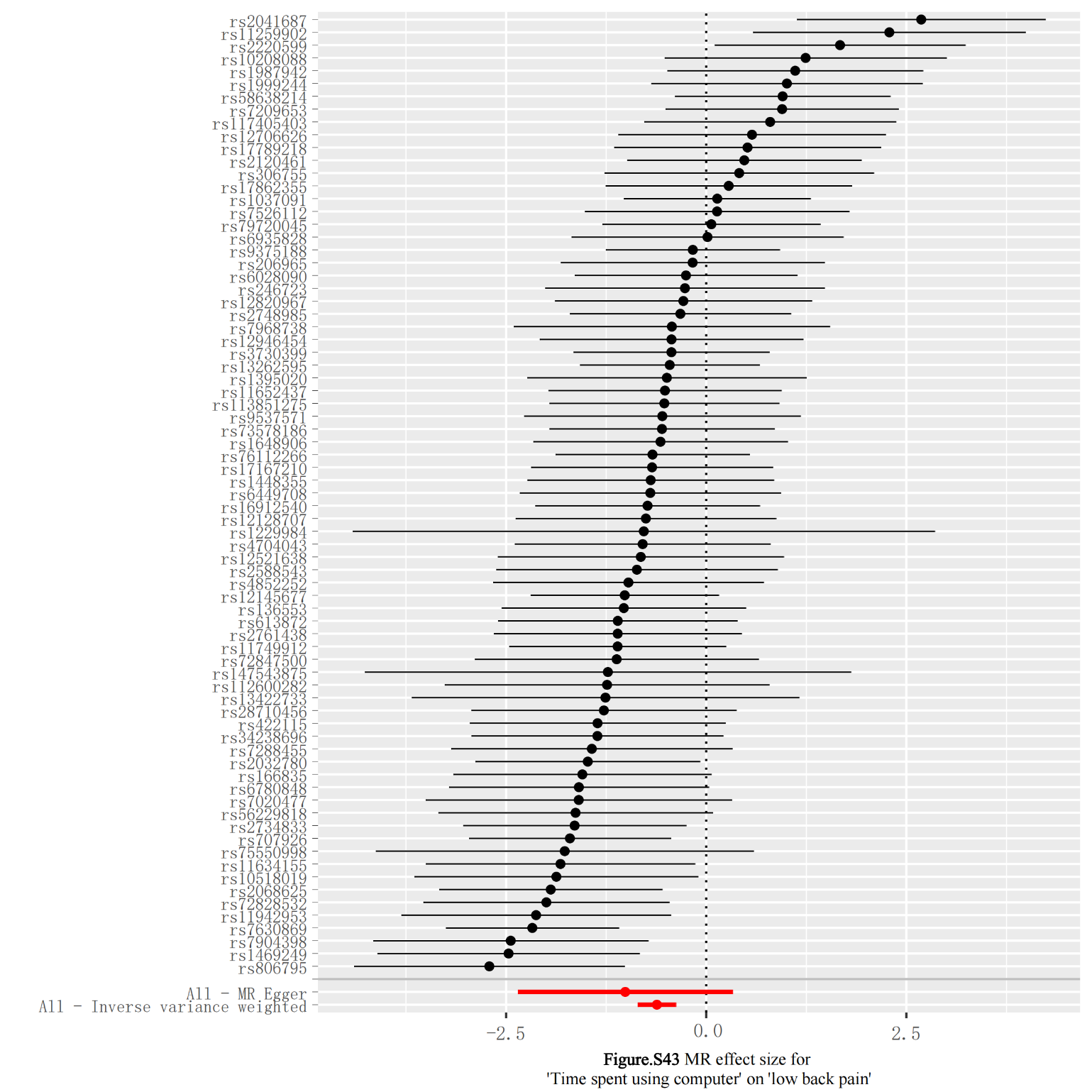

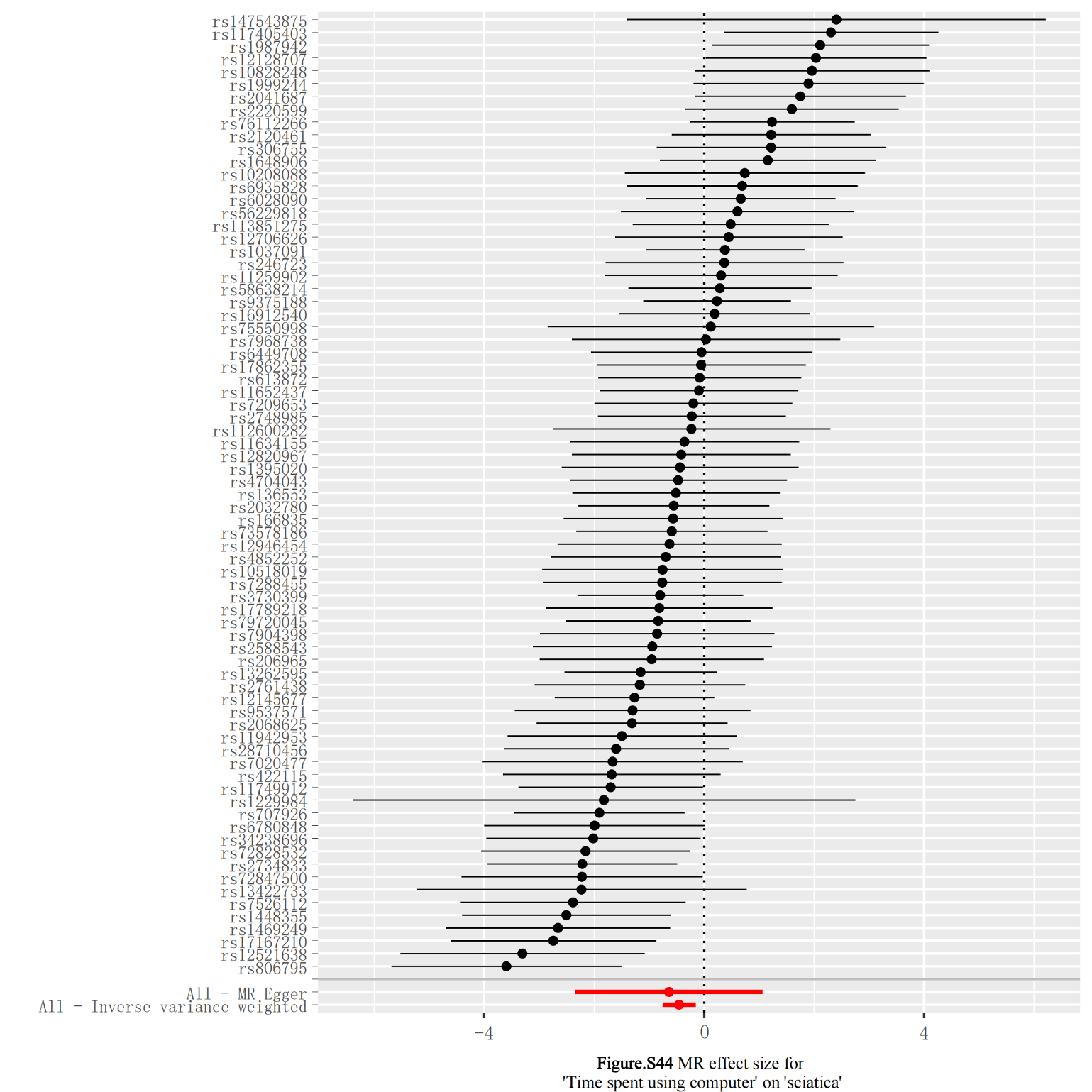

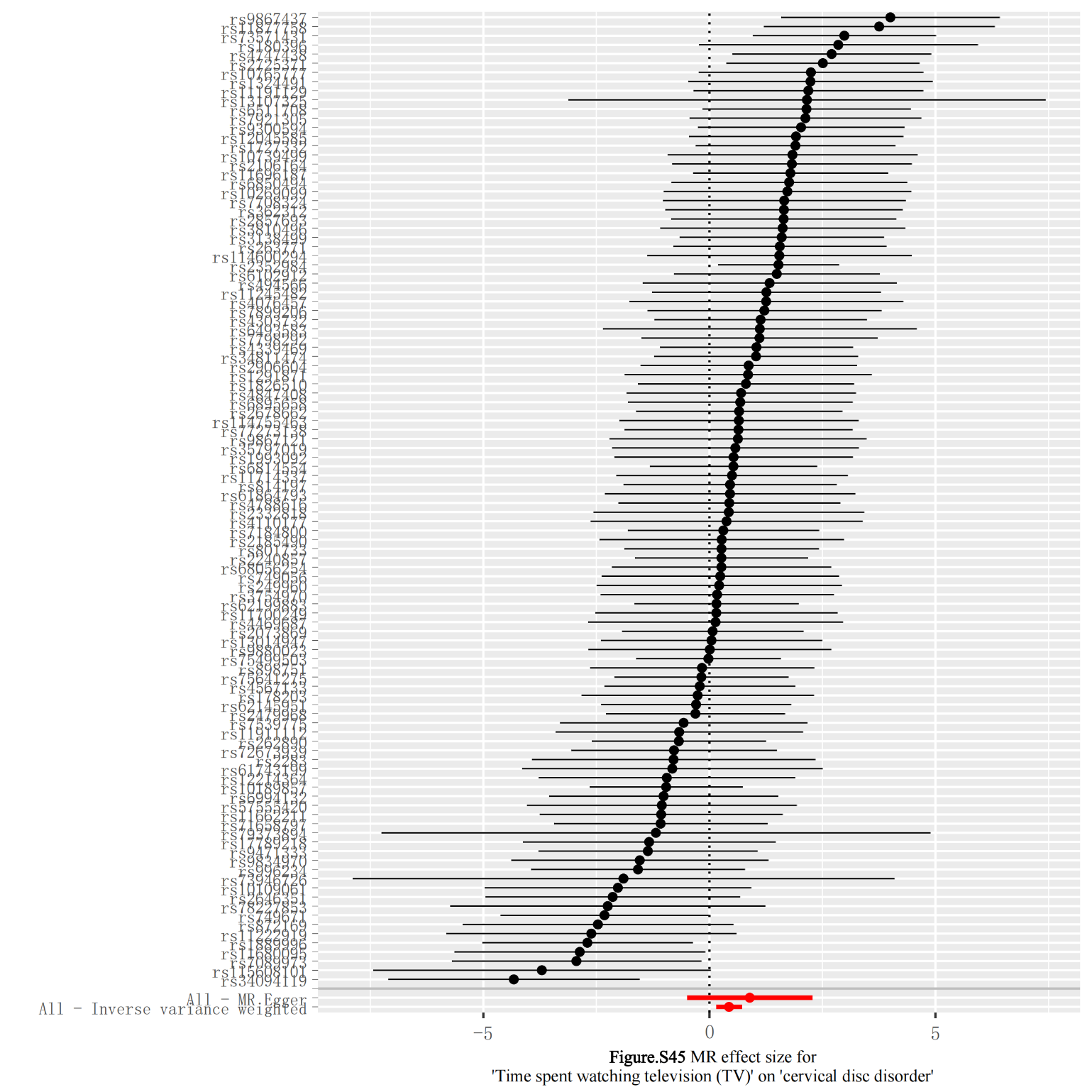

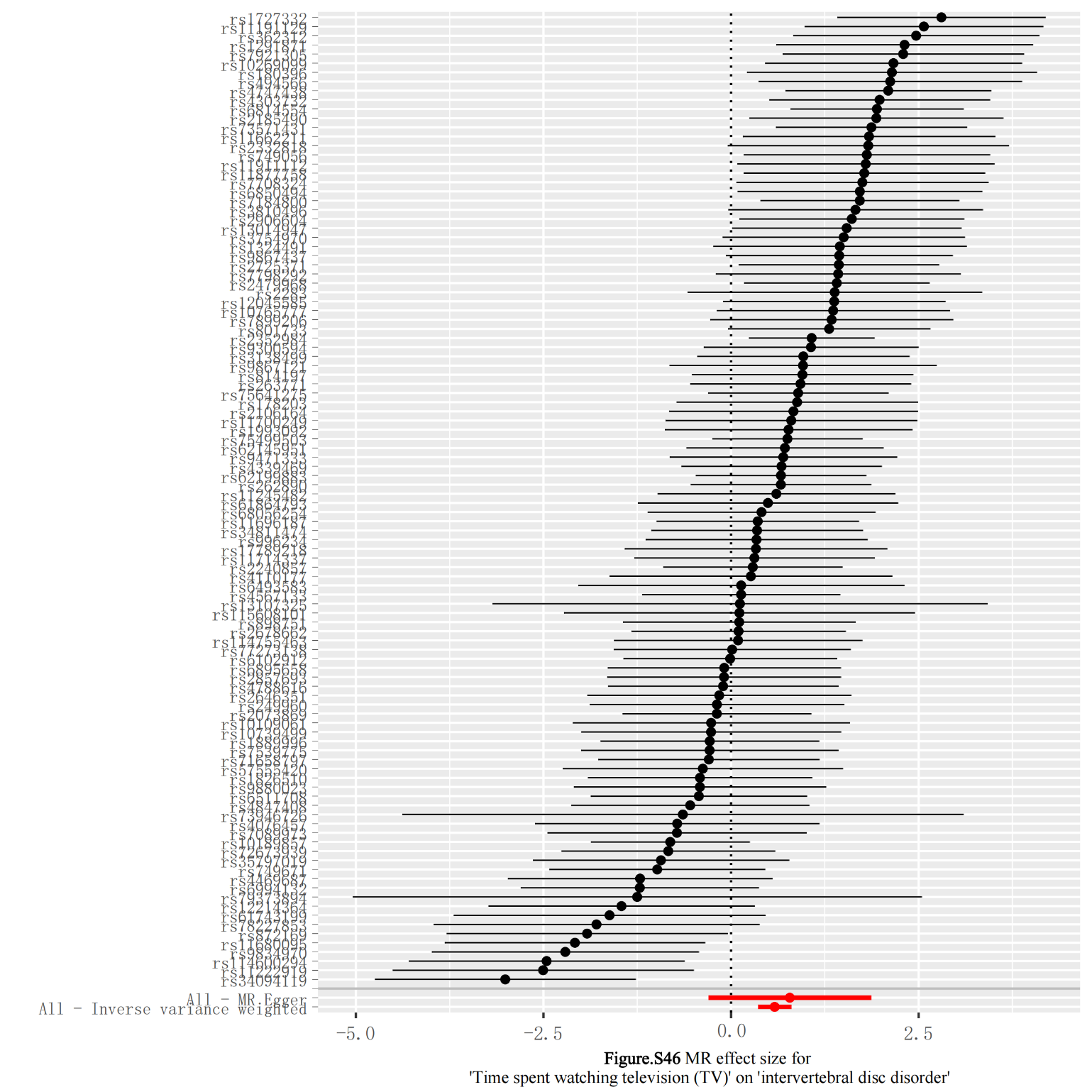

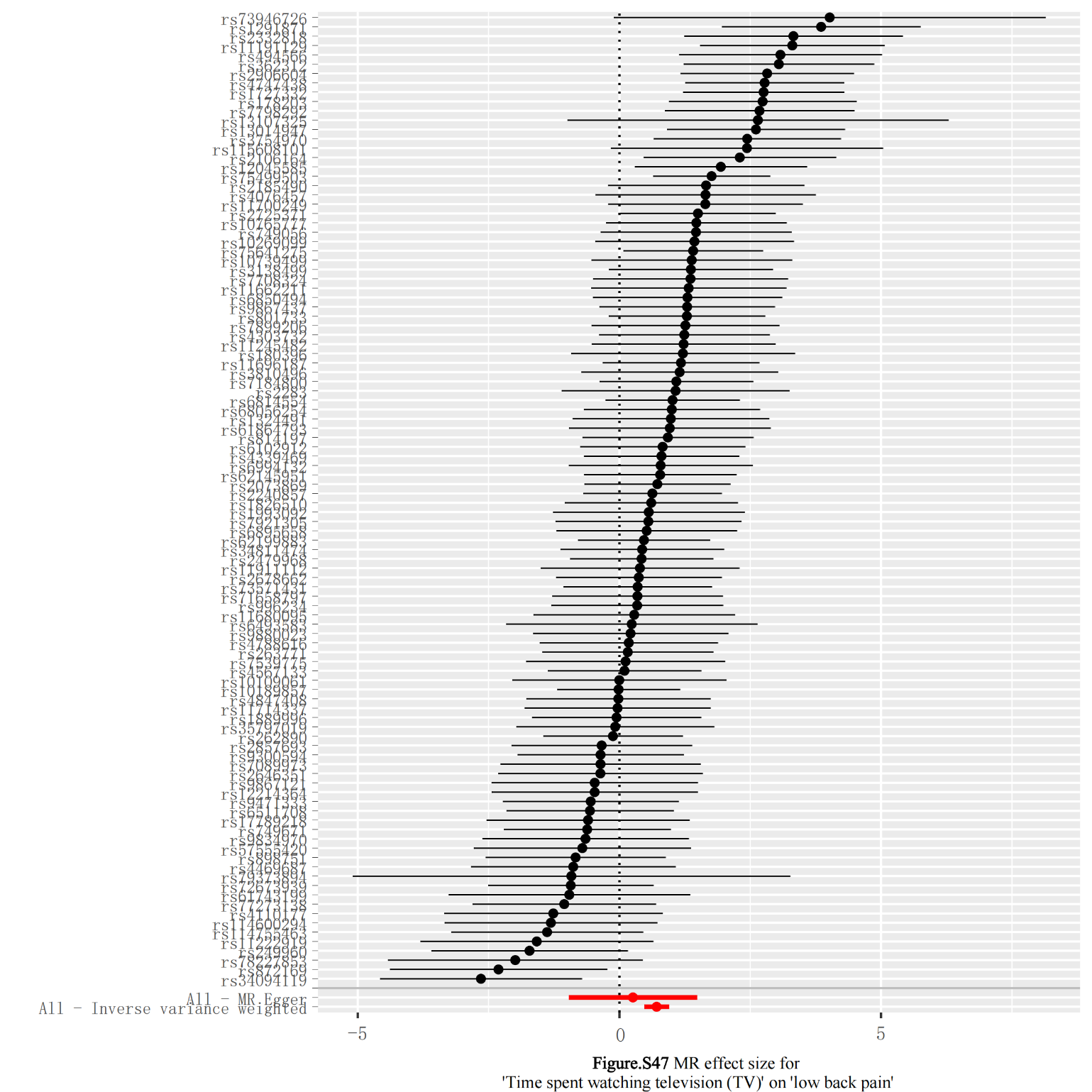

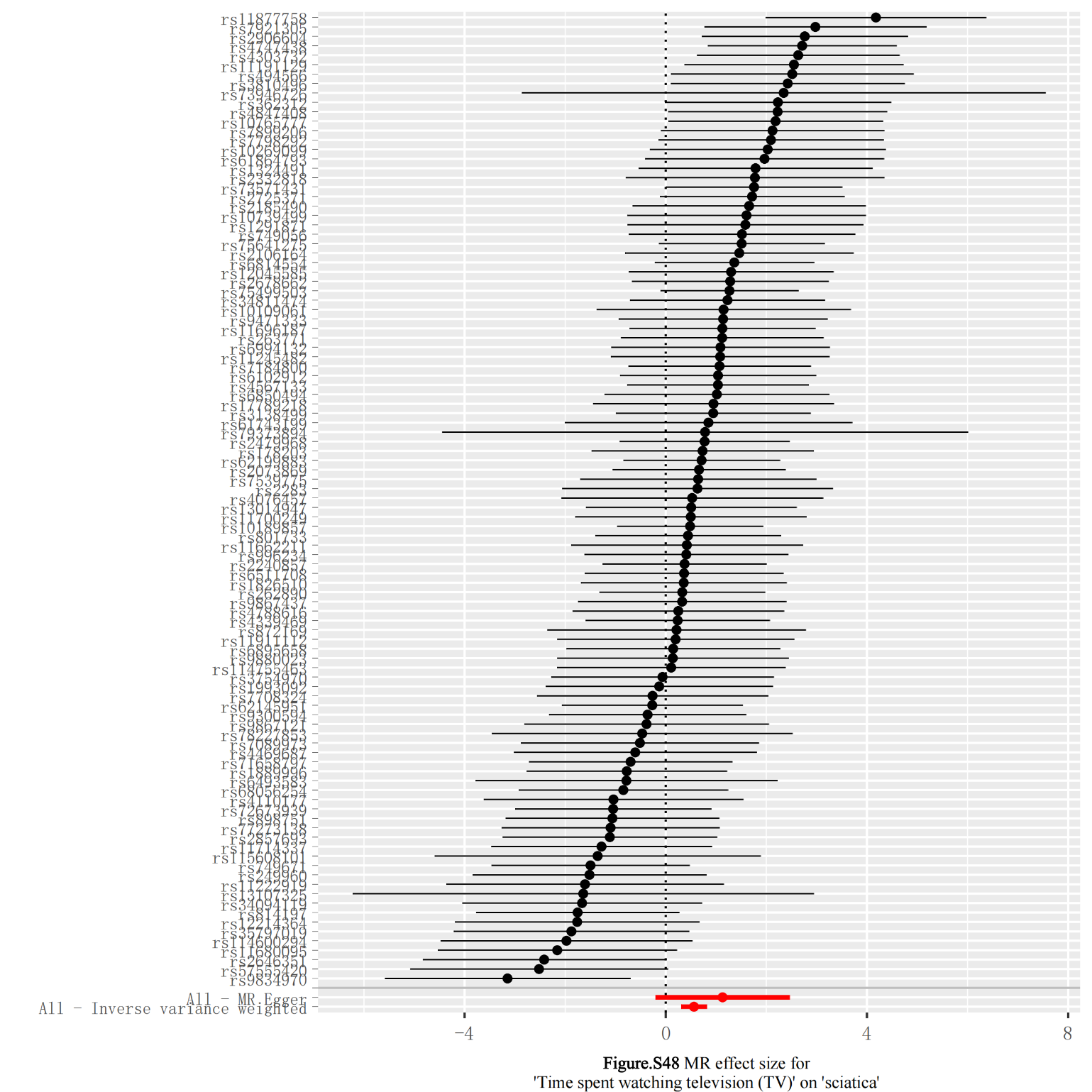

Supplement: Supplementary file 1 [file Data_Sheet_1.docx]
